# The Identification of Genetic Determinants of Methanol Tolerance in Yeast Suggests Differences in Methanol and Ethanol Toxicity Mechanisms and Candidates for Improved Methanol Tolerance Engineering

**DOI:** 10.3390/jof7020090

**Published:** 2021-01-27

**Authors:** Marta N. Mota, Luís C. Martins, Isabel Sá-Correia

**Affiliations:** 1 iBB—Institute for Bioengineering and Biosciences, Instituto Superior Técnico, Universidade de Lisboa, 1049-001 Lisbon, Portugal; marta.mota@tecnico.ulisboa.pt (M.N.M.); luismcmartins@tecnico.ulisboa.pt (L.C.M.); 2 Department of Bioengineering, Instituto Superior Técnico, Universidade de Lisboa, 1049-001 Lisbon, Portugal

**Keywords:** toxicogenomics, stress tolerance, toxicity mechanisms, methanol, ethanol, tolerance determinants, yeast robustness

## Abstract

Methanol is a promising feedstock for metabolically competent yeast strains-based biorefineries. However, methanol toxicity can limit the productivity of these bioprocesses. Therefore, the identification of genes whose expression is required for maximum methanol tolerance is important for mechanistic insights and rational genomic manipulation to obtain more robust methylotrophic yeast strains. The present chemogenomic analysis was performed with this objective based on the screening of the Euroscarf *Saccharomyces cerevisiae* haploid deletion mutant collection to search for susceptibility phenotypes in YPD medium supplemented with 8% (*v*/*v*) methanol, at 35 °C, compared with an equivalent ethanol concentration (5.5% (*v*/*v*)). Around 400 methanol tolerance determinants were identified, 81 showing a marked phenotype. The clustering of the identified tolerance genes indicates an enrichment of functional categories in the methanol dataset not enriched in the ethanol dataset, such as chromatin remodeling, DNA repair and fatty acid biosynthesis. Several genes involved in DNA repair (eight *RAD* genes), identified as specific for methanol toxicity, were previously reported as tolerance determinants for formaldehyde, a methanol detoxification pathway intermediate. This study provides new valuable information on genes and potential regulatory networks involved in overcoming methanol toxicity. This knowledge is an important starting point for the improvement of methanol tolerance in yeasts capable of catabolizing and copying with methanol concentrations present in promising bioeconomy feedstocks, including industrial residues.

## 1. Introduction

Methanol is a promising feedstock alternative to sugar-based raw materials for the bioproduction of fuels, specialty chemicals, polymers, and other value-added products due to its abundance and relatively low cost [1,2,3,4]. Methanol is also the major impurity in crude glycerol, reaching relatively high levels that can vary considerably from batch to batch and, although it can be removed by evaporation, this process is energy demanding [5,6]. Therefore, the utilization of methanol as co-substrate by methanol-tolerant methylotrophic yeasts would increase the feasibility of bioprocesses that use crude glycerol as substrate [5]. Methanol is also present, at relatively low concentrations, in hydrolysates from pectin-rich agro-industrial residues given that the D-galacturonic acid monomers are methyl-esterified in different positions [7,8,9]. Differently from methylotrophic yeast species, the preferred yeast cell factory *Saccharomyces cerevisiae*, is not able to use methanol as sole carbon source but there are successful examples of *S. cerevisiae* metabolic engineering for direct methanol utilization [2,10,11]. Although being a promising carbon source for metabolically competent yeast strains, methanol toxicity can limit the productivity of methanol-based biomanufacturing [3]. For this reason, the identification of genes/proteins whose expression is required for maximum tolerance to methanol in the model yeast species *S. cerevisiae* is important for enlightening the mechanisms underlying methanol toxicity in methylotrophic yeasts and in other eukaryotes as well as for guiding the development of more robust yeast strains, in particular methylotrophic yeast strains [3]. Genome-wide approaches have been enabling a holistic view and a deeper understanding of the molecular mechanisms and signaling pathways involved in the global response and adaptation of yeasts to sublethal concentrations of toxicants by allowing the identification of genes and pathways involved in the toxicological response and required for maximum tolerance [12,13]. The genome-wide identification of genes that are determinants of tolerance to methanol is a first step to allow the improvement of yeast robustness and the objective of the present study. Such chemogenomic analysis, using a *S. cerevisiae* deletion mutant collection, has been explored before to identify genes required for maximum tolerance to a variety of relevant chemical stresses including compounds of biotechnological, agronomical and pharmaceutical interest [12,13].

The methanol detoxification pathway in *S. cerevisiae* involves two reactions: (i) the oxidation of methanol to formaldehyde carried out by alcohol dehydrogenases, and (ii) the oxidation of formaldehyde to formic acid catalyzed by an aldehyde dehydrogenase [14]. The genome-wide response of yeast to methanol, based on transcriptomic analyses, was reported in two studies. Results suggest that the major cellular targets for methanol and formaldehyde toxicity are membrane structure and proteins, respectively [14], and that the response to methanol also includes the up-regulation, at different levels and depending on the yeast strain, of genes of mitochondrial and peroxisomal metabolism, alcohol and formate dehydrogenation, glutathione metabolism, at different levels, [15]. The screening of the same yeast deletion mutant collection used in our study was carried out for the identification of formaldehyde tolerance determinants [16,17]. Among them, DNA repair mechanisms were found to underlie formaldehyde tolerance, consistent with the alkylating activity of this compound [16,17]. Concerning formic acid, produced in the last step of methanol detoxification in yeast [14], another chemogenomic analysis performed in our lab, indicates an enrichment of tolerance genes involved in intracellular trafficking and protein synthesis, cell wall and cytoskeleton organization, carbohydrate metabolism, lipid, amino acid and vitamin metabolism, response to stress, chromatin remodeling, transcription, and internal pH homeostasis [18]. This study also confirms the involvement of the Haa1 transcription factor and the Haa1-regulon in the tolerance to formic acid [18], as described for acetic acid [19]. 

Although the available studies on the determinants and signaling pathways involved in yeast tolerance to methanol are scarce, several reports on tolerance to ethanol toxicity are available, in particular at the genome-wide level [20,21,22,23,24,25]. Due to the structural similarity of these short chain alcohols, the knowledge gathered for ethanol can be useful to understand methanol toxicity and tolerance in yeast. The screening of the yeast disruptome carried out in our laboratory for ethanol, using the same experimental methodology applied in the present work for methanol, identified as enriched in the obtained dataset genes associated with intracellular organization, biogenesis, and transport regarding the vacuole, the peroxisome, the endosome, and the cytoskeleton and the transcriptional machinery [22]. The clustering of the encoded proteins, based on their known physical and genetic interactions, highlighted the importance of the vacuolar protein sorting machinery, the vacuolar H (^+^)-ATPase (V-ATPase) complex, and the peroxisome protein import machinery [22]. Several plasma-membrane H^+^-ATPase and vacuolar H^+^-ATPase (V-ATPase) genes which are essential for maintaining the intracellular pH at physiological values [26,27,28,29], were also found in other genome-wide screenings as ethanol tolerance determinants [20,22,23,24,25]. As a lipophilic agent, ethanol leads to the perturbation of plasma membrane lipid organization and consequently to the increase of its non-specific permeability, disrupting membrane biological function as a matrix for proteins and, thus, affecting their activity [30,31]. Alterations of plasma membrane lipid composition is among the responses of the yeast cell considered useful to counteract those perturbations, namely at the level of sterol and unsaturated fatty acids composition and content [29,32,33]. Peroxisomal function is also responsible for yeast tolerance to ethanol with phospholipid biosynthesis since cells with abnormal peroxisomal function are unable to regulate the composition of membrane phospholipids, compromising membrane remodeling to overcome ethanol stress [22,25]. 

The first goal of the present study was to get insights into the global mechanisms underlying methanol toxicity through the identification of tolerance determinant genes by screening the entire Euroscarf haploid deletion mutant collection grown in YPD medium supplemented with 8% (*v*/*v*) methanol at 35 °C. This chemogenomic analysis was extended to an equivalent growth inhibitory concentration of ethanol of 5.5% (*v*/*v*) performed under identical experimental conditions to compare the mechanisms underlying methanol and ethanol toxicity and tolerance. This was considered essential because the various available genome-wide studies that allowed the identification of genetic determinants of ethanol tolerance were performed under different experimental conditions: in rich medium with ethanol concentrations of 7% (*v*/*v*) [23], 10% (*v*/*v*) [20,21] and 11% (*v*/*v*) [24] and in minimal medium supplemented with 8% (*v*/*v*) ethanol [22]. Moreover, the criteria used to identify genes that when deleted lead to ethanol susceptibility phenotypes varied. The results obtained in the present work indicate that, despite the similarities identified for a vast number of genetic determinants of tolerance to these two alcohols, DNA repair and membrane remodeling are among the more specific responses to counteract methanol toxicity. Results from this genome-wide search for genes that confer tolerance to methanol in *S. cerevisiae* can now be explored for the rational genetic manipulation of yeasts to obtain more robust strains capable to cope with stressing methanol concentrations, in particular of methylotrophic yeasts. 

## 2. Materials and Methods

### 2.1. Strains and Growth Media

The haploid parental strain *S. cerevisiae* BY4741 (MATa, *his3*Δ*1*, *leu2*Δ*0*, *met15*Δ*0*, *ura3*Δ*0*) and the collection of derived single deletion mutants were obtained from Euroscarf (Frankfurt, Germany). The screening of this collection and the growth curves shown were carried out in YPD medium containing, per liter, 2% (*w*/*v*) glucose (Merck, Darmstadt, Germany), 2% (*w*/*v*) yeast extract and 1% (*w*/*v*) peptone, both from BD Biosciences (Franklin Lakes, NJ, USA) acidified with HCl until pH 4.5. Solid media were prepared by addition of 2% (*w*/*v*) agar (Iberagar, Barreiro, Portugal). 

### 2.2. Genome-Wide Search for Yeast Determinants of Methanol or Ethanol Tolerance

To select the alcohol concentrations to be used for the disruptome assays, the parental strain *S. cerevisiae* BY4741 was tested for susceptibility to a range of methanol or ethanol concentrations. For that, yeast cells were cultivated for 10 hours in liquid YPD medium, followed by the inoculation in fresh liquid YPD medium (pH 4.5) and growth to a standardized OD_600nm_ of 0.5 ± 0.05. These exponentially growing culture was used to inoculate fresh liquid YPD medium (pH 4.5) supplemented with increasing alcohol concentrations (Merck, Darmstadt, Germany) at 35 °C, in 96-wells plates. Growth was followed for 36 h using a microplate reader set at OD_595nm_ (FilterMax F5 Microplate Reader; Molecular Devices). 

Based on the results of the above referred first screening, the entire BY4741 Euroscarf deletion mutant collection was screened for susceptibility to the selected concentrations of 8% (*v*/*v*) methanol or to 5.5% (*v*/*v*) ethanol, at 35 °C, in YPD medium (pH 4.5). For that, the parental and deletion mutant strains were cultivated for 16 h in YPD medium at 30 °C with 250 rpm orbital agitation, in 96-well plates. Using a 96-pin replica platter, the cell suspensions were spotted onto the surface of YPD solid medium supplemented, or not, with 8% (*v*/*v*) methanol or 5.5% (*v*/*v*) ethanol and incubated at 35 °C. Photographs were taken after 24 h of incubation for control plates (YPD medium) or 36–48 h in the presence of the alcohols. 

When observed, the susceptibility phenotype of each single deletion mutant was scored as (+) if the mutant strain showed, compared with the parental strain, a slight growth inhibition after the standardized incubation time, (++) if the growth was moderately inhibited compared to the parental strain, and (+++) if no growth was observed after 48h of incubation (Supplementary Material, Figure S1). 

The eventual over- or under- representation of Gene Ontology (GO) biological process terms related with the physiological function of the genes found to be required for maximum tolerance to methanol was determined using the PANTHER Classification System (http://pantherdb.org); over-representation of functional categories was considered significant for a *p*-value < 0.05 and this analysis was complemented using the information available at *Saccharomyces* Genome Database (SGD) (http://www.yeastgenome.org).

### 2.3. Growth Curves of Selected Deletion Mutants under Methanol-Induced Stress

The susceptibility of the parental strain and selected deletion mutants was compared in liquid YPD medium in Erlenmeyer flasks or onto solid YPD medium in Petri dishes, both at pH 4.5. 

For the spot assays in solid YPD medium, exponentially-growing yeast cell suspensions (OD_600nm_ of 0.5 ± 0.05) were diluted to an OD_600nm_ of 0.25 ± 0.005 (a) and this suspension was used to prepare 1:5 (b), 1:25 (c), 1:125 (d), and 1:625 (e) serially diluted suspensions. Four microliters of each cell suspension were spotted onto YPD solid medium either or not supplemented with increasing concentrations of methanol (0, 8, 10, 12, or 14% (*v*/*v*)). Susceptibility phenotypes were observed after 48 h of incubation at 35 °C.

For susceptibility to methanol testing by growth in liquid YPD medium, a mid-exponential cell suspension was used to inoculate 50 mL of YPD medium (pH 4.5) in 100 mL flasks, either or not supplemented with 8% (*v*/*v*) methanol, with an initial OD_600nm_ of 0.1 ± 0.05. Cell cultivation was performed at 35 °C, with orbital agitation (250 rpm) and growth followed based on culture OD_600nm_.

## 3. Results

### 3.1. Selection of Methanol and Ethanol Concentrations for the Chemogenomic Analysis

**Figure 1:**
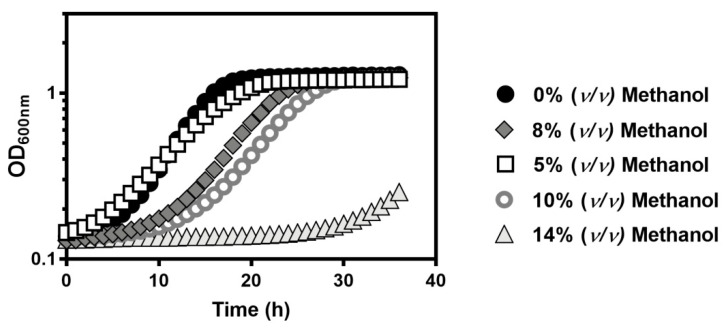
Comparison of the growth curves of *S. cerevisiae* BY4741 in YPD medium (pH 4.5) supplemented or not (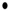) with 5% (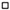), 8% (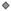), 10% (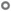), or 14% (*v*/*v*) (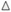) of methanol. Growth was followed in a microplate reader by measuring the optical density at 595 nm of a 96-well plate incubated at 35 °C with orbital agitation, for 36 h.

To select the appropriate methanol and ethanol concentrations to perform the planned chemogenomic analysis, the growth curves of the parental strain *Saccharomyces cerevisiae* BY4741 were compared in YPD liquid medium supplemented or not with 5%, 8%, 10% and 14% (*v*/*v*) methanol, at pH 4.5 and 35 °C during 36 h in a 96-wells plate (Figure 1). The incubation time was fixed in 36 h to limit alcohol evaporation. Only 8% and 10% (*v*/*v*) of methanol were considered suitable concentrations since 14% (*v*/*v*) did not allow detectable growth after 30 h of incubation and 5% (*v*/*v*) did not significantly affect the growth profile.

**Figure 2:**
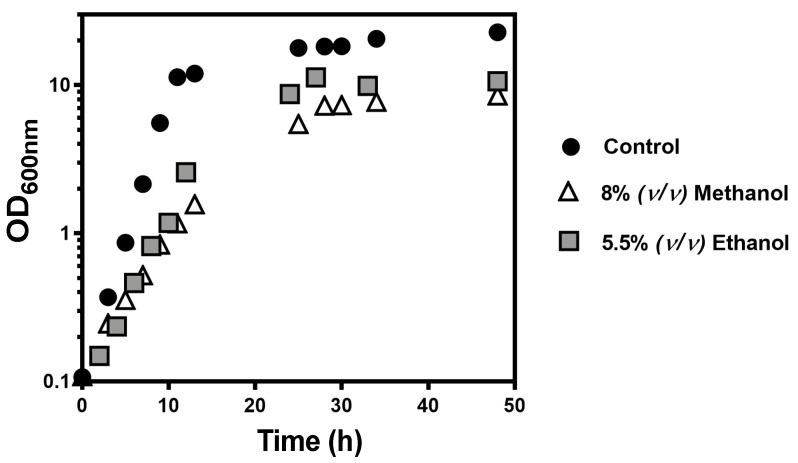
Effect of equivalent methanol and ethanol concentrations in the growth curves of *S. cerevisiae* BY4741. Cells were cultivated in liquid YPD medium (pH 4.5) supplemented, or not (circles), with 8% (*v*/*v*) methanol (triangles) or 5.5% (*v*/*v*) ethanol (squares) at 35 °C with orbital agitation (250 rpm). Growth was followed based on culture optical density at 600 nm (OD_600nm_) and the growth curves shown are representative of at least three independent experiments.

An inhibitory ethanol concentration equivalent to 8% (*v*/*v*) of methanol was also selected using the same methodology and the selected value was 5.5% (*v*/*v*) (Figure 2). 

### 3.2. Identification of Genes Required for Methanol and Ethanol Tolerance at a Genome-Wide Scale

**Figure 3:**
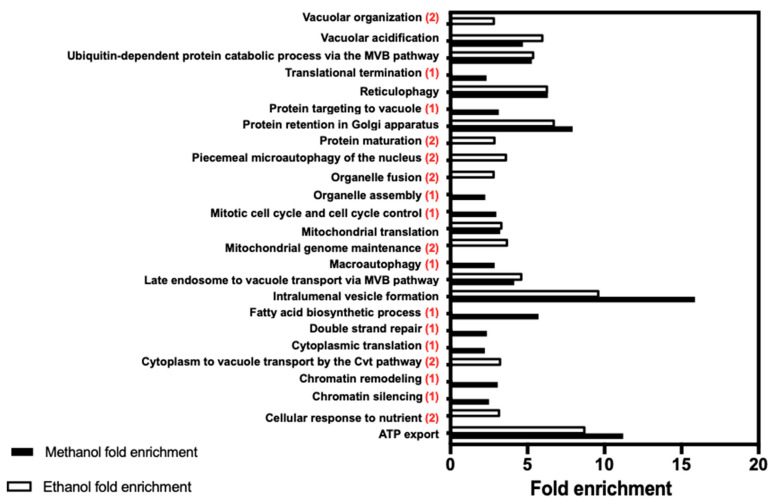
Biological functions enriched in the datasets of genes required for yeast tolerance to methanol (black bars) or ethanol (white bars). Genes listed in Supplementary Material, Tables S1 and S2, were clustered according to their biological process GO assignments using the PANTHER Classification System (http://pantherdb.org), and functional categories were considered to be over-represented if *p*-value < 0.05. The fold enrichment is calculated by dividing the number of genes present in the input dataset by the total number of genes of yeast genome expected to belong to specific functional class. (1) indicates the biological functions that are only enriched in the methanol dataset while (2) indicates those only enriched in the ethanol dataset. The biological functions with no indication are enriched in both datasets.

Approximately 5100 deletion mutants were tested for susceptibility to 8% (*v*/*v*) methanol compared with the parental strain and the generated dataset compared with the dataset obtained, under the same experimental conditions, for 5.5% (*v*/*v*) of ethanol. Four hundred and two of those mutants were found to be more susceptible to methanol than the parental strain, 81 of these mutants showing full growth inhibition (+++), 170 showing a moderate growth inhibition (++), and 151 a minor growth inhibition (+). The full list of genes is available in the Supplementary Material, Table S1. No genes that when deleted leads to higher methanol tolerance, were detected. The full list of genes required for ethanol tolerance (Supplementary Material Table S2) includes 445 genes, 110 of them leading to growth abrogation when deleted under the experimental conditions tested. Based on the biological function of the genes required for maximum methanol or ethanol tolerance, they were clustered according to the PANTHER Classification System (http://pantherdb.org). The fold enrichment of different functional classes in the two datasets (*p*-value < 0.05), is shown in Figure 3.

A more detailed discussion on selected methanol tolerance genes, in particular those belonging to different enriched functional classes, follows.

#### 3.2.1. Genes Involved in DNA Repair and Mitotic Cell Cycle

Results from the performed chemogenomic analysis strongly suggest that DNA is a main specific molecular target of methanol toxicity since several genes related with DNA repair were found to be enriched in the methanol dataset but not in the ethanol dataset. Specifically, eight radiation sensitive (*RAD*) genes are relevant for overcoming methanol-induced deleterious effects given that the corresponding deletion mutants exhibited a marked susceptibility phenotype (Table 1). These genes are involved in DNA repair mechanisms, in particular in non-homologous end joining and base excision repair (*RAD27*), homologous recombination (*RAD51* and *RAD57*), post-replication repair (*RAD5*, *RAD6* and *RAD18*) and nucleotide excision repair (*RAD33*). The genes *MET18*, *MRE11*, and *SGS1*, involved in the biological functions described in Table 1, also play a role in DNA repair mechanisms. Some of the mentioned genes involved in DNA repair also participate in mitotic cell cycle (*MRE11*, *RAD6*, and *SGS1).* The *CDC55* gene was described as involved in mitotic cell cycle, only, but the corresponding deletion mutant exhibited a strong susceptibility phenotype. 

#### 3.2.2. Genes Involved in Autophagy

Sixteen genes involved in autophagy were found to be implicated in yeast tolerance to methanol (Table 2). Autophagy is a highly conserved eukaryotic cellular recycling process playing an important role in cell survival and maintenance, involving the formation of the autophagosome [34]. The individual deletion of the macroautophagy genes, *AIM26*, *ATG11*, *RAS2*, *VAM7*, *VPS36*, and *YPT6* (Table 2) led to a strong methanol susceptibility phenotype. Collectively, these results suggest that methanol may have strong deleterious effects on intracellular proteins and organelles, targeting cytoplasmic contents and organelles into autophagosomes for degradation as part of the protective response.

Genes involved in reticulophagy (*ATG11*, *SNF7*, *SPO7*, *STP22*, *VPS21*, *VPS4*, Table 2), a type of selective autophagy required for the selective clearance and degradation of the endoplasmic reticulum (ER) by the cellular macroautophagy/autophagy machinery under endoplasmic reticulum stress [34], were also found to be enriched in both methanol and ethanol datasets. The ER is the main site for cellular protein and calcium homeostasis, as well as lipid synthesis in eukaryotic cells [35].

#### 3.2.3. Genes Involved in Reserve Polysaccharides, Cell Wall and Membrane Biosynthesis

Genes implicated in cell wall and membrane biosynthesis are also relevant in yeast tolerance to methanol through their function in polysaccharide metabolism and fatty acid biosynthesis (Table 3). Regarding polysaccharide metabolism, the deletion of *FKS1*, *GPH1 ROT2*, *SMI1*, and *TPS2* genes led to strong susceptibility phenotypes. Remarkably, genes involved in the catabolism and synthesis of the reserve carbohydrates glycogen and trehalose (*GPH1* and *TPS2* genes) and cell wall synthesis (*FKS1*, *ROT2*, and *SMI1*) are shared by the two datasets being required for maximum tolerance to both alcohols.

Genes involved in membrane synthesis relevant for methanol and ethanol tolerance, include ergosterol biosynthetic genes (*ERG2* and *ERG3*), phospholipid biosynthetic genes (*KCS1*, *LIP5*, *PDX3*), and sphingolipids biosynthetic genes (*ELO2*, *ELO3*, and *SAC1*). In particular, the deletion of the *ELO2* gene, encoding the fatty acid elongase involved in fatty acids and sphingolipids biosynthesis, led to a strong phenotype.

#### 3.2.4. Genes Involved in Protein Synthesis

Methanol tolerance determinants involved in protein synthesis, through cytoplasmic and mitochondrial translation, include a large number of genes encoding (i) proteins of the large subunits of ribosomes—Ribosomal Proteins of the Large subunit, *RPL* genes (e.g., *RPL8A*, *RPL2B*, *RPL34B*, *RPL14A*, *RPL31B*, *RPL20A*, *RPL36B*); (ii) proteins of the small subunit of ribosomes, Ribosomal Proteins of the Small subunit, *RPS* genes (e.g., *RPS17A*, *RPS19A*) (Supplementary Material, Table S1). 

The mitochondrial large subunit proteins encoded, mostly, by the family of the Mitochondrial Ribosomal Proteins of the Large subunit, *MRPL* genes (e.g., *MRP49*, *MRPL25*, *MRPL8*, *MRPL33*, *MRPL10*, *MRPL33*), and the mitochondrial small subunit proteins, encoded by the Mitochondrial Ribosomal Small subunit of Mitochondria (*MRPS)* and Ribosomal Small subunit of Mitochondria *(RSM)* families of genes (e.g., *MRP13*, *MRPS35*,, *RSM18*, *RSM22*, *RSM23*) were required to overcome methanol-induced stress (Supplementary Material, Table S1).

Several other genes related with cytoplasmic translation, (*RPS16A*, *RPL20B*, *RPL21A*, *RPS24A*, *RPS27B*) and mitochondrial translation, (*GTF1*, *IFM1*, *MRF1*, *MRP7*, *MRPS12*, *MRPL20*, *MRPL22*, *MRPL24*, *MRPL7*, *MSE1*, *RSM23*, *SLM5*) were found to confer tolerance, to both methanol and ethanol (Supplementary Material, Tables S1 and S2).

#### 3.2.5. Genes Involved in Vacuolar Function and Endosomal Transport

ATP export and intralumenal vesicle formation are the two functions with the highest level of fold enrichment in both methanol and ethanol datasets sharing 12 genes (*BRO1*, *DID4*, *DOA4*, *SNF7*, *SNF8*, *STP22*, *VPS20*, *VPS24*, *VPS25*, *VPS27*, *VPS28*, *VPS36*) with vacuolar and endosomal functions (Figure 3 and Table 4 and Table 5).

Results suggest that yeast cells exposure to methanol stress affects several transport routes such as vacuolar transport and vesicular transport. Methanol tolerance determinants related with vacuolar function include Vacuolar Membrane H^+^- ATPase (*VMA*) genes (involved in intracellular pH homeostasis [36,37], the vacuolar morphogenesis (*VAM)* genes *VMA1*, *VMA2*, *VMA7*, *VMA13*, *VAM7* (leading to a strong phenotype) and *VAM3*, *VAM6*, *VAM7*, *VAM10* (leading to a moderate phenotype) and the vacuolar protein sorting (*VPS*) genes—*VPS1*, *VPS24*, *VPS25*, *VPS33*, *VPS36* (Table 4).

Genes involved in vesicular transport in the datasets include those encoding the endosomal sorting complex (*BRO1*, *DID2*, *DID4*, *SNF7*, and *SNF8*) (Table 5). 

#### 3.2.6. Transcriptional Control and Regulatory Tolerance Networks

The enriched biological functions “chromatin remodeling” and “silencing” in the methanol dataset contains, among other genes, 12 transcription factors (TF) (Table 6). Five of these genes are shared with the ethanol dataset (Table 6).

**Figure 4:**
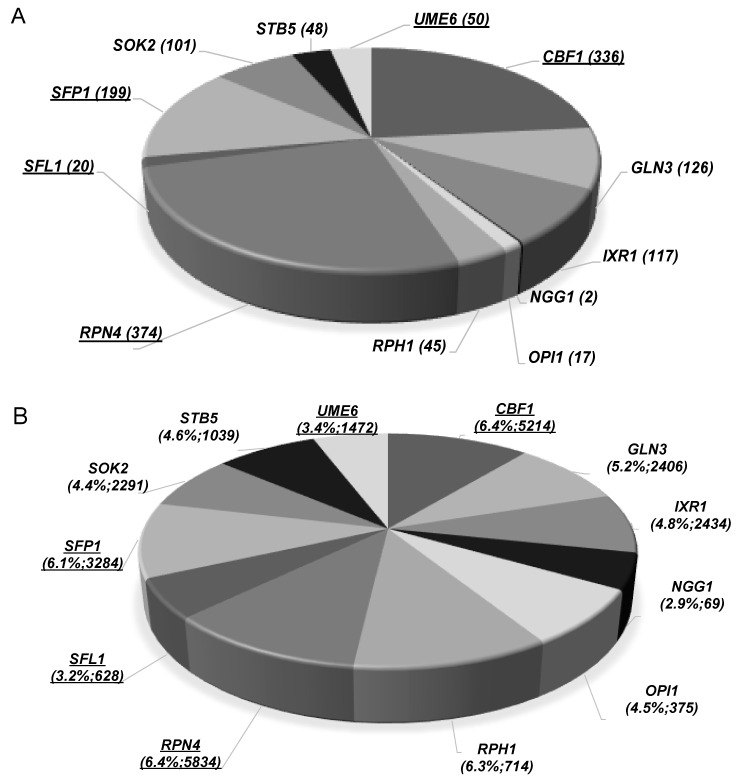
Clustering of methanol-tolerance genes that are targets of transcription factors (TF) that also exert protection against this alcohol. (**A**) Number of target genes (into brackets) for each TF that are required for methanol tolerance. (**B**) Percentage of methanol tolerance genes regulated by each TF relatively to the total number of genes of each described regulon. Methanol tolerance genes were clustered in association with their documented regulators using the information available in the Yeastract database (December 2020). The TFs found to exert protection only against methanol are in bold; the TFs underlined and in bold are those found in the methanol and ethanol datasets.

Using the Yeastract database [38], it was found that the number of genes in the methanol dataset described as being regulated by the identified TFs is variable (numbers indicated in Figure 4A), ranging from 2 *(NGG1*) to 374 (*RPN4*). However, the described regulons corresponding to those TFs also include a highly variable number of target genes (from 69–5834 genes, Figure 4B). For this reason, the percentage of methanol tolerance genes among the various regulons was calculated and the methanol tolerance genes were found to represent 3–6.4% of the described TF target genes, reaching values above 6% for the TFs Cbf1, Sfp1, Rpn4, and Rph1. Based on the levels of the methanol susceptibility phenotypes of the corresponding deletion mutants estimated in this study and the percentage of genes of the described regulons identified as methanol determinants, the TFs Cbf1, Sfp1, Rpn4, Ixr1, Opi1, Sfl1, Sok2, Stb5, and Ume6 are suggested as having an important and more specific role in methanol tolerance in *S. cerevisiae.* The TFs Cbf1, Sfl1, Sfp1, Rpn4, and Ume6, are also considered relevant determinants of ethanol tolerance.

The methanol susceptibility phenotypes observed during this high throughput analysis were confirmed by growth in shake flasks (Supplementary Material, Figure S2). 

**Figure 5:**
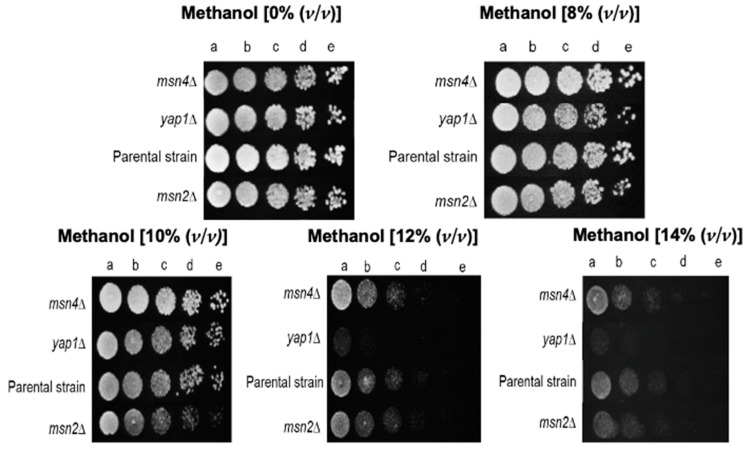
Susceptibility to methanol of the parental strain *S. cerevisiae* BY4741 and derived deletion mutants *yap1*∆, *msn4*∆, and *msn2*∆ by spot assays. Yeast cell suspensions used as inocula for spot assays were prepared using cells harvested in the exponential phase of growth (culture OD_600nm_ = 0.5 ± 0.05). Cell suspensions were diluted in sterile water to an OD_600nm_ = 0.25 ± 0.005 (a) and this solution was used to prepare 1:5 (b), 1:25 (c), 1:125 (d), and 1:625 (e) diluted suspensions. Susceptibility phenotypes were registered after 48 h of incubation at 35 °C.

Oxidative stress is being considered a major consequence of methanol-induced stress in *S. cerevisiae* [14,39]. Therefore, it was intriguing the absence of the well-known transcriptional regulators (TR) of the general stress response, Msn2 and Msn4, and of the oxidative stress response, Yap1 [40,41] in the obtained dataset. The hypothesis that the concentration of methanol used for screening the disruptome was below the threshold level for rendering those TR active was tested. When YPD medium was supplemented with 8% (*v*/*v*) methanol (the conditions used for the disruptome screening), the lack of phenotype for *msn2*∆, *msn4*∆ and *yap1*∆ mutants was confirmed (Figure 5). However, at methanol concentrations in the range 10–14% (*v*/*v*), the *yap1*∆ exhibited a marked methanol susceptibility phenotype confirming the importance of Yap1 for methanol tolerance. Nevertheless, the growth inhibition of the single *msn2* and *msn4* deletion mutants by methanol, compared with the parental strain, was minor, if any. This result is consistent with former evidences indicating that the pleiotropic stress sensitivity phenotype of the single deletion mutants *msn2*∆ and *msn4*∆ is milder than expected, considering the number of STRE-containing genes, being only observed under severe stress conditions [41]. A possible explanation for this behavior is that STRE are redundant with other regulatory systems for genes with essential roles in the stress response. Moreover, genetic evidence suggests that Msn2p and Msn4p are functionally redundant and that only the phenotype of the double deletion mutant is evident [41].

**Figure 6:**
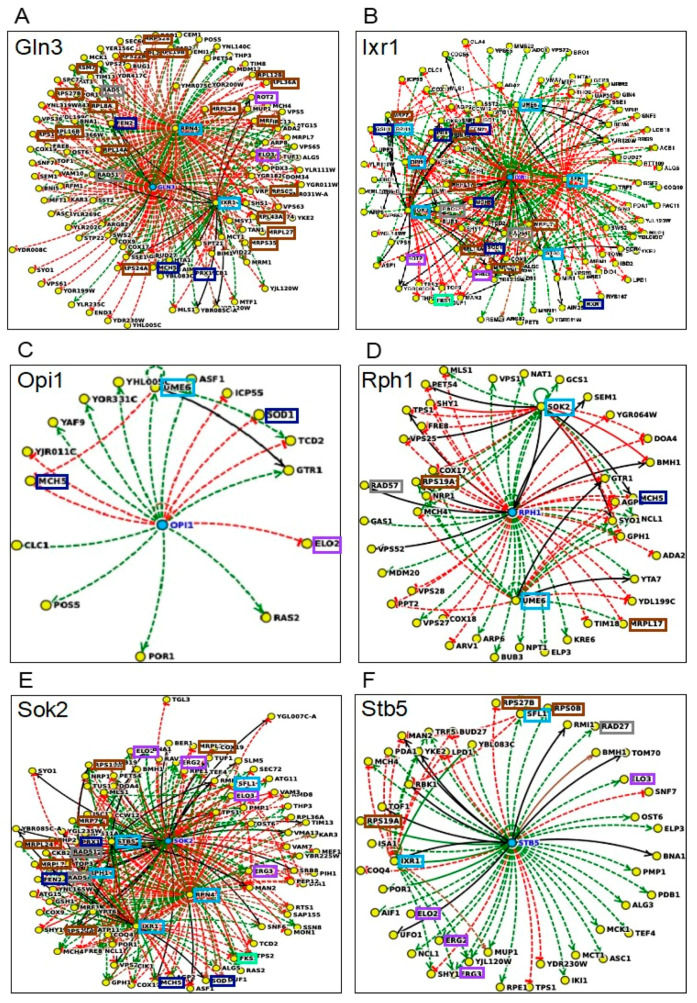

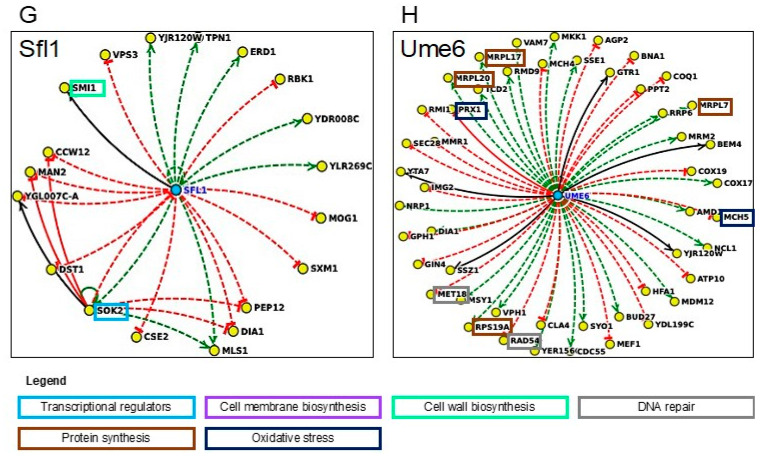
Genetic interaction networks between the TFs Gln3 (**A**), Ixr1 (**B**), Opi1 (**C**), Rph1 (**D**), Sok2 (**E**), Stb5 (**F**), Sfl1 (**G**) and Ume6 (**H**), required for tolerance to methanol and the methanol tolerance genes identified in this study that are their documented targets. The interaction networks were obtained using the regulatory interactions available in Yeastract (December 2020, http://yeastract-plus.org/yeastract/scerevisiae/index.php). The positive, negative, and unspecified regulatory interactions, correspond, to green, red, and black lines, respectively. The interactions are based in expression data (dashed lines) or DNA binding (full lines). The color of the boxes represents different functional groups as indicated in the legend.

Based on the documented interactions (DNA binding evidences or DNA expression data) deposited in the Yeastract database [38], the complex regulatory networks describing the interactions between the main TFs found to be specifically required for methanol tolerance (Gln3, Ixr1, Opi1, Rph1, Sok2, Stb5) and their target genes in the methanol dataset is shown in Figure 6A–F, respectively. These methanol-specific TFs control a considerable number of the genes with strong phenotype that participate in methanol enriched biological functions discussed previously, such as DNA repair (e.g., *MET18*, *RAD27*, *RAD5*, *RAD51*, *RAD54*, *RAD57*), cell wall biosynthesis (e.g., *FKS1* and *SMI1*), membrane biosynthesis (e.g., *ELO2*, *ELO3*, *ERG2*, *ERG3*, *ROT2*), oxidative-stress responsive genes (e.g., *FEN2*, *GSH1*, *MCH5*, *PRX1*, *SOD1*) and protein synthesis (e.g., *MRPL*, *MRPS*, *RPL*, and *RPS)* families of genes. The transcriptional network controlled by the TF Ixr1, that regulates hypoxic genes during normoxia, is highly complex since it controls, the highest number of genes (6) encoding other transcription factors of the methanol dataset: Gln3, Opi1, Rph1, Sok2, Stb5, and Ume6 [42,43,44,45]. Ngg1 was not included in this analysis due to the low number of regulatory interactions available at Yeastract database. The regulatory interaction networks for the TFs that are shared between methanol and ethanol datasets, Sfl1 and Ume6, with the genes that confer methanol tolerance are presented in Figure 6, panels G and H, respectively. The regulatory networks for Cbf1, Rpn4, and Sfp1 are not shown because, due to the high density of the regulated methanol tolerance genes, they do not provide useful information.

## 4. Discussion

The chemogenomics analysis performed in this work provides new information on methanol toxicity and tolerance mechanisms in yeast, being the first disruptome study conducted to unveil methanol tolerance determinants at a genome-wide scale. Although the identification of methanol tolerance determinants in yeast was the main objective of this study, we also intended to compare the major determinants of tolerance to methanol versus ethanol to get clues on more specific methanol toxicity mechanisms and to identify relevant tolerance determinants common to both alcohols to guide future efforts concerning yeast robustness engineering. The yeast disruptome was previously screened for susceptibility to ethanol stress in several studies [20,21,22,23,24,25]. However, differences registered between the data obtained in the various experimental analyses suggest that the genetic background, growth media, level of stress, and other environmental conditions influence the effect of gene expression in yeast tolerance [22]. To address this issue, two datasets were obtained in this work by testing equivalent sub-lethal inhibitory concentrations of methanol and ethanol under the same experimental conditions. Among the 402 determinants of tolerance to methanol-induced stress, identified in YPD growth medium supplemented with 8% (*v*/*v*) at 35 °C, 235 were specific to methanol, thus not shared with the ethanol dataset obtained for 5.5% (*v*/*v*) ethanol. The main feature that clearly distinguishes methanol from ethanol tolerance determinants relates with DNA repair mechanisms presumably required for overcoming methanol-induced stress. Among the eight *RAD* genes (a designation due to the sensitivity of the corresponding mutants to exposure to X-rays [46]) exclusively present in the methanol dataset and involved in several mechanisms of DNA repair, such as recombinational repair and double-strand break repair [47,48] are genes, previously identified as formaldehyde tolerance determinants: the *RAD18*, *RAD27*, *RAD5*, *RAD51*, and *RAD57* [16]. The mechanisms proposed to be required for formaldehyde tolerance involve homologous recombination and nucleotide excision repair; while homologous recombination is considered the preferred mechanism to repair damage due to chronic exposure to formaldehyde, nucleotide excision repair is the preferred mechanism to repair acute exposure [17]. The identification of genes involved in homologous recombination (Rad51, Rad54, Rad57) and in nucleotide excision repair (Rad33) indicates that both mechanisms are important for methanol tolerance.

Although there are differences between the major methanol or ethanol tolerance determinants, there are tolerance mechanisms in common. Since straight-chain alcohols toxicity can be related with the octanol-water partition coefficient log P_ow_ value [49], and log P_ow_ values for methanol and ethanol are −0.74 and −0.30, respectively [50], compared with methanol, ethanol is more lipophilic and, therefore, expectably, more toxic as the result of membrane targeting [51,52]. Consistent with the concept that membranes are molecular targets for methanol and ethanol toxic effects, genes required for membrane composition control were identified as required for alcohol tolerance, in particular genes involved in the biosynthesis of ergosterol, phospholipids, and sphingolipids [20,21,22,23,24,25]. In this context, the TF Ume6, an important ethanol tolerance determinant [20,21,22,24] that is known to regulate phospholipid biosynthetic gene expression [53], was found in methanol and ethanol datasets. Together with other tolerance genes involved in the establishment of membrane composition, those genes reinforce the idea of the importance of membrane remodeling to counteract alcohol stress. Alcohol-induced permeabilization of plasma membrane leads to the increased passive influx of ions, in particular protons, across plasma membrane, contributing to cytosolic acidification and to the dissipation of the electrochemical membrane potential [36,37,54]. Since the H^+^-ATPase, present at the vacuolar membrane (V-ATPase), is involved in the regulation of intracellular pH homeostasis [36] it is understandable that several subunits of this multimeric protein were identified as required for methanol and ethanol tolerance. Methanol is likely an inducer of intracellular and vacuolar acidification, as described for ethanol [22,55,56] and the maintenance of intracellular pH (pH_i_) homeostasis is affected by V-ATPase defects, emphasizing the importance of this cellular function in tolerance to various straight-chain alcohols [21]. The biosynthesis of the reserve polysaccharides, glycogen and trehalose also appears to be relevant for yeast tolerance to methanol and ethanol, as suggested by the identification of two genes involved in the synthesis of trehalose (*TPS1* and *TPS2)* and one gene required for glycogen degradation (*GPH1)* in the datasets. Trehalose and glycogen accumulate under stress conditions and are important in carbon storage and as compatible solutes [57] and trehalose exerts a protective effect on biomembranes, avoiding desiccation and protein denaturation [58]. *TPS1* and *GPH1* were also found in other chemogenomic studies as ethanol tolerance determinants [22,23,24,25]. 

The importance of autophagic processes in methanol and ethanol tolerance is also suggested by our study, a role that can be related with cell protection against DNA and other macromolecules and organelle damaging [34,59]. In fact, autophagy is considered a central component of the global stress response [59]. Reticulophagy, a type of selective autophagy [34], is an enriched biological function in both datasets. These results are consistent with previous studies reporting that ethanol exposure can lead to endoplasmic reticulum stress, contributing to impaired protein folding and inducing the unfolded protein response [60]. Methanol also decreases the level of hydration of proteins, leading to tertiary structure modifications, in which polar groups are exposed and can interact mutually [39,61]. Formaldehyde, due to reaction with the amino- and sulfhydryl- groups in small molecules, peptides, proteins and nucleic acids, contributes to the formation of inter- and intramolecular bridges [39,62]. These conformational changes, caused by methanol or formaldehyde, are comparable to the effect of ethanol inducing endoplasmic reticulum stress, consistent with a common cellular response to these alcohols. The de novo synthesized proteins from the endoplasmic reticulum, destined for secretion from the cell, endocytic processes or to the plasma membrane are delivered to the Golgi apparatus [63]. The retention of proteins in the Golgi apparatus, suggested by specific methanol and ethanol tolerance determinants obtained in the two datasets of this study, in particular *DID4*, *VPS1*, *VPS27*, *VPS36*, *VPS4*, and *VPS5* genes, corroborates the occurrence of changes in protein structure induced by the alcohols linking protein defects due to alcohol exposure to protein sequestration in the Golgi. Furthermore, the processes of intracellular trafficking, including vacuolar protein targeting, endosome transport, and transport mediated by the endosomal sorting complexes needed for transport (ESCRT-I, -II, and -III), as well as ubiquitin-dependent protein sorting to the vacuole, were found to confer methanol tolerance, similarly to what was described for ethanol [20,21,22,23,24]. The endosomal sorting complexes, as well as ubiquitin-dependent sorting to the vacuole, are important for degradation of methanol/formaldehyde- and ethanol-induced misfolded proteins that were likely to suffer damage due to alcohol toxicity.

Concerning the putative regulatory networks involved in methanol tolerance, twelve TFs were identified in our chemogenomic analysis. Five of them are relevant to overcome the stress induced by either methanol or ethanol: Cbf1, Rpn4, Sfl1, Sfp1, and Ume6. Regarding Rpn4 and Sfp1, these TFs reportedly confer tolerance to a wide variety of environmental stresses [64,65,66]. The regulation data available in Yeastract pointed out Rpn4 as a major regulator: 5834 genes of yeast genome are under Rpn4 control and above 90% of the alcohol tolerance genes present in both datasets are known to be under Rpn4 regulation. This TF was previously reported to be activated by ethanol shock [67] or short exposure to this alcohol [68]. Rpn4 stimulates the expression of proteasome subunit genes as well as of genes involved DNA repair [66], which are enriched biological functions in the methanol dataset. The regulation of the DNA-damage response is also dependent on Sfp1 [69], which was previously identified as an ethanol tolerance determinant [22]. Cbf1 regulates around 80% of methanol and ethanol datasets, being the second major regulator of alcohol tolerance genes. The Yeastract tool “Rank by TF”, enabling automatic selection and ranking of transcription factors potentially involved in the regulation of the genes of methanol and ethanol datasets, attributed a significant p-value to Cbf1 and Rpn4 (<0.05 in both datasets) and for Sfp1 (<0.06 and 0.08, in methanol and ethanol datasets, respectively). These results emphasize the importance of these TFs in the regulation of methanol and ethanol tolerance genes and its potential as candidates for alcohol tolerance engineering. Although the genetic engineering of TFs by modulating their activity can lead to an imbalance in metabolic reactions, this strategy has been proposed as an important approach for improvement of yeast tolerance to several toxicants. Successful approaches using this strategy includes Haa1 engineering to increase *S cerevisiae* acetic acid tolerance [70] or the tolerance to a mixture of acetic acid and furfural by the increased co-expression of Haa1 and Tye7 [71] or Ace2 and Sfp1 [65]. To improve methanol and ethanol tolerance, Cbf1, Rpn4 and Sfp1 are promising molecular targets of genetic engineering. Recently, the heterologous expression of IrrE, a global regulatory protein from the prokaryotic organism *Deinococcus radiodurans*, was engineered to improve yeast tolerance to inhibitors present in lignocellulose hydrolysates or to high temperatures [72]. 

Concerning the TFs encoded by genes only found in the methanol dataset, *GLN3*, *IXR1*, *NGG1*, *OPI1*, *RPH1*, *SOK2*, and *STB5*, they are involved in several bioprocesses, such as nitrogen catabolism [73], oxygen sensing [45], histone acetylation and demethylation [74,75], lipid biosynthesis [76], autophagy [77], pseudohyphal growth [78], and oxidative stress response [79].

Ixr1 and Opi1 are suggested to play a significant role in methanol tolerance since the corresponding deletion mutants exhibited a marked methanol susceptibility phenotype. Ixr1 is a transcriptional repressor of hypoxic genes’ transcription during normoxia [45] and in the corresponding deletion mutant, *ixr1*Δ, the expression of genes related to ribosomal genes are downregulated [44]. Ixr1 also controls the levels of dNTPs in the cell required for DNA synthesis and repair [80]. Additionally, *IXR1* may be involved in the oxidative stress response, since the mutant *ixr1*Δ is more susceptible to peroxides [81] and its promoter region has a binding site for *STB5*, a regulator of the oxidative stress response [44]. Opi1 is a transcriptional repressor involved in the regulation of phospholipid synthesis in response to inositol availability [76]. The deletion of the *OPI1* gene was found to jeopardize mitochondrial metabolism, by decreasing the levels of cardiolipin by 50%, resulting in low cytochrome content and high mitochondrial DNA instability [82]. Our results suggest, for the first time, the importance of mitochondrial genome in methanol tolerance, similarly to what has been proposed for ethanol [22,83]. Given that *IXR1* is involved in the regulation of around 30% of the genes in the methanol dataset, including other transcription factors encoding genes (*OPI1*, *RPH1*, *SFP1*, *SOK2*, *STB5*, and *UME6*) [42,43,44,45] and the deletion mutant exhibits a strong methanol susceptibility phenotype, this regulator, together with Opi1 can be, in principle, considered promising candidate molecular targets to be tested for TF engineering in *S. cerevisiae*.

Methanol oxidation is accompanied by the production of free radicals in complex eukaryotes [39] and, in yeast, oxidative stress was pointed out as a major consequence of exposure to methanol [14]. Formic acid is also an oxidative stress inducer and, in mammalians, formate binds to cytochrome c, inhibiting the last step of the electron transport chain in mitochondria [84] and to the disruption of the proton gradient and the consequent decrease of ATP synthesis [85]. In yeast, formic acid can lead to the rapid burst of intracellular reactive oxygen species [86] and, consequently, to oxidative stress [87]. In addition to *IXR1* and *STB5*, other oxidative stress responsive genes were found to be determinants of methanol tolerance, including genes encoding antioxidant enzymes such as superoxide dismutase (Sod1), methionine-S-sulfoxide reductase (Mrx2), peroxiredoxin (Prx1), as well as Gsh1, a gamma glutamylcysteine synthetase involved in the first step of glutathione biosynthesis. Additionally, two transporters, Fen2 and Mch5 involved in overcoming oxidative stress [88,89] are relevant due to the inability of the corresponding deletion mutants to grow in methanol. Fen2 is a H^+^-pantothenate (vitamin B5) symporter [90] and mutations in *FEN2* lead to reduced biosynthesis of ergosterol and fatty acids [90], which emphasizes the importance of membrane composition and properties in methanol tolerance. In addition, the *Corynebacterium glutamicum* Cgl0833, a Na+/panthothenate symporter, is required for methanol tolerance [91]. Mch5 is a transporter from the major facilitator superfamily that proceeds to the uptake of riboflavin [92], a vitamin that is required for the activity of glutathione reductase in the FAD coenzyme form [88]. Although many important TFs for methanol tolerance were identified in this genome-wide study, the major regulator of the response to oxidative stress, Yap1 were not identified but, for higher levels of methanol stress, *YAP1* expression was confirmed as a critical methanol tolerance determinant. 

**Figure 7:**
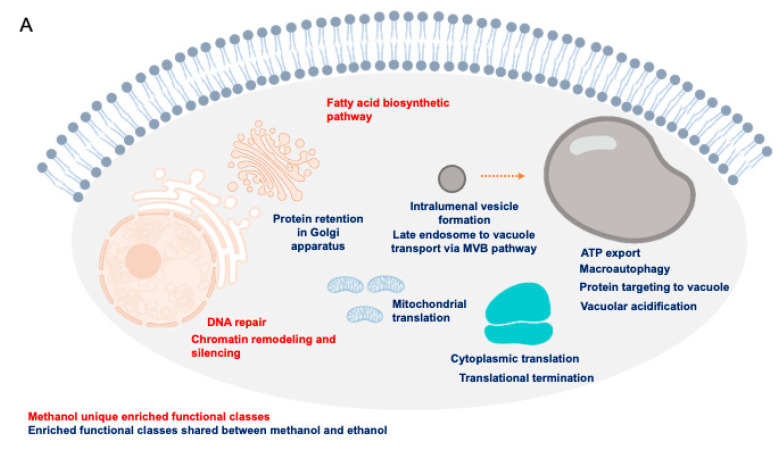

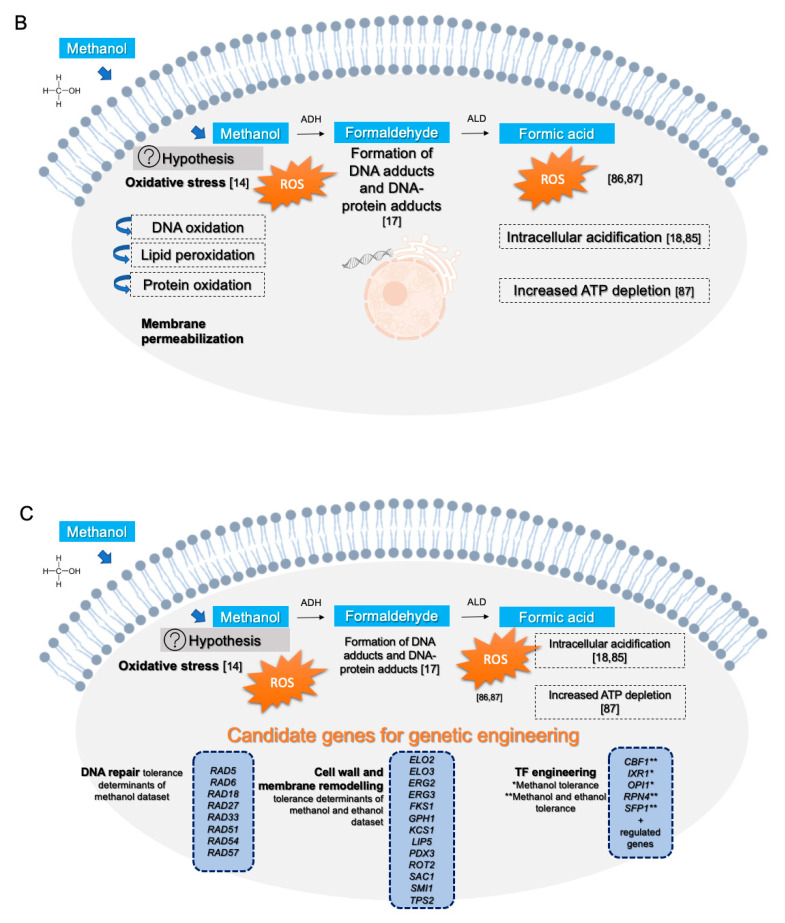
Schematic model proposed, for methanol toxicity, including the detoxification intermediates formaldehyde and formic acid. (**A**) Biological functions found to be enriched in the methanol dataset. The biological functions shared with the ethanol dataset are in blue and the enriched biological functions, specific to the methanol dataset, are in red. (**B**) Methanol detoxification pathway and the anticipated toxic effects of each metabolic intermediate, in particular having DNA as an important molecular target for the formic acid-induced ROS [86] and formaldehyde alkylating activity. (**C**) To overcome the effects of methanol in yeast cells, DNA repair mechanisms play an important role in preserving DNA integrity after methanol exposure; mechanisms found to be relevant for methanol tolerance involve, among others, the *RAD* genes, and other genes involved in DNA repair and in the oxidative stress response. Several TFs found to confer methanol tolerance are additional potential targets for genetic engineering to obtain more robust strains, able to cope with deleterious concentrations of methanol.

Despite the potential of methanol as a (co)substrate for the biotechnology industry, the global mechanisms by which this alcohol exerts its toxicity are still unclear. Based on the results from this study and on the above referred discussion, a schematic model on the hypothesized methanol toxicity and tolerance mechanisms are presented, specifying the more specific mechanisms and those shared with ethanol (Figure 7A). The reported toxicity mechanisms of the detoxification intermediates formaldehyde and formic acid are also included (Figure 7B). 

Results from this genome-wide search for methanol tolerance genes can also be explored for the rational genomic manipulation of the yeast cell to obtain more robust strains capable of coping with high methanol or high methanol and ethanol concentrations by exploring information on a cumulative inhibitor tolerance phenotype, as recently reported for *S cerevisiae* engineering towards improved tolerance to for the multiple inhibitors present in lignocellulosics-based fermentations [93]. Since TFs engineering is an emerging, although somewhat controversial, strategy to increase yeast tolerance to different biotechnological relevant stresses [65,70,71], it would be interesting to explore the modulation of the expression or the alteration of the amino acid sequence of the TFs here identified as promising (Figure 7C). Among them are Cbf1, Rpn4 and Sfp1, for alcohol tolerance, and Ixr1 and Opi1, specifically for methanol tolerance. The knowledge here obtained, and the list of genes provided in this study can be considered an important starting point for the improvement of yeast tolerance to methanol or to methanol and ethanol in biotechnologically relevant yeast species for which the necessary genome sequence and editing tools are currently available or could be developed. The exploitation of the bioinformatics tool NCYeastract database (Non- Conventional Yeastract; http://yeastract-plus.org/ncyeastract/) [38] will facilitate the identification of orthologous genes in the yeast species currently included in the database as well as the regulatory associations already described for other yeast species, especially for *S. cerevisiae* in the sister database Yeastract (http://yeastract-plus.org/yeastract/scerevisiae/index.php), using the new tools for cross-species transcription regulation comparison [38]. In particular, this is currently a useful resource to guide the genetic engineering of biotechnologically relevant yeasts, such as the methylotrophic yeast species *Komagataella phaffii* and the oleaginous yeast species *Yarrowia lipolytica* [38].

## Figures and Tables

**Table 1:** Genes involved in DNA repair and mitotic cell cycle identified in this study as determinants of yeast tolerance to methanol, compared with ethanol. The description of the encoded protein functions is based on the information at SGD (www.yeastgenome.org). The classification of the susceptibility phenotype level is as described in the Section 2.

Gene/ORF	Description of the Encoded Protein Function	Susceptibility to Methanol	Susceptibility to Ethanol
*CDC55*	Regulatory subunit B of protein phosphatase 2A (PP2A), which localizes to nucleus prevents mitotic exit.	+++	No phenotype
*MET18*	Component of cytosolic iron-sulfur protein assembly (CIA) machinery. Met18 acts at a late step of Fe-S cluster assembly and it is also involved in DNA replication and repair, transcription, and telomere maintenance.	+++	+
*MRE11*	Nuclease subunit of the MRX complex with Rad50and Xrs2; MRX complex functions in repair of DNA double-strand breaks and in telomere stability.	+++	No phenotype
*RAD5*	DNA helicase/Ubiquitin ligase; involved in error-free DNA damage tolerance (DDT), replication fork regression during post-replication repair by template switching, error-prone translesion synthesis.	++	No phenotype
*RAD6*	Ubiquitin-conjugating enzyme (E2); involved in post-replication repair as a heterodimer with Rad18, regulation of K63 polyubiquitination in response to oxidative stress, double-strand break repair and checkpoint control and as a heterodimer with Bre1.	+	No phenotype
*RAD18*	E3 ubiquitin ligase; required for post-replication repair.	++	No phenotype
*RAD27*	5′ to 3′ exonuclease, 5′ flap endonuclease; required for Okazaki fragment processing and maturation, for long-patch base-excision repair.	+++	No phenotype
*RAD33*	Protein involved in nucleotide excision repair.	+	No phenotype
*RAD51*	Strand exchange protein involved in the recombinational repair of double-strand breaks in DNA during vegetative growth and meiosis.	+++	No phenotype
*RAD57*	Protein that stimulates strand exchange by stabilizing the binding of Rad51 to single-stranded DNA; involved in the recombinational repair of double-strand breaks in DNA during vegetative growth and meiosis.	++	No phenotype
*SGS1*	RecQ family nucleolar DNA helicase. Sgs1 play a role in genome integrity maintenance, chromosome synapsis, meiotic joint molecule/crossover formation.	++	++

**Table 2:** Genes involved in autophagy identified in this study as determinants of yeast tolerance to methanol, compared with ethanol. Genes in bold are specific to macroautophagy and the corresponding mutants exhibited a strong susceptibility phenotype, and the underlined genes are specific to reticulophagy. Atg11 is common to both types of autophagy. Symbols are as in Table 1.

Gene/ORF	Description of the Encoded Protein Function	Susceptibility to Methanol	Susceptibility to Ethanol
*AIM26*	Protein of unknown function. Null mutant displays elevated frequency of mitochondrial genome loss.	+++	No phenotype
***ATG11***	Adapter protein for pexophagy and the Cvt targeting pathway. Atg11 directs receptor-bound cargo to the phagophore assembly site (PAS) for packaging into vesicles.	+++	++
***RAS2***	GTP-binding protein that regulates nitrogen starvation response, sporulation, and filamentous growth.	+++	+++
*SNF7*	One of four subunits of the ESCRT-III complex. Snf1 is involved in the sorting of transmembrane proteins into the multivesicular body (MVB) pathway.	+++	+++
*STP22*	Component of the ESCRT-I complex.	++	+++
***VAM7***	Vacuolar SNARE protein.	+++	+
*VPS21*	Endosomal Rab family GTPase required for endocytic transport and sorting of vacuolar hydrolases. Vps21 is also required for endosomal localization of the CORVET complex.	++	++
*VPS4*	AAA-ATPase involved in multivesicular body (MVB) protein sorting.	++	+
***VPS36***	Component of the ESCRT-II complex that contains the GLUE (GRAM Like Ubiquitin binding in EAP45) domain which is involved in interactions with ESCRT-I and ubiquitin-dependent sorting of proteins into the endosome.	+++	+++
***YPT6***	Rab family GTPase that is required for endosome-to-Golgi, intra-Golgi retrograde, and retrograde Golgi-to-ER transport.	+++	+++

**Table 3:** Genes involved in reserve carbohydrate, cell wall, and membrane biosynthesis identified in this study as determinants of yeast tolerance to methanol, compared with ethanol. Symbols are as in Table 1.

Gene/ORF	Description of the Encoded Protein Function	Susceptibility to Methanol	Susceptibility to Ethanol
*ELO2*	Fatty acid elongase, involved in sphingolipid biosynthesis; acts on fatty acids of up to 24 carbons in length.	+++	+++
*ELO3*	Elongase involved in fatty acid and sphingolipid biosynthesis.	++	++
*ERG2*	C-8 sterol isomerase; catalyses isomerization of delta-8 double bond to delta-7 position at an intermediate step in ergosterol biosynthesis.	+	+++
*ERG3*	C-5 sterol desaturase; glycoprotein that catalyses the introduction of a C-5(6) double bond into episterol, a precursor in ergosterol biosynthesis.	++	++
*FKS1*	Catalytic subunit of 1,3-beta-D-glucan synthase; binds to regulatory subunit Rho1; involved in cell wall synthesis and maintenance.	+++	+++
*GPH1*	Glycogen phosphorylase required for the mobilization of glycogen.	+++	++
*KCS1*	Inositol hexakisphosphate and inositol heptakisphosphate kinase.	++	+++
*LIP5*	Protein involved in biosynthesis of the coenzyme lipoic acid.	+	+
*PDX3*	Pyridoxine (pyridoxamine) phosphate oxidase.	++	+++
*ROT2*	Glucosidase II catalytic subunit; required to trim the final glucose in N-linked glycans and for normal cell wall synthesis.	+++	++
*SAC1*	Phosphatidylinositol phosphate (PtdInsP) phosphatase; is involved in hydrolysis of PtdIns(4)P in the early and medial Golgi.	+	++
*SMI1*	Protein involved in the regulation of cell wall synthesis.	+++	++
*TPS2*	Phosphatase subunit of the trehalose-6-P synthase/phosphatase complex that involved in synthesis of the storage carbohydrate trehalose.	+++	+

**Table 4:** Genes involved in vacuolar organization and acidification identified in this study as determinants of yeast tolerance to methanol, compared with ethanol. Symbols are as in Table 1.

Gene/ORF	Description of the Encoded Protein Function	Susceptibility to Methanol	Susceptibility to Ethanol
*VAM10*	Protein involved in vacuole morphogenesis and acts at an early step of homotypic vacuole fusion that is required for vacuole tethering.	+	++
*VAM3*	Syntaxin-like vacuolar t-SNARE. Vam3 mediates docking/fusion of late transport intermediates with the vacuole.	+	++
*VAM6*	Guanine nucleotide exchange factor for the GTPase Gtr1. Vam6 is a Rab GTPase effector, interacting with both GTP- and GDP-bound conformations of Ypt7.	+	++
*VAM7*	Vacuolar SNARE protein; Vam7 functions with Vam3 in vacuolar protein trafficking.	+++	+
*VMA1*	Subunit A of the V1 peripheral membrane domain of V-ATPase.	+++	+++
*VMA13*	Subunit H of the V1 peripheral membrane domain of V-ATPase.	+++	+++
*VMA2*	Subunit B of V1 peripheral membrane domain of vacuolar H^+^-ATPase.	+++	No phenotype
*VMA7*	Subunit F of the V1 peripheral membrane domain of V-ATPase.	+++	No phenotype
*VPS1*	Dynamin-like GTPase required for vacuolar sorting.	+++	+++
*VPS24*	One of four subunits of the ESCRT-III complex. Vps24 is involved in the sorting of transmembrane proteins into the multivesicular body (MVB) pathway.	+++	+++
*VPS25*	Component of the ESCRT-II complex.	+++	++
*VPS33*	ATP-binding protein that is a subunit of the HOPS and CORVET complexes. Vps33 is essential for protein sorting, vesicle docking, and fusion at the vacuole.	+++	+++
*VPS36*	Component of the ESCRT-II complex.	+++	+++

**Table 5:** Genes involved in vesicular transport identified in this study as determinants of yeast tolerance to methanol, compared with ethanol. Symbols are as in Table 1.

Gene/ORF	Description of the Encoded Protein Function	Susceptibility to Methanol	Susceptibility to Ethanol
*BRO1*	Cytoplasmic class E vacuolar protein sorting (VPS) factor. Bro1 coordinates deubiquitination in the multivesicular body (MVB) pathway by recruiting Doa4 to endosomes.	++	No phenotype
*DID2*	Class E protein of the vacuolar protein-sorting (Vps) pathway. Did2 binds Vps4p and directs it to dissociate ESCRT-III complexes.	++	++
*DID4*	Class E Vps protein of the ESCRT-III complex. Did4 is required for sorting of integral membrane proteins into lumenal vesicles of multivesicular bodies, and for delivery of newly synthesized vacuolar enzymes to the vacuole.	+	++
*SNF7*	One of four subunits of the ESCRT-III complex. Snf1 is involved in the sorting of transmembrane proteins into the multivesicular body (MVB) pathway.	+++	+++
*SNF8*	Component of the ESCRT-II complex.	+++	+++

**Table 6:** Transcription factors (TF) identified only in the methanol dataset ^(1)^, the ethanol-specific TFs ^(2)^, and the TFs shared between methanol and ethanol datasets ^(1,2)^. Symbols are as in Table 1.

Gene/ORF	Description of the Encoded Protein Function	Susceptibility to Methanol	Susceptibility to Ethanol
*CBF1* ^(1,2)^	Transcription factor that associates with kinetochore proteins, required for chromosome segregation.	+	++
*GLN3* ^(1)^	Transcriptional activator of genes regulated by nitrogen catabolite repression.	+	No phenotype
*HAP5* ^(2)^	Transcription factor that is a subunit of the Hap2/3/4/5 CCAAT-binding complex. Hap5 is a global regulator of respiratory gene expression.	No phenotype	+
*IXR1* ^(1)^	Transcriptional repressor that regulates hypoxic genes during normoxia; involved in the aerobic repression of genes such as *COX5*b, *TIR1*, and *HEM13*.	+++	No phenotype
*MGA2* ^(2)^	Transcription factor, localized in the endoplasmic reticulum membrane, involved in regulation of *OLE1* transcription.	No phenotype	++
*NGG1* ^(1)^	Transcriptional regulator involved in glucose repression of Gal4-regulated genes. Subunit of chromatin modifying histone acetyltransferase complexes.	++	No phenotype
*OAF1* ^(2)^	Transcription factor that is an activator of beta-oxidation of fatty acids, peroxisome organization and biogenesis, activating transcription in the presence of oleate.	No phenotype	+
*OPI1* ^(1)^	Transcriptional regulator of a variety of genes. Opi1 phosphorylation by protein kinase A stimulates Opi1 function in negative regulation of phospholipid biosynthetic genes.	+++	No phenotype
*RPH1* ^(1)^	Transcription factor with JmjC domain-containing histone demethylase. Rph1 targets tri- and dimethylated H3K36 and associates with actively transcribed regions and promotes elongation; also involved in the repression of autophagy-related genes in nutrient-replete conditions.	+	No phenotype
*RPN4* ^(1,2)^	Transcription factor that stimulates expression of proteasome encoding genes being regulated by the 26S proteasome in a negative feedback control mechanism.	+	+++
*RSF2* ^(2)^	Zinc-finger transcription factor that regulates both nuclear and mitochondrial genes, involved in glycerol-based growth and respiration.	No phenotype	+
*SFL1* ^(1,2)^	Transcriptional repressor and activator; involved in repression of flocculation-related genes.	++	++
*SFP1* ^(1,2)^	Transcription factor that regulates ribosomal protein and biogenesis genes; also involved in the regulation of the response to nutrients and stress, G2/M transitions during mitotic cell cycle and DNA-damage response and modulates cell size.	++	+++
*STP1* ^(2)^	Transcription factor that activates transcription of amino acid permease genes and may have a role in tRNA processing.	No phenotype	+
*SOK2* ^(1)^	Transcription factor that negatively regulates pseudohyphal differentiation; also involved in the regulation of cyclic AMP (cAMP)-dependent protein kinase signal transduction pathway.	++	No phenotype
*STB5* ^(1)^	Transcription factor involved in the regulation multidrug resistance and oxidative stress response.	++	No phenotype
*SWI6* ^(2)^	Transcription cofactor involved in meiotic gene expression. Swi6 is also required for the unfolded protein response.	No phenotype	++
*TUP1* ^(2)^	General repressor of transcription, through interactions with histones H3 and H4 and stabilization of nucleosomes over promoters.	No phenotype	+
*UME6* ^(1,2)^	Transcriptional regulator of early meiotic genes; involved in chromatin remodelling and transcriptional repression via DNA looping.	++	+++
*UPC2* ^(2)^	Transcription factor that induces sterol biosynthetic genes, upon sterol depletion. Upc2 acts as a sterol sensor, binding ergosterol in sterol rich conditions.	No phenotype	+
*URE2* ^(2)^	Transcription factor involved in the regulation of nitrogen catabolite repression.	No phenotype	+

## Data Availability

Data is available in this article and as Supplementary Material (www.mdpi.com/xxx/s1).

## Supplementary Materials

The following are available online at https://www.mdpi.com/2309-608X/7/2/90/s1, Figure S1. Visual description of the criteria used to define the different levels of susceptibility to methanol of the deletion mutant strains tested. Figure S2. Effect of methanol in the growth curves of the parental strain BY4741 (circles), and derived *ixr1*Δ (hexagons) and *opi1*Δ (squares) mutants in YPD liquid medium, (A) or in this medium supplemented with 8% (*v*/*v*) methanol (B) at 35 °C with orbital agitation (250 rpm). Table S1. List of genes whose expression increases *S. cerevisiae* tolerance to 8% (*v*/*v*) methanol based on the screening of the Euroscarf deletion mutant collection; the elimination of the indicated genes increases yeast susceptibility to methanol, Table S2. List of genes whose expression increases *S. cerevisiae* tolerance to 5.5% (*v*/*v*) ethanol based on the screening of the Euroscarf deletion mutant collection; the elimination of the indicated genes increases yeast susceptibility to ethanol.

## References

[1] Zhang W., Song M., Yang Q., Dai Z., Zhang S., Xin F., Dong W., Ma J., Jiang M. (2018). Current advance in bioconversion of methanol to chemicals. Biotechnol. Biofuels.

[2] Zhu T., Zhao T., Bankefa O.E., Li Y. (2020). Engineering unnatural methylotrophic cell factories for methanol-based biomanufacturing: Challenges and opportunities. Biotechnol. Adv..

[3] Fabarius J.T., Wegat V., Roth A., Sieber V. (2020). Synthetic Methylotrophy in Yeasts: Towards a Circular Bioeconomy. Trends Biotechnol..

[4] Frazão C.J.R., Walther T. (2020). Syngas and Methanol-Based Biorefinery Concepts. Chem. Ing. Tech..

[5] Vartiainen E., Blomberg P., Ilmén M., Andberg M., Toivari M., Penttilä M. (2019). Evaluation of synthetic formaldehyde and methanol assimilation pathways in *Yarrowia lipolytica*. Fungal Biol. Biotechnol..

[6] Chen Z., Liu D. (2016). Toward glycerol biorefinery: Metabolic engineering for the production of biofuels and chemicals from glycerol. Biotechnol. Biofuels.

[7] Yapo B.M., Lerouge P., Thibault J.F., Ralet M.C. (2007). Pectins from citrus peel cell walls contain homogalacturonans homogenous with respect to molar mass, rhamnogalacturonan I and rhamnogalacturonan II. Carbohydr. Polym..

[8] Müller-Maatsch J., Bencivenni M., Caligiani A., Tedeschi T., Bruggeman G., Bosch M., Petrusan J., Van Droogenbroeck B., Elst K., Sforza S. (2016). Pectin content and composition from different food waste streams. Food Chem..

[9] Martins L.C., Monteiro C.C., Semedo P.M., Sá-Correia I. (2020). Valorisation of pectin-rich agro-industrial residues by yeasts: Potential and challenges. Appl. Microbiol. Biotechnol..

[10] Dai Z., Gu H., Zhang S., Xin F., Zhang W., Dong W., Ma J., Jia H., Jiang M. (2017). Metabolic construction strategies for direct methanol utilization in *Saccharomyces cerevisiae*. Bioresour. Technol..

[11] Duan X., Gao J., Zhou Y.J. (2018). Advances in engineering methylotrophic yeast for biosynthesis of valuable chemicals from methanol. Chin. Chem. Lett..

[12] Dos Santos S.C., Teixeira M.C., Cabrito T.R., Sá-Correia I. (2012). Yeast toxicogenomics: Genome-wide responses to chemical stresses with impact in environmental health, pharmacology, and biotechnology. Front. Genet..

[13] Dos Santos S.C., Sá-Correia I. (2015). Yeast toxicogenomics: Lessons from a eukaryotic cell model and cell factory. Curr. Opin. Bio-Technol..

[14] Yasokawa D., Murata S., Iwahashi Y., Kitagawa E., Nakagawa R., Hashido T., Iwahashi H. (2010). Toxicity of methanol and formaldehyde towards *Saccharomyces cerevisiae* as assessed by DNA microarray analysis. Appl. Biochem. Biotechnol..

[15] Espinosa M.I., Williams T.C., Pretorius I.S., Paulsen I.T. (2019). Benchmarking two *Saccharomyces cerevisiae* laboratory strains for growth and transcriptional response to methanol. Synth. Syst. Biotechnol..

[16] North M., Gaytán B.D., Romero C., De La Rosa V.Y., Loguinov A., Smith M.T., Zhang L., Vulpe C.D. (2016). Functional toxicogenomic profiling expands insight into modulators of formaldehyde toxicity in yeast. Front. Genet..

[17] De Graaf B., Clore A., McCullough A.K. (2009). Cellular pathways for DNA repair and damage tolerance of formaldehyde-induced DNA-protein crosslinks. DNA Repair.

[18] Henriques S.F., Mira N.P., Sá-Correia I. (2017). Genome-wide search for candidate genes for yeast robustness improvement against formic acid reveals novel susceptibility (Trk1 and positive regulators) and resistance (Haa1-regulon) determinants. Biotechnol. Biofuels.

[19] Mira N.P., Becker J.D., Sá-Correia I. (2010). Genomic Expression Program Involving the Haa1p-Regulon in *Saccharomyces cerevisiae* Response to Acetic Acid. Omics J. Integr. Biol..

[20] Auesukaree C., Damnernsawad A., Kruatrachue M., Pokethitiyook P., Boonchird C., Kaneko Y., Harashima S. (2009). Genome-wide identification of genes involved in tolerance to various environmental stresses in *Saccharomyces cerevisiae*. J. Appl. Genet..

[21] Fujita K., Matsuyama A., Kobayashi Y., Iwahashi H. (2006). The genome-wide screening of yeast deletion mutants to identify the genes required for tolerance to ethanol and other alcohols. FEMS Yeast Res..

[22] Teixeira M.C., Raposo L.R., Mira N.P., Lourenço A.B., Sá-Correia I. (2009). Genome-wide identification of *Saccharomyces cerevisiae* genes required for maximal tolerance to ethanol. Appl. Environ. Microbiol..

[23] Van Voorst F., Houghton-Larson J., Jønson L., Kielland-Brandt M.C., Brandt A. (2006). Genome-wide identification of genes required for growth of *Saccharomyces cerevisiae* under ethanol stress. Yeast.

[24] Kubota S., Takeo I., Kume K., Kanai M., Atsunori S., Mizunuma M., Miyakawa T., Shimoi H., Iefuji H., Hirata D. (2004). Effect of Ethanol on Cell Growth of Budding Yeast: Genes That Are Important for Cell Growth in the Presence of Ethanol. Biosci. Biotechnol. Biochem..

[25] Yoshikawa K., Tanaka T., Furusawa C., Nagahisa K., Hirasawa T., Shimizu H. (2009). Comprehensive phenotypic analysis for identification of genes affecting growth under ethanol stress in *Saccharomyces cerevisiae*. FEMS Yeast Res..

[26] Rosa M., Sá-Correia I. (1991). In vivo activation by ethanol of plasma membrane ATPase of *Saccharomyces cerevisiae*. Appl. Environ. Microbiol..

[27] Monteiro G.A., Sá-Correia I. (1998). In vivo activation of yeast plasma membrane H+-ATPase by ethanol: Effect on the kinetic parameters and involvement of the carboxyl-terminus regulatory domain. Biochim. Biophys. Acta-Biomembr..

[28] Ogawa Y., Nitta A., Uchiyama H., Imamura T., Shimoi H., Ito K. (2000). Tolerance mechanism of the ethanol-tolerant mutant of sake yeast. J. Biosci. Bioeng..

[29] Aguilera F., Peinado R.A., Millán C., Ortega J.M., Mauricio J.C. (2006). Relationship between ethanol tolerance, H+-ATPase activity and the lipid composition of the plasma membrane in different wine yeast strains. Int. J. Food Microbiol..

[30] Salgueiro S.P., Sá-Correia I., Novais J.M. (1988). Ethanol-Induced Leakage in *Saccharomyces cerevisiae*: Kinetics and Relationship to Yeast Ethanol Tolerance and Alcohol Fermentation Productivity. Appl. Environ. Microbiol..

[31] Stanley D., Bandara A., Fraser S., Chambers P.J., Stanley G.A. (2010). The ethanol stress response and ethanol tolerance of *Saccharomyces cerevisiae*. J. Appl. Microbiol..

[32] Chi Z., Arneborg N. (1999). Relationship between lipid composition, frequency of ethanol-induced respiratory deficient mutants, and ethanol tolerance in *Saccharomyces cerevisiae*. J. Appl. Microbiol..

[33] You K.M., Rosenfield C., Knipple D.C. (2003). Ethanol tolerance in the yeast *Saccharomyces cerevisiae* is dependent on cellular oleic acid content. Appl. Environ. Microbiol..

[34] Reggiori F., Klionsky D.J. (2013). Autophagic processes in yeast: Mechanism, machinery and regulation. Genetics.

[35] Delorme-Axford E., Popelka H., Klionsky D.J. (2019). TEX264 is a major receptor for mammalian reticulophagy. Autophagy.

[36] Martínez-Muñoz G.A., Kane P. (2008). Vacuolar and plasma membrane proton pumps collaborate to achieve cytosolic pH homeostasis in yeast. J. Biol. Chem..

[37] Charoenbhakdi S., Dokpikul T., Burphan T., Techo T., Auesukaree C. (2016). Vacuolar H+-ATPase protects *Saccharomyces cerevisiae* cells against ethanol induced oxidative and cell wall stresses. Appl. Environ. Microbiol..

[38] Monteiro P.T., Oliveira J., Pais P., Antunes M., Palma M., Cavalheiro M., Galocha M., Godinho C.P., Martins L.C., Bourbon N. (2020). YEASTRACT+: A portal for cross-species comparative genomics of transcription regulation in yeasts. Nucleic Acids Res..

[39] Skrzydlewska E. (2003). Toxicological and Metabolic Consequences of Methanol Poisoning. Toxicol. Mech. Methods.

[40] Gasch A.P., Spellman P.T., Kao C.M., Carmel-Harel O., Eisen M.B., Storz G., Botstein D., Brown P.O. (2000). Genomic Expression Programs in the Response of Yeast Cells to Environmental Changes. Mol. Biol. Cell.

[41] Estruch F. (2000). Stress-controlled transcription factors, stress-induced genes and stress tolerance in budding yeast. FEMS Microbiol. Rev..

[42] Chua G., Morris Q.D., Sopko R., Robinson M.D., Ryan O., Chan E.T., Frey B.J., Andrews B.J., Boone C., Hughes T.R. (2006). Identifying transcription factor functions and targets by phenotypic activation. Proc. Natl. Acad. Sci. USA.

[43] Reimand J., Vaquerizas J.M., Todd A.E., Vilo J., Luscombe N.M. (2010). Comprehensive reanalysis of transcription factor knockout expression data in *Saccharomyces cerevisiae* reveals many new targets. Nucleic Acids Res..

[44] Vizoso-Vázquez Á., Lamas-Maceiras M., González-Siso M.I., Cerdán M.E. (2018). Ixr1 Regulates Ribosomal Gene Transcription and Yeast Response to Cisplatin. Sci. Rep..

[45] Vizoso-Vázquez Á., Lamas-Maceiras M., Becerra M., González-Siso M.I., Rodríguez-Belmonte E., Cerdán M.E. (2012). Ixr1p and the control of the *Saccharomyces cerevisiae* hypoxic response. Appl. Microbiol. Biotechnol..

[46] Game J.C., Mortimer R.K. (1974). A Genetic Study of X-Ray Sensitive Mutants in Yeast. Mutat. Res..

[47] Lewis K.L., Karthikeyan G., Westmoreland J.W., Resnick M.A. (2002). Differential suppression of DNA repair deficiencies of yeast *rad50*, *mre11* and *xrs2* mutants by *EXO1* and *TLC1* (the RNA component of telomerase). Genetics.

[48] Symington L.S. (2002). Role of *RAD52* epistasis group genes in homologous recombination and double-strand break repair. Microbiol. Mol. Biol. Rev..

[49] Salter G.J., Kell D.B. (1995). Solvent Selection for Whole Cell Biotransformations in Organic Media. Crit. Rev. Biotechnol..

[50] Sangster J. (1989). Octanol-water partition coefficients of simple organic compounds. J. Phys. Chem..

[51] Weber F.J., De Bont J.A.M. (1996). Adaptation mechanisms of microorganisms to the toxic effects of organic solvents on membranes. Biochim. Biophys. Acta.

[52] Sikkema J., De Bont J.A.M., Poolman B. (1995). Mechanisms of membrane toxicity of hydrocarbons. Microbiol. Rev..

[53] Jackson J.C., Lopes J.M. (1996). The yeast *UME6* gene is required for both negative and positive transcriptional regulation of phospholipid biosynthetic gene expression. Nucleic Acids Res..

[54] Leão C., van Uden N. (1984). Effects of ethanol and other alkanols on passive proton influx in the yeast *Saccharomyces cerevisiae*. Biochim. Biophys. Acta (BBA)-Biomembr..

[55] Rosa M.F., Sá-Correia I. (1996). Intracellular acidification does not account for inhibition of *Saccharomyces cerevisiae* growth in the presence of ethanol. FEMS Microbiol. Lett..

[56] Carmelo V., Santos H., Sá-Correia I. (1997). Effect of extracellular acidification on the activity of plasma membrane ATPase and on the cytosolic and vacuolar pH of *Saccharomyces cerevisiae*. Biochim. Biophys. Acta-Biomembr..

[57] Welsh D.T. (2000). Ecological significance of compatible solute accumulation by micro- organisms: From single cells to global climate. FEMS Microbiol. Rev..

[58] Wiemken A. (1990). Trehalose in yeast, stress protectant rather than reserve carbohydrate. Antonie Van Leeuwenhoek.

[59] Kroemer G., Mariño G., Levine B. (2010). Autophagy and the integrated stress response. Mol. Cell.

[60] Miyagawa K.I., Ishiwata-Kimata Y., Kohno K., Kimata Y. (2014). Ethanol stress impairs protein folding in the endoplasmic reticulum and activates Ire1 in *Saccharomyces cerevisiae*. Biosci. Biotechnol. Biochem..

[61] Chen S.T., Chen S.Y., Tu C.C., Chiou S.H., Wang K.T. (1995). Physicochemical properties of alkaline serine proteases in alcohol. J. Protein Chem..

[62] Bolt H.M. (1987). Experimental toxicology of formaldehyde. J. Cancer Res. Clin. Oncol..

[63] Banfield D.K. (2011). Mechanisms of protein retention in the Golgi. Cold Spring Harb. Perspect. Biol..

[64] Ma M., Liu Z.L. (2010). Comparative transcriptome profiling analyses during the lag phase uncover *YAP1,PDR1,PDR3 RPN4* and *HSF1* as key regulatory genes in genomic adaptation to the lignocellulose derived inhibitor HMF for *Saccharomyces cerevisiae*. BMC Genom..

[65] Chen Y., Sheng J., Jiang T., Stevens J., Feng X., Wei N. (2016). Transcriptional profiling reveals molecular basis and novel genetic targets for improved resistance to multiple fermentation inhibitors in *Saccharomyces cerevisiae*. Biotechnol. Biofuels.

[66] Dohmen R.J., Willers I., Marques A.J. (2007). Biting the hand that feeds: Rpn4-dependent feedback regulation of proteasome function. Biochim. Biophys. Acta-Mol. Cell Res..

[67] Alexandre H., Ansanay-Galeote V., Dequin S., Blondin B. (2001). Global gene expression during short-term ethanol stress in *Saccharomyces cerevisiae*. FEBS Lett..

[68] Bubis J.A., Spasskaya D.S., Gorshkov V.A., Kjeldsen F., Kofanova A.M., Lekanov D.S., Gorshkov M.V., Karpov V.L., Tarasova I.A., Karpov D.S. (2020). Rpn4 and proteasome-mediated yeast resistance to ethanol includes regulation of autophagy. Appl. Microbiol. Biotechnol..

[69] Marion R.M., Regev A., Segal E., Barash Y., Koller D., Friedman N., Shea E.K.O. (2004). Sfp1 is a stress- and nutrient-sensitive regulator of ribosomal protein gene expression. Proc. Natl. Acad. Sci. USA.

[70] Swinnen S., Henriques S.F., Shrestha R., Ho P.-W., Sá-Correia I., Nevoigt E. (2017). Improvement of yeast tolerance to acetic acid through Haa1 transcription factor engineering: Towards the underlying mechanisms. Microb. Cell Fact..

[71] Li B., Wang L., Wu Y., Xia Z., Yang B., Tang Y. (2020). Improving acetic acid and furfural resistance of *Saccharomyces cerevisiae* by regulating novel transcriptional factors revealed via comparative transcriptome. Authorea Prepr..

[72] Wang L., Wang X., He Z.Q., Zhou S.J., Xu L., Tan X.Y., Xu T., Li B.Z. (2020). Engineering prokaryotic regulator IrrE to enhance stress tolerance in budding yeast. Biotechnol. Biofuels.

[73] Scherens B., Feller A., Vierendeels F., Messenguy F., Dubois E. (2006). Identification of direct and indirect targets of the Gln3 and Gat1 activators by transcriptional profiling in response to nitrogen availability in the short and long term. FEMS Yeast Res..

[74] Eberharter A., Sterner D.E., Schieltz D., Hassan A., Yates J.R., Berger S.L., Workman J.L. (1999). The ADA Complex Is a Distinct Histone Acetyltransferase Complex in *Saccharomyces cerevisiae*. Mol. Cell. Biol..

[75] Klose R.J., Gardner K.E., Liang G., Erdjument-Bromage H., Tempst P., Zhang Y. (2007). Demethylation of Histone H3K36 and H3K9 by Rph1: A Vestige of an H3K9 Methylation System in *Saccharomyces cerevisiae*?. Mol. Cell. Biol..

[76] Sreenivas A., Carman G.M. (2003). Phosphorylation of the yeast phospholipid synthesis regulatory protein Opi1p by Protein Kinase A. J. Biol. Chem..

[77] Bernard A., Jin M., González-Rodríguez P., Füllgrabe J., Backues S.K., Joseph B., Klionsky D.J. (2015). Rph1/KDM4 mediates nutrient-limitation signaling that leads to the transcriptional induction of autophagy. Curr. Biol..

[78] Pan X., Heitman J. (2000). Sok2 Regulates Yeast Pseudohyphal Differentiation via a Transcription Factor Cascade That Regulates Cell-Cell Adhesion. Mol. Cell. Biol..

[79] Larochelle M., Drouin S., Robert F., Turcotte B. (2006). Oxidative Stress-Activated Zinc Cluster Protein Stb5 Has Dual Activa-tor/Repressor Functions Required for Pentose Phosphate Pathway Regulation and NADPH Production. Mol. Cell. Biol..

[80] Tsaponina O., Chabes A. (2013). Pre-activation of the genome integrity checkpoint increases DNA damage tolerance. Nucleic Acids Res..

[81] Castro-Prego R., Lamas-Maceiras M., Soengas P., Carneiro I., González-Siso I., Cerdán M.E. (2010). Regulatory factors controlling transcription of *Saccharomyces cerevisiae IXR1* by oxygen levels: A model of transcriptional adaptation from aerobiosis to hypoxia implicating *ROX1* and *IXR1* cross-regulation. Biochem. J..

[82] Luévano-Martínez L.A., Appolinario P., Miyamoto S., Uribe-carvajal S., Kowaltowski A.J. (2013). Deletion of the transcriptional regulator opi1p decreases cardiolipin content and disrupts mitochondrial metabolism in *Saccharomyces cerevisiae*. Fungal Genet. Biol..

[83] Jiménez J., Benítez T. (1988). Yeast cell viability under conditions of high temperature and ethanol concentrations depends on the mitochondrial genome. Curr. Genet..

[84] Iwaki M., Rich P.R. (2004). Direct Detection of Formate Ligation in Cytochrome c Oxidase by ATR-FTIR Spectroscopy. J. Am. Chem. Soc..

[85] Nicholls P. (1975). Formate as an inhibitor of cytochrome c oxidase. Biochem. Biophys. Res. Commun..

[86] Lin D. (2012). Toxicity mechanism of formic acid is directly linked to ROS burst and oxidative damage in yeast *Saccharomyces cerevisiae*. Adv. Mater. Res..

[87] Guo Z.P., Olsson L. (2016). Physiological responses to acid stress by *Saccharomyces cerevisiae* when applying high initial cell density. FEMS Yeast Res..

[88] Ashoori M., Saedisomeolia A. (2014). Riboflavin (vitamin B2) and oxidative stress: A review. Br. J. Nutr..

[89] Wojtczak L., Slyshenkov V.S. (2003). Protection by pantothenic acid against apoptosis and cell damage by oxygen free radicals–The role of glutathione. BioFactors.

[90] Stolz J., Hoja U., Meier S., Sauer N., Schweizer E. (1999). Identification of the Plasma Membrane H+-Biotin Symporter of *Saccharomyces cerevisiae* by Rescue of a Fatty Acid-auxotrophic Mutant. J. Biol. Chem..

[91] Wang Y., Fan L., Tuyishime P., Liu J., Zhang K., Gao N., Zhang Z., Ni X., Feng J., Yuan Q. (2020). Adaptive laboratory evolution enhances methanol tolerance and conversion in engineered *Corynebacterium glutamicum*. Commun. Biol..

[92] Reihl P., Stolz J. (2005). The Monocarboxylate Transporter Homolog Mch5p Catalyzes Riboflavin (Vitamin B2) Uptake in *Saccharomyces cerevisiae*. J. Biol. Chem..

[93] Brandt B.A., Garcia-Aparicio M., Mokomele T., Gorgens J.F., van Zyl W.H. (2020). Rational engineering of *Saccharomyces cerevisiae* towards improved tolerance to multiple inhibitors in lignocellulose fermentations. Biotechnol. Biofuels.

