# Supplementary material for: The Identification of Genetic Determinants of Methanol Tolerance in Yeast Suggests Differences in Methanol and Ethanol Toxicity Mechanisms and Candidates for Improved Methanol Tolerance Engineering
Source: J Fungi (Basel). 2021 Jan 27;7(2):90. doi: 10.3390/jof7020090 (PMC7911966; doi:10.3390/jof7020090)
Supplement: Supplementary file 1 [file jof-07-00090-s001.pdf]

Supplementary material

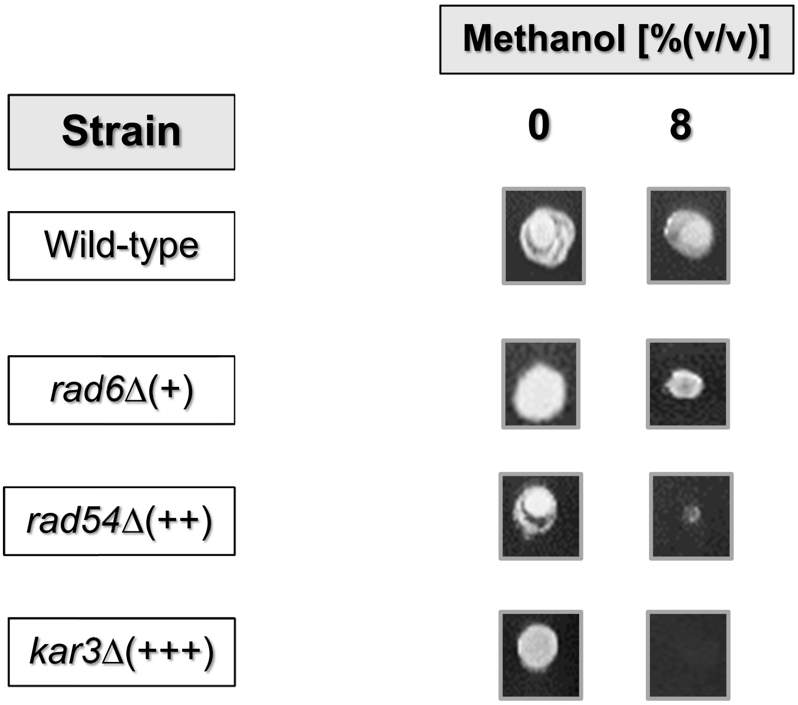

Figure S1. Visual description of the criteria used to define the different levels of susceptibility to methanol of the deletion mutant strains tested. Wild-type and deletion mutant strains were spotted onto solid YPD medium (pH 4.5) supplemented with 8%(v/v) of methanol. Three levels of susceptibility were defined when the growth of the single mutant strains was slightly diminished compared to wild type (+), significantly reduced (++), or abolished (+++).

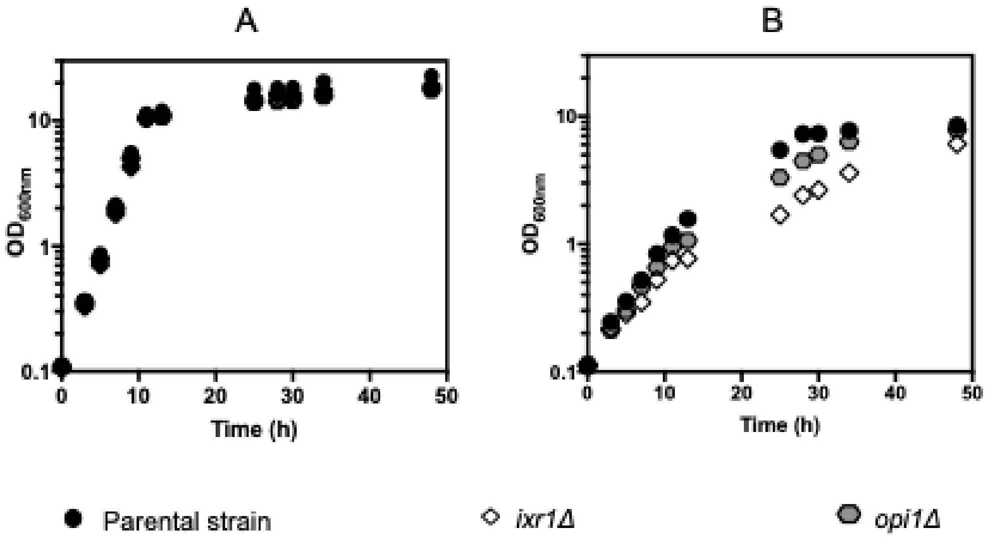

Figure S2. Effect of methanol in the growth curves of the parental strain BY4741 (circles), and derived *ixr1*Δ (hexagons) and *opi1*Δ (squares) mutants in YPD liquid medium, **(A)** or in this medium supplemented with 8%(v/v) methanol(**B)** at 35ºC with orbital agitation (250 rpm). The growth curves are representative of at least three independent experiments.

Table S1. List of genes whose expression increases *S. cerevisiae* tolerance to 8%(v/v) methanol based on the screening of the Euroscarf deletion mutant collection; the elimination of the indicated genes increases yeast susceptibility to methanol. Biological functions are based on the information available in SGD (www.yeastgenome.org). The symbols '+++', '++' and '+' refer to phenotypes as shown in Figure S1.

Ammonium and vitamin metabolism

Genes	Function	Susceptibility to methanol
BNA1	3-hydroxyanthranilic acid dioxygenase. Bna1 is required for the *de novo* biosynthesis of NAD from tryptophan via kynurenine.	++
ADO1	Adenosine kinase. Ado1 is required for the utilization of S-adenosylmethionine.	+++
MET7	Folylpolyglutamate synthetase. Met7 catalyses extension of the glutamate chains of the folate coenzymes, required for methionine synthesis and for maintenance of mitochondrial DNA.	+
NPR3	Subunit of the Iml1/SEACIT complex; SEACIT (Iml1-Npr2-Npr3) that is required for non-nitrogen-starvation-induced autophagy.	+
PDX3	Pyridoxine (pyridoxamine) phosphate oxidase.	++

Autophagy

Genes	Function	Susceptibility to methanol
ATG11	Adapter protein for pexophagy and the Cvt targeting pathway.	+++
ATG15	Phospholipase; Atg15 is required for lysis of autophagic and CVT bodies.	++
MON1	Subunit of a heterodimeric guanine nucleotide exchange factor (GEF). Mon1 is a subunit of the Mon1-Ccz1 GEF complex which stimulates nucleotide exchange and activation of Ypt7, a Rab family GTPase involved in membrane tethering and fusion events at the late endosome and vacuole.	++

Carbohydrate metabolism

Genes	Function	Susceptibility to methanol
GPH1	Glycogen phosphorylase required for the mobilization of glycogen.	+++
LPD1	Lipoamide dehydrogenase component (E3) of the pyruvate dehydrogenase and 2-oxoglutarate dehydrogenase multi-enzyme complexes.	+++
MAN2	Mannitol dehydrogenase.	+
MLS1	Malate synthase.	+
PDA1	E1 alpha subunit of the pyruvate dehydrogenase (PDH) complex.	+
PDB1	E1 beta subunit of the pyruvate dehydrogenase (PDH) complex.	+
RPE1	D-ribulose-5-phosphate 3-epimerase.	++
TPS1	Synthase subunit of trehalose-6-P synthase/phosphatase complex.	++
TPS2	Phosphatase subunit of the trehalose-6-P synthase/phosphatase complex.	+++

Cell cycle

Genes	Function	Susceptibility to methanol
BNI5	Linker protein responsible for recruitment of myosin to the bud neck.	+
BUB3	Kinetochore checkpoint WD40 repeat protein.	++
CDC55	Regulatory subunit B of protein phosphatase 2A (PP2A).	+++
CIK1	Kinesin-associated protein. Cik1 is required for both karyogamy and mitotic spindle organization.	+++
CIN8	Kinesin motor protein involved in mitotic spindle assembly and chromosome segregation.	++
IBD2	Component of the BUB2-dependent spindle checkpoint pathway.	+
RTS1	B-type regulatory subunit of protein phosphatase 2A (PP2A).	+
SHS1	Component of the septin ring that is required for cytokinesis.	+++
SWM1	Subunit of the anaphase-promoting complex (APC); APC is an E3 ubiquitin ligase that regulates the metaphase-anaphase transition and exit from mitosis.	+++

Cell wall function

Genes	Function	Susceptibility to methanol
CCW12	Cell wall mannoprotein. Ccw12 plays a role in maintenance of newly synthesized areas of cell wall.	++
CWH41	Processing alpha glucosidase I and ER type II integral membrane N-glycoprotein involved in assembly of cell wall beta 1,6 glucan and asparagine-linked protein glycosylation.	+
FKS1	Catalytic subunit of 1,3-beta-D-glucan synthase. Fks1 is involved in cell wall synthesis and maintenance.	+++
GAS1	Beta-1,3-glucanosyltransferase. Gas1 is required for cell wall assembly and also has a role in transcriptional silencing.	++
KRE6	Type II integral membrane protein. Kre6 is required for beta-1,6 glucan biosynthesis.	++
MNN11	Subunit of a Golgi mannosyltransferase complex that mediates elongation of the polysaccharide mannan backbone.	+++
PEF1	Penta-EF-hand protein, required for polar bud growth and cell wall abscission.	++
ROT2	Glucosidase II catalytic subunit. Rot2 is required to trim the final glucose in N-linked glycans and for normal cell wall synthesis.	+++
SLG1	Sensor-transducer of the stress-activated PKC1-MPK1 kinase pathway. Slg1 is involved in maintenance of cell wall integrity.	++
SMI1	Protein involved in the regulation of cell wall synthesis.	+++

Cellular signalling

Genes	Function	Susceptibility to methanol
ASC1	G-protein beta subunit and guanine dissociation inhibitor for Gpa2 required to prevent frameshifting at ribosomes stalled at repeated CGA codons.	+
BCK1	MAPKKK acting in the protein kinase C signalling pathway, controlling cell integrity.	++

CLA4	Cdc42-activated signal transducing kinase. Cla4 is involved in septin ring assembly, vacuole inheritance, cytokinesis, sterol uptake regulation.	++
ELM1	Serine/threonine protein kinase. Elm1 regulates the orientation checkpoint, the morphogenesis checkpoint and the metabolic switch from fermentative to oxidative metabolism.	++
MKK1	MAPKK involved in the protein kinase C signalling pathway, controlling of cell integrity.	+
NBP2	Protein involved in the HOG (high osmolarity glycerol) pathway; negatively regulates Hog1 by recruitment of phosphatase Ptc1 to the Pbs2-Hog1 complex.	++
PCL6	Pho85p cyclin of the Pho80p subfamily. Pcl6 is involved in the control of glycogen storage by Pho85.	+
RAS2	GTP-binding protein that regulates nitrogen starvation response, sporulation, and filamentous growth.	+++
ROM2	Guanine nucleotide exchange factor (GEF) for Rho1 and Rho2.	+
SNF4	Activating gamma subunit of the AMP-activated Snf1 kinase complex; activates glucose-repressed genes, represses glucose-induced genes; role in sporulation, and peroxisome biogenesis.	++
SST2	GTPase-activating protein for Gpa1. Sst2 regulates desensitization to alpha factor pheromone.	++
TUS1	Guanine nucleotide exchange factor (GEF) that modulates Rho1 activity. Tus1 is involved in the cell integrity signalling pathway.	++
YPT7	Rab family GTPase; GTP-binding protein of the rab family. Ypt7 is required for homotypic fusion event in vacuole inheritance, for endosome-endosome fusion.	++

Chromatin remodelling, nucleic acid metabolism and transcription

Genes	Function	Susceptibility to methanol
ADA2	Transcription coactivator; component of the ADA and SAGA transcriptional adaptor/HAT (histone acetyltransferase) complexes.	++
AMD1	AMP deaminase that catalyses the deamination of AMP to form IMP and ammonia.	+
ARP8	Nuclear actin-related protein involved in chromatin remodelling.	+
ASF1	Nucleosome assembly factor. Asf1 is involved in chromatin assembly, disassembly and in the recovery after DSB repair.	++
BRE1	E3 ubiquitin ligase. Bre1 forms heterodimer with Rad6 to regulate K63 polyubiquitination in response to oxidative stress and to monoubiquinate histone H2B-K123, which is required for the subsequent methylation of histone H3-K4 and H3-K79.	++
CBP2	Required for splicing of the group I intron bI5 of the COB pre-mRNA;	+
CDC73	Component of the Paf1 complex. Cdc73 binds to and modulates the activity of RNA polymerases I and II. Cdc73 is also involved in transcription elongation.	++
CHZ1	Histone chaperone for Htz1/H2A-H2B dimer.	+
CSE2	Subunit of the RNA polymerase II mediator complex.	++
CSM1	Nucleolar protein that mediates homolog segregation during meiosis I.	+++
DIA2	Origin-binding F-box protein that functions in ubiquitination of silent chromatin structural protein Sir4. Dia2 is required to target Cdc6 for destruction during G1 phase and is also required for deactivation of Rad53 checkpoint kinase, completion of DNA	++

	replication during recovery from DNA damage, assembly of RSC complex, RSC-mediated transcription regulation, and nucleosome positioning.	
DST1	General transcription elongation factor TFIIS. Dst1 enables RNA polymerase II to read through blocks to elongation by stimulating cleavage of nascent transcripts stalled at transcription arrest sites.	+
ELP3	Subunit of Elongator complex; Elongator is required for modification of wobble nucleosides in tRNA.	+
GCN5	Catalytic subunit of ADA and SAGA histone acetyltransferase complexes.	+
GTF1	Subunit of the trimeric GatFAB AmidoTransferase(AdT) complex. Gtf1 is involved in the formation of Q-tRNAQ.	+
HIR3	Subunit of the HIR complex, Hir3 is involved in regulation of histone gene transcription,	+
HTA1	Histone H2A. Hta1 is required for chromatin assembly and chromosome function.	++
HTZ1	Histone variant H2AZ. Htz1 is involved in transcriptional regulation through prevention of the spread of silent heterochromatin.	++
IKI1	Subunit of hexameric RecA-like ATPase Elp456 Elongator subcomplex. Iki1 is required for modification of wobble nucleosides in tRNA.	++
LSM6	Lsm (Like Sm) protein. Lsm6 is part of heteroheptameric complexes and it is involved inm RNA decay.	+
MAF1	Negative regulator of RNA polymerase III that is involved in tRNA processing and stability.	++
MED1	Subunit of the RNA polymerase II mediator complex. Med1 is essential for transcriptional regulation.	++
MED2	Subunit of the RNA polymerase II mediator complex. Med2 is essential for transcriptional regulation.	++
MFT1	Subunit of the THO complex that is involved in transcription elongation and mitotic recombination.	++
MOG1	Nuclear protein that interacts with GTP-Gsp1, Mog1 stimulates nucleotide release from Gsp1 and is also involved in nuclear protein import.	+++
NCL1	S-adenosyl-L-methionine-dependent tRNA.	++
NPP1	Nucleotide pyrophosphatase/phosphodiesterase; Npp1 mediates extracellular nucleotide phosphate hydrolysis along with Npp2 and Pho5.	++
NPT1	Nicotinate phosphoribosyltransferase. Npt1 is required for silencing at rDNA and telomeres and has a role in silencing at mating-type loci.	++
NUT1	Component of the RNA polymerase II mediator complex that is required for transcriptional activation and also has a role in basal transcription.	++
PAP2	Non-canonical poly(A) polymerase. Pap2 is involved in nuclear RNA degradation as a component of TRAMP.	+
PAT1	Deadenylation-dependent mRNA-decapping factor.	+
POL32	Third subunit of DNA polymerase delta; Pol32 is involved in chromosomal DNA replication.	+
RCO1	Essential component of the Rpd3S histone deacetylase complex.	++
RFM1	Component of the Sum1-Rfm1-Hst1 complex. Rfm1 is involved in transcriptional repression of middle sporulation genes and in initiation of DNA replication.	+
RMI1	Subunit of the RecQ (Sgs1) - Top3 complex Rmi1 stimulates	++

	superhelical relaxing, DNA catenation/decatenation and ssDNA binding activities of Top3. Rmi1 is also involved in response to DNA damage;	
RRN10	Protein involved in promoting high level transcription of rDNA; subunit of UAF (upstream activation factor) for RNA polymerase I.	++
RRP6	Nuclear exosome exonuclease component that has 3'-5' exonuclease activity that is regulated by Lrp1.	+
RSC1	Component of the RSC chromatin remodelling complex. Rsc1 is required for expression of mid-late sporulation-specific genes.	++
RTT101	Cullin subunit of a Roc1-dependent E3 ubiquitin ligase complex. Rtt101 plays a role in anaphase progression and is required for recovery after DSB repair.	++
RTT109	Histone acetyltransferase. Acetylates H3K56, H3K9.	+
SGS1	RecQ family nucleolar DNA helicase. Sgs1 play a role in genome integrity maintenance, chromosome synapsis, meiotic joint molecule/crossover formation.	++
SIN3	Component of both the Rpd3S and Rpd3L histone deacetylase complexes. Sin3 is involved in transcriptional repression and activation of diverse processes, including mating-type switching and meiosis.	+
SIR3	Silencing protein. Sir3 is required for spreading of silenced chromatin.	++
SNF6	Subunit of the SWI/SNF chromatin remodelling complex. Snf6 is involved in transcriptional regulation,	++
SPT21	Protein with a role in transcriptional silencing.	++
SPT3	Subunit of the SAGA and SAGA-like transcriptional regulatory complexes.	+
SPT4	Spt4/5 (DSIF) transcription elongation factor complex subunit; the Spt4/5 complex binds to ssRNA in a sequence-specific manner, and along with RNAP I and II has multiple roles regulating transcriptional elongation, RNA processing, quality control, and transcription-coupled repair.	+
SRB8	Subunit of the RNA polymerase II mediator complex. Srb8 is essential for transcriptional regulation and is involved in glucose repression.	+
SSN8	Cyclin-like component of the RNA polymerase II holoenzyme. Ssn8 is involved in phosphorylation of the RNA polymerase II C-terminal domain.	+++
TCD2	tRNA threonylcarbamoyladenosine dehydratase.	+
THO2	Subunit of the THO complex.	+++
THP2	Subunit of the THO and TREX complexes.	+++
THP3	Protein that may have a role in transcription elongation.	+
TOP1	Topoisomerase I; relieves torsional strain in DNA by cleaving and re-sealing the phosphodiester backbone. Top1 relaxes both positively and negatively supercoiled DNA.	++
TOP3	DNA Topoisomerase III that relax single-stranded negatively-supercoiled DNA preferentially.	++
TRF5	Non-canonical poly(A) polymerase. Trf5 is involved in nuclear RNA degradation as a component of the TRAMP complex.	+
UAF30	Subunit of UAF (upstream activation factor) complex.	+++
UBP8	Ubiquitin-specific protease component of the SAGA acetylation complex; Ubp8 is required for SAGA (Spt-Ada-Gcn5-Acetyltransferase)-mediated deubiquitination of histone H2B.	+
UPF3	Component of the nonsense-mediated mRNA decay (NMD) pathway. Upf3 is involved in decay of mRNA containing nonsense codon.	+

YAF9	Subunit of NuA4 histone H4 acetyltransferase and SWR1 complexes.	+
YNG2	Subunit of NuA4, an essential histone acetyltransferase complex.	+++
YTA7	Protein that localizes to chromatin that plays a role in regulation of histone gene expression.	++

Cytoskeleton

Genes	Functions	Susceptibility to methanol
ARP6	Actin-related protein that binds nucleosomes.	+++
BEM2	Rho GTPase activating protein (RhoGAP) that involved in the control of cytoskeleton organization and cellular morphogenesis.	+++
BEM4	Protein involved in establishment of cell polarity and bud emergence.	++
BER1	Protein involved in microtubule-related processes.	++
BIK1	Microtubule-associated protein. Bik1 is a component of the interface between microtubules and kinetochore and is involved in sister chromatid separation.	+
BIM1	Microtubule plus end-tracking protein.	++
BNI1	Formin; polarisome component; nucleates the formation of linear actin filaments, involved in cell processes such as budding and mitotic spindle orientation which require the formation of polarized actin cables.	++
GIN4	Protein kinase involved in bud growth and assembly of the septin ring.	+
HOF1	Protein that regulates actin cytoskeleton organization.	+
KAR3	Minus-end-directed microtubule motor.	+++
LDB18	Component of the dynactin complex; dynactin is required for dynein activity.	++
PAC11	Dynein intermediate chain, microtubule motor protein.	++
SLA1	Cytoskeletal protein binding protein.	+++
SPC72	Gamma-tubulin small complex (gamma-TuSC) receptor.	++
VRP1	Verprolin, proline-rich actin-associated protein; involved in cytoskeletal organization and cytokinesis.	++
YKE2	Subunit of the heterohexameric Gim/prefoldin protein complex; involved in the folding of alpha-tubulin, beta-tubulin, and actin.	+++

DNA repair

Genes	Function	Susceptibility to methanol
MET18	Component of cytosolic iron-sulfur protein assembly (CIA) machinery. Met18 acts at a late step of Fe-S cluster assembly and it is also involved in DNA replication and repair, transcription, and telomere maintenance.	+++
MMS22	Subunit of E3 ubiquitin ligase complex involved in replication repair.	+++
MRE11	Nuclease subunit of the MRX complex with Rad50and Xrs2; MRX complex functions in repair of DNA double-strand breaks and in telomere stability.	+++
MTF1	Mitochondrial RNA polymerase specificity factor.	++
RAD18	E3 ubiquitin ligase; forms heterodimer with Rad6 to	++

	monoubiquitinate PCNA-K164; Rad18 is required for postreplication repair.	
RAD27	5' to 3' exonuclease, 5' flap endonuclease. Rad27 is required for Okazaki fragment processing and maturation, for long-patch base-excision repair.	+++
RAD33	Protein involved in nucleotide excision repair.	+
RAD5	DNA helicase/Ubiquitin ligase. Rad5 is involved in error-free DNA damage tolerance (DDT), replication fork regression during postreplication repair by template switching, error-prone translesion synthesis.	++
RAD51	Strand exchange protein. Rad51 is involved in the recombinational repair of double-strand breaks in DNA during vegetative growth and meiosis.	+++
RAD54	DNA-dependent ATPase that stimulates strand exchange. Rad54 is involved in the recombinational repair of double-strand breaks in DNA during vegetative growth and meiosis	++
RAD57	Protein that stimulates strand exchange by stabilizing the binding of Rad51 to single-stranded DNA. Rad57 is involved in the recombinational repair of double-strand breaks in DNA during vegetative growth and meiosis.	++
RAD6	Ubiquitin-conjugating enzyme (E2). Rad6 is involved in postreplication repair as a heterodimer with Rad18, regulation of K63 polyubiquitination in response to oxidative stress, DSBR and checkpoint control and as a heterodimer with Bre1.	+
TOF1	Subunit of a replication-pausing checkpoint complex. Tof1-Mrc1-Csm3 complex acts at the stalled replication fork to promote sister chromatid cohesion after DNA damage, facilitating gap repair of damaged DNA.	+

Internal pH homeostasis

Genes	Function	Susceptibility to methanol
PMP1	Regulatory subunit for the plasma membrane H(+)-ATPase Pma1.	++
VMA1	Subunit A of the V1 peripheral membrane domain of V-ATPase.	+++
VMA13	Subunit H of the V1 peripheral membrane domain of V-ATPase.	+++
VMA2	Subunit B of V1 peripheral membrane domain of vacuolar H^+-ATPase.	+++
VMA7	Subunit F of the V1 peripheral membrane domain of V-ATPase.	+++
VPH1	Subunit a of vacuolar-ATPase V0 domain.	+
VPH2	Integral membrane protein required for V-ATPase function.	++

Intracellular trafficking and protein sorting

Genes	Function	Susceptibility to methanol
ACB1	Acyl-CoA-binding protein that transports newly synthesized acyl-CoA esters from fatty acid synthetase (Fas1-Fas2) to acyl-CoA-consuming processes.	+
ARF1	ADP-ribosylation factor; GTPase of the Ras superfamily involved in regulation of coated vesicle formation in intracellular trafficking within the Golgi.	++
ARV1	Cortical ER protein. Arv1 is implicated in the membrane insertion of	+++

	tail-anchored C-terminal single transmembrane domain proteins.	
BMH1	14-3-3 protein, major isoform. Bmh1 controls proteome at post-transcriptional level, binds proteins and DNA and it is involved in regulation of exocytosis, vesicle transport, Ras/MAPK and rapamycin-sensitive signalling, aggresome formation, spindle position checkpoint.	+
BRO1	Cytoplasmic class E vacuolar protein sorting (VPS) factor. Bro1 coordinates deubiquitination in the multivesicular body (MVB) pathway by recruiting Doa4 to endosomes.	++
BUG1	Cis-Golgi localized protein involved in ER to Golgi transport.	++
CCR4	Component of the CCR4-NOT transcriptional complex. CCR4-NOT is involved in regulation of gene expression.	++
CLC1	Clathrin light chain and a subunit of the major coat protein involved in intracellular protein transport and endocytosis. Clc1 regulates endocytic progression.	+++
DID2	Class E protein of the vacuolar protein-sorting (Vps) pathway. Did2 binds Vps4p and directs it to dissociate ESCRT-III complexes.	++
DID4	Class E Vps protein of the ESCRT-III complex. Did4 is required for sorting of integral membrane proteins into lumenal vesicles of multivesicular bodies, and for delivery of newly synthesized vacuolar enzymes to the vacuole.	+
DOA4	Ubiquitin hydrolase. Doa4 despf1ates intralumenal vesicle (ILVs) cargo proteins and is required for recycling ubiquitin from proteasome-bound ubiquitinated intermediates.	++
END3	EH domain-containing protein involved in endocytosis.	+
GCS1	ADP-ribosylation factor GTPase activating protein (ARF GAP); involved in ER-Golgi transport.	+++
GLO3	ADP-ribosylation factor GTPase activating protein (ARF GAP) that is involved in ER-Golgi transport.	+
GSF2	Endoplasmic reticulum (ER) localized integral membrane protein that may promote secretion of certain hexose transporters.	++
GTR1	Subunit of a TORC1-stimulating GTPase and the EGO/GSE complex. Gtr1 is also a subunit of Gtr1-Gtr2, a GTPase that activates TORC1 in response to amino acid stimulation.	+
LDB19	Alpha-arrestin involved in ubiquitin-dependent endocytosis.	++
PEP12	Target membrane receptor (t-SNARE); for vesicular intermediates traveling between the Golgi apparatus and the vacuole; controls entry of biosynthetic, endocytic, and retrograde traffic into the prevacuolar compartment.	++
RAV1	Subunit of RAVE complex (Rav1, Rav2, Skp1). The RAVE complex promotes assembly of the V-ATPase holoenzyme.	+++
RCY1	F-box protein involved in recycling endocytosed proteins.	++
RRT2	Methylesterase performing penultimate step of diphthamide biosynthesis; Rrt2is involved in endosomal recycling; forms complex with Rtt10p that functions in retromer-mediated pathway for recycling internalized cell-surface proteins.	++
RVS167	Calmodulin-binding actin-associated protein with roles in endocytic membrane tabulation and constriction, and exocytosis.	+
SEC28	Epsilon-COP subunit of the coatomer. Sec28 regulates retrograde Golgi-to-ER protein traffic.	++
SEC66	Non-essential subunit of Sec63 complex.	+++
SEC72	Non-essential subunit of Sec63 complex.	+++
SNF7	One of four subunits of the ESCRT-III complex. Snf1 is involved in the sorting of transmembrane proteins into the multivesicular body (MVB) pathway.	+++
SNF8	Component of the ESCRT-II complex.	+++

STP22	Component of the ESCRT-I complex.	++
SXM1	Nuclear transport factor (karyopherin) that is involved in protein transport between the cytoplasm and nucleoplasm.	+
SYO1	Transport adaptor or symportin that facilitates synchronized nuclear coimport of the two 5S-rRNA binding proteins Rpl5 and Rpl11. Syo1 is required for biogenesis of the large ribosomal subunit.	++
TIM18	Component of the mitochondrial TIM22 complex that is involved in insertion of polytopic proteins into the inner membrane.	+
TRS65	Component of transport protein particle (TRAPP) complex II; TRAPPII is a multimeric guanine nucleotide-exchange factor for the GTPase Ypt1, regulating intra-Golgi and endosome-Golgi traffic.	+++
VAM10	Protein involved in vacuole morphogenesis and acts at an early step of homotypic vacuole fusion that is required for vacuole tethering.	+
VAM3	Syntaxin-like vacuolar t-SNARE. Vam3 mediates docking/fusion of late transport intermediates with the vacuole.	+
VAM6	Guanine nucleotide exchange factor for the GTPase Gtr1. Vam6 is a Rab GTPase effector, interacting with both GTP- and GDP-bound conformations of Ypt7.	+
VAM7	Vacuolar SNARE protein; Vam7 functions with Vam3 in vacuolar protein trafficking.	+++
VPS1	Dynamin-like GTPase required for vacuolar sorting.	+++
VPS20	Myristoylated subunit of the ESCRT-III complex.	++
VPS21	Endosomal Rab family GTPase required for endocytic transport and sorting of vacuolar hydrolases. Vps21 is also required for endosomal localization of the CORVET complex.	++
VPS24	One of four subunits of the ESCRT-III comple. Vps24 is involved in the sorting of transmembrane proteins into the multivesicular body (MVB) pathway.	+++
VPS25	Component of the ESCRT-II complex.	+++
VPS27	Endosomal protein that forms a complex with Hse1.Vps27 is required for recycling Golgi proteins, forming lumenal membranes and sorting ubiquitinated proteins destined for degradation.	+++
VPS28	Component of the ESCRT-I complex.	++
VPS3	Component of CORVET membrane tethering complex. VPS is required for the sorting and processing of soluble vacuolar proteins, acidification of the vacuolar lumen, and assembly of the vacuolar H+-ATPase.	+++
VPS33	ATP-binding protein that is a subunit of the HOPS and CORVET complexes. Vps33 is essential for protein sorting, vesicle docking, and fusion at the vacuole.	+++
VPS36	Component of the ESCRT-II complex.	+++
VPS4	AAA-ATPase involved in multivesicular body (MVB) protein sorting.	++
VPS5	Nexin-1 homolog required for localizing membrane proteins from a prevacuolar/late endosomal compartment back to late Golgi.	+
VPS52	Component of the GARP (Golgi-associated retrograde protein) complex. GARP is required for the recycling of proteins from endosomes to the late Golgi, and for mitosis after DNA damage induced checkpoint arrest.	++
VPS64	Protein required for cytoplasm to vacuole targeting of proteins.	++
VPS72	Htz1-binding component of the SWR1 complex. Vps72 is required for vacuolar protein sorting.	+
VPS74	Golgi phosphatidylinositol-4-kinase effector and PtdIns4P sensor. Vps74 interacts with the cytosolic domains of cis and medial glycosyltransferases, and in the PtdIns4P-bound state mediates the	+

	targeting of these enzymes to the Golgi.	
YPT6	Rab family GTPase required for endosome-to-Golgii, intra-Golgi retrograde, and retrograde Golgi-to-ER transport.	+++

Ion homeostasis

Genes	Function	Susceptibility to methanol
FRE8	Protein with sequence similarity to iron/copper reductases; involved in iron homeostasis.	++

Lipid synthesis

Genes	Function	Susceptibility to methanol
ELO2	Fatty acid elongase, involved in sphingolipid biosynthesis.	+++
ELO3	Elongase involved in fatty acid and sphingolipid biosynthesis.	++
ERG2	C-8 sterol isomerase; catalyses isomerization of delta-8 double bond to delta-7 position at an intermediate step in ergosterol biosynthesis.	+
ERG3	C-5 sterol desaturase. Erg3 is glycoprotein that catalyses the introduction of a C-5(6) double bond into episterol, a precursor in ergosterol biosynthesis.	++
ISC1	Inositol phosphosphingolipid phospholipase C. Isc1 hydrolyzes complex sphingolipids to produce ceramide.	+
KCS1	Inositol hexakisphosphate and inositol heptakisphosphate kinase.	++
LIP5	Protein involved in biosynthesis of the coenzyme lipoic acid.	+
SAC1	Phosphatidylinositol phosphate (PtdInsP) phosphatase. Sac1 is involved in hydrolysis of PtdIns[4]P in the early and medial Golgi.	+
SPO7	Putative regulatory subunit of Nem1-Spo7 phosphatase holoenzyme. Spo7 regulates nuclear growth by controlling phospholipid biosynthesis.	++
TGL3	Bifunctional triacylglycerol lipase and LPE acyltransferase; major lipid particle-localized triacylglycerol (TAG) lipase; catalyses acylation of lysophosphatidylethanolamine (LPE), a function which is essential for sporulation.	+

Mitochondrial function

Genes	Function	Susceptibility to methanol
AIF1	Mitochondrial cell death effector.	+
ATP10	Mitochondrial inner membrane protein that is an assembly factor for the F0 sector of mitochondrial F1F0 ATP synthase	++
ATP11	Molecular chaperone that is required for the assembly of alpha and beta subunits into the F1 sector of mitochondrial F1F0 ATP synthase.	+
CEM1	Mitochondrial beta-keto-acyl synthase required for mitochondrial respiration.	++
COA3	Mitochondrial protein required for cytochrome c oxidase assembly.	+
COQ1	Hexaprenyl pyrophosphate synthetase. Coq1 catalyses the first step in ubiquinone (coenzyme Q) biosynthesis.	+
COQ10	Coenzyme Q (ubiquinone) binding protein that functions in the	+

	delivery of Q6 to its proper location for electron transport during respiration.	
COQ4	Protein with a role in ubiquinone (Coenzyme Q) biosynthesis.	+
COX17	Copper metallochaperone that transfers copper to Sco1 and Cox11.	+
COX18	Mitochondrial integral inner membrane protein required for membrane insertion of C-terminus of Cox2,	++
COX19	Protein required for cytochrome c oxidase assembly.	+
COX9	Subunit VIIa of cytochrome c oxidase (Complex IV).	++
GEP4	Mitochondrial phosphatidylglycerophosphatase (PGP phosphatase). Gep4 dephosphorylates phosphatidylglycerolphosphate to generate phosphatidylglycerol, an essential step during cardiolipin biosynthesis.	++
HFA1	Mitochondrial acetyl-coenzyme A carboxylase that catalyses the production of malonyl-CoA in mitochondrial fatty acid biosynthesis.	+
ICP55	Mitochondrial aminopeptidase. Icp55 cleaves the N termini of at least 38 imported proteins after cleavage by the mitochondrial processing peptidase (MPP), thereby increasing their stability.	++
IMP1	Catalytic subunit of mitochondrial inner membrane peptidase complex; required for maturation of mitochondrial proteins of the intermembrane space	+
ISA1	Protein required for maturation of mitochondrial [4Fe-4S] proteins.	+
MDM12	Mitochondrial outer membrane protein,required for transmission of mitochondria to daughter cells and for mitophagy.	+
MDM20	Non-catalytic subunit of the NatB N-terminal acetyltransferase. Mdm20 is involved in mitochondrial inheritance and actin assembly.	++
MEF1	Mitochondrial elongation factor involved in translational elongation.	+
MGM1	Mitochondrial GTPase required for mitochondrial morphology, fusion, and genome maintenance.	++
MIR1	Mitochondrial phosphate carrier that imports inorganic phosphate into mitochondria.	+++
MMR1	Phosphorylated protein of the mitochondrial outer membrane.	++
MRF1	Mitochondrial translation release factor that is involved in stop codon recognition and hydrolysis of the peptidyl-tRNA bond during mitochondrial translation.	+
MRM1	Ribose methyltransferase that modifies a functionally critical, conserved nucleotide in mitochondrial 21S rRNA	+
MRM2	Mitochondrial 2' O-ribose methyltransferase that is required for methylation of U(2791) in 21S rRNA.	+
MSE1	Mitochondrial glutamyl-tRNA synthetase.	+
MSY1	Mitochondrial tyrosyl-tRNA synthetase.	+
PET54	Mitochondrial inner membrane protein that binds to the 5' UTR of the COX3 mRNA to activate its translation together with Pet122 and Pet494.	+++
POR1	Mitochondrial porin (voltage-dependent anion channel) required for maintenance of mitochondrial osmotic stability and mitochondrial membrane permeability.	+
POS5	Mitochondrial NADH kinase that phosphorylates NADH.	+
PRX1	Mitochondrial peroxiredoxin with thioredoxin peroxidase activity. Prx1 has a role in reduction of hydroperoxides.	++
RMD9	Mitochondrial protein required for respiratory growth.	+
SHE9	Mitochondrial inner membrane protein required for normal mitochondrial morphology.	+

SHY1	Mitochondrial inner membrane protein required for complex IV assembly,	+
SLM5	Mitochondrial asparaginyl-tRNA synthetase.	+++
TIM13	Mitochondrial intermembrane space protein that forms a complex with Tim8 that delivers a subset of hydrophobic proteins to the TIM22 complex for insertion into the inner membrane.	+++
TIM8	Mitochondrial intermembrane space protein. Tim8 forms a complex with Tim13 that delivers a subset of hydrophobic proteins to the TIM22 complex for inner membrane insertion.	+
TOM6	Component of the TOM (translocase of outer membrane) complex. Tom6 is responsible for recognition and initial import steps for all mitochondrially directed proteins.	+
TOM70	Component of the TOM (translocase of outer membrane) complex that is involved in the recognition and initial import steps for all mitochondrially directed proteins.	++
TUF1	Mitochondrial translation elongation factor Tu (EF-Tu). Tuf1 is involved in fundamental pathway of mtDNA homeostasis.	+

Protein folding

Genes	Function	Susceptibility to methanol
BUD27	Unconventional prefoldin protein involved in translation initiation; required for correct assembly of RNAP I, II, and III in an Rpb5-dependent manner.	++
GIM3	Subunit of the heterohexameric cochaperone prefoldin complex; prefoldin binds specifically to cytosolic chaperonin and transfers target proteins to it.	+++
IRC25	Component of a heterodimeric Poc4-Irc25 chaperone. Irc25 is involved in assembly of alpha subunits into the 20S proteasome.	+
PAC10	Part of the heteromeric co-chaperone GimC/prefoldin complex - promotes efficient protein folding.	++
PIH1	Component of the conserved R2TP complex (Rvb1-Rvb2-Tah1-Pih1). The R2TP complex interacts with Hsp90 (Hsp82 and Hsc82) to mediate assembly large protein complexes such as box C/D snoRNPs and RNA polymerase II.	+++
SSE1	ATPase component of heat shock protein Hsp90 chaperone complex. Sse1 binds unfolded proteins.	+
SSZ1	Hsp70 protein that interacts with Zuo1. Ssz1 is also involved in pleiotropic drug resistance via sequential activation of *PDR1* and *PDR5*.	++
UMP1	Chaperone required for correct maturation of the 20S proteasome.	++
VPS75	NAP family histone chaperone. Vps75 binds to histones and Rtt109, stimulating histone acetyltransferase activity	++

Protein modification

Genes	Functions	Susceptibility to methanol
AKR1	Palmitoyl transferase involved in protein palmitoylation.	+++
ALG3	Dolichol-P-Man dependent alpha (1-3) mannosyltransferase that is involved in synthesis of dolichol-linked oligosaccharide donor for N-linked glycosylation of proteins.	++

ALG5	UDP-glucose:dolichyl-phosphate glucosyltransferase that is involved in asparagine-linked glycosylation in the endoplasmic reticulum.	++
ALG6	Alpha 1,3 glucosyltransferase that is involved in transfer of oligosaccharides from dolichyl pyrophosphate to asparagine residues of proteins during N-linked protein glycosylation.	+
ALG8	Glucosyl transferase that is involved in N-linked glycosylation.	++
ARG82	Inositol polyphosphate multikinase (IPMK); sequentially phosphorylates Ins(1,4,5)P3 to form Ins(1,3,4,5,6)P5.	++
CKB2	Beta' regulatory subunit of casein kinase 2 (CK2). Ckb2 is a Ser/Thr protein kinase with roles in cell growth and proliferation.	+
DUF1	Ubiquitin-binding protein of unknown function.	+
MCK1	Dual-specificity Ser/Thr and tyrosine protein kinase. Mck1 participates in chromosome segregation, meiotic entry, genome stability.	+
MTQ2	Adenosylmethionine-dependent methyltransferase.	+
NAT1	Subunit of protein N-terminal acetyltransferase NatA.	++
OST6	Subunit of the oligosaccharyltransferase complex of the ER lumen complex. This complex catalyses asparagine-linked glycosylation of newly synthesized proteins.	+
PER1	Protein of the endoplasmic reticulum required for GPI-phospholipase A2 activity that remodels the GPI anchor as a prerequisite for association of GPI-anchored proteins with lipid rafts.	++
PPT2	Phosphopantetheine:protein transferase (PPTase). Ppt2 activates mitochondrial acyl carrier protein (Acp1).	++
SAP155	Protein required for function of the Sit4 protein phosphatase.	++
SEM1	19S proteasome regulatory particle lid subcomplex component.	++
SLX8	Subunit of Slx5-Slx8 SUMO-targeted ubiquitin ligase (STUbL) complex. Slx plays a role in proteolysis of spindle positioning protein Kar9, DNA repair proteins Rad52 and Rad57.	++
UFO1	F-box receptor protein; subunit of the Skp1-Cdc53-F-box receptor (SCF) E3 ubiquitin ligase complex.	++
VID22	Glycosylated integral membrane protein that plays a role in fructose-1,6-bisphosphatase degradation.	++
YME1	Catalytic subunit of i-AAA protease complex. Yme1 is responsible for degradation of unfolded or misfolded mitochondrial gene products.	++

Protein synthesis

Genes	Function	Susceptibility to methanol
DOM34	Protein that facilitates ribosomal subunit dissociation.	+
GEP3	Protein required for mitochondrial ribosome small subunit biogenesis.	+
IMG2	Mitochondrial ribosomal protein of the large subunit.	+++
MRP7	Mitochondrial ribosomal protein of the large subunit.	++
MRPL10	Mitochondrial ribosomal protein of the large subunit.	+
MRPL17	Mitochondrial ribosomal protein of the large subunit.	+
MRPL20	Mitochondrial ribosomal protein of the large subunit.	++
MRPL22	Mitochondrial ribosomal protein of the large subunit.	+
MRPL24	Mitochondrial ribosomal protein of the large subunit.	+

MRPL25	Mitochondrial ribosomal protein of the large subunit.	+
MRPL27	Mitochondrial ribosomal protein of the large subunit.	+
MRPL7	Mitochondrial ribosomal protein of the large subunit.	++
MRPS28	Mitochondrial ribosomal protein of the small subunit.	+
MRPS35	Mitochondrial ribosomal protein of the small subunit.	++
RPL12B	Ribosomal 60S subunit protein L12B.	+
RPL14A	Ribosomal 60S subunit protein L14A	++
RPL16B	Ribosomal 60S subunit protein L16B.	+
RPL19B	Ribosomal 60S subunit protein L19B.	++
RPL21A	Ribosomal 60S subunit protein L21A.	++
RPL36A	Ribosomal 60S subunit protein L36A.	+++
RPL43A	Ribosomal 60S subunit protein L43A.	++
RPL8A	Ribosomal 60S subunit protein L8A.	+
RPS0B	Protein component of the small (40S) ribosomal subunit.	++
RPS11A	Protein component of the small (40S) ribosomal subunit.	+
RPS17A	Ribosomal protein 51 (rp51) of the small (40s) subunit.	+
RPS19A	Protein component of the small (40S) ribosomal subunit.	+
RPS22B	Protein component of the small (40S) ribosomal subunit.	++
RPS24A	Protein component of the small (40S) ribosomal subunit.	+
RPS27B	Protein component of the small (40S) ribosomal subunit.	+
RSM18	Mitochondrial ribosomal protein of the small subunit.	+
RSM23	Mitochondrial ribosomal protein of the small subunit.	+
RSM7	Mitochondrial ribosomal protein of the small subunit.	+++
TEF4	Gamma subunit of translational elongation factor eEF1B.	+++
IFM1	Mitochondrial translation initiation factor 2.	++

Response to stress

Genes	Function	Susceptibility to methanol
GSH1	Gamma glutamylcysteine synthetase. Gsh1 catalyses the first step in glutathione (GSH) biosynthesis.	++
MXR1	Methionine-S-sulfoxide reductase that is involved in the response to oxidative stress, Mxr1 also protects iron-sulfur clusters from oxidative inactivation along with *MXR2*.	+
RMD8	Cytosolic protein required for sporulation.	++
SOD1	Cytosolic copper-zinc superoxide dismutase; detoxifies superoxide.	+++

Transcription factors

Gene	Function	Susceptibility to methanol
CBF1	Transcription factor that associates with kinetochore proteins, required for chromosome segregation. Basic helix-loop-helix (bHLH) protein; forms homodimer to bind E-box consensus sequence CACGTG present at MET gene promoters and centromere DNA element I (CDEI); affects nucleosome positioning at this motif; associates with other transcription factors such as Met4 and Isw1 to mediate transcriptional activation or repression;	+
GLN3	Transcriptional activator of genes regulated by nitrogen catabolite repression; localization and activity regulated by quality of nitrogen source and Ure2.	+
IXR1	Transcriptional repressor that regulates hypoxic genes during	+++

	normoxia; involved in the aerobic repression of genes such as *COX5*b, *TIR1*, and *HEM13*.	
NGG1	Transcriptional regulator involved in glucose repression of Gal4-regulated genes. Subunit of chromatin modifying histone acetyltransferase complexes; member of the ADA complex, the SAGA complex, and the SLIK complex.	++
OPI1	Transcriptional regulator of a variety of genes; its phosphorylation by protein kinase A stimulates Opi1 function in negative regulation of phospholipid biosynthetic genes; involved in telomere maintenance.	+++
RPH1	Transcription factor with JmjC domain-containing histone demethylase. Rph1 targets tri- and dimethylated H3K36 and associates with actively transcribed regions and promotes elongation. Rph1 acts as a repressor of autophagy-related genes in nutrient-replete conditions.	+
RPN4	Transcription factor that stimulates expression of proteasome encoding genes being regulated by the 26S proteasome in a negative feedback control mechanism; *RPN4* is transcriptionally regulated by various stress response.	+
SFL1	Transcriptional repressor and activator; involved in repression of flocculation-related genes, and activation of stress responsive genes; has direct role in *INO1* transcriptional memory; negatively regulated by cAMP-dependent protein kinase A subunit Tpk2.	++
SOK2	Transcription factor that negatively regulates pseudohyphal differentiation. Sok2 also plays a regulatory role in the cyclic AMP (cAMP)-dependent protein kinase (PKA) signal transduction pathway.	++
SFP1	Transcription factor that regulates ribosomal protein and biogenesis genes. Sfp1 also regulates response to nutrients and stress, G2/M transitions during mitotic cell cycle and DNA-damage response and modulates cell size.	++
STB5	Transcription factor involved in the regulation multidrug resistance and oxidative stress response; forms a heterodimer with Pdr1; contains a Zn(II)2Cys6 zinc finger domain that interacts with a pleiotropic drug resistance element *in vitro*.	++
UME6	Transcriptional regulator of early meiotic genes; involved in chromatin remodelling and transcriptional repression via DNA looping; binds URS1 upstream regulatory sequence, represses transcription by recruiting conserved histone deacetylase Rpd3 (through co-repressor Sin3) and chromatin-remodelling factor Isw2; couples metabolic responses to nutritional cues with initiation and progression of meiosis.	++

Transporters

Gene	Function	Susceptibility to methanol
AGP2	General amino acid permease with broad substrate specificity. Can also transport carnitine.	++
FEN2	Plasma membrane H+-pantothenate symporter.	+++
MCH4	Probable transporter. Does not act in the transport of monocarboxylic acids across the plasma membrane.	+
MCH5	Riboflavin transporter involved in riboflavin (vitamin B2) uptake. monocarboxylic acids across the plasma membrane.	+++
MUP1	High affinity methionine permease that is also involved in cysteine	++

	uptake.	
PET8	S-adenosyl-L-methionine transport.	+
SPF1	Mediates manganese transport into the endoplasmic reticulum. The ATPase activity is required for cellular manganese homeostasis.	+++
TPN1	Thiamine-regulated, high affinity import carrier of pyridoxine, pyridoxal and pyridoxamine.	++
VMR1	ATP-binding cassette (ABC) family member involved in multiple drug resistance and metal sensitivity.	+

Unknown function

Gene	Function	Susceptibility to methanol
AIM22	Putative lipoate-protein ligase.	++
AIM26	Protein of unknown function.	+++
DIA1	Protein of unknown function; involved in invasive and pseudohyphal growth.	++
EMI1	Non-essential protein of unknown function; required for transcriptional induction of the early meiotic-specific transcription factor IME1, also required for sporulation.	+
ERD1	Predicted membrane protein required for lumenal ER protein retention.	+
FSH3	Putative serine hydrolase.	+
FYV1	Dubious open reading frame; unlikely to encode a functional protein.	+
ILM1	Protein of unknown function; may be involved in mitochondrial DNA maintenance; required for slowed DNA synthesis-induced filamentous growth.	+++
IRC13	Putative protein of unknown function.	+
MCT1	Predicted malonyl-CoA:ACP transferase.	+
NRP1	Putative RNA binding protein of unknown function.	++
OPI8	Dubious open reading frame; unlikely to encode a functional protein,	++
OPI9	Dubious open reading frame; unlikely to encode a functional protein	+++
RBK1	Putative ribokinase.	++
RRG9	Protein of unknown function.	+
SWS2	Putative mitochondrial ribosomal protein of the small subunit.	+
TAN1	Putative tRNA acetyltransferase;	+++
VPS61	Dubious open reading frame; unlikely to encode a functional protein.	**
VPS63	Putative protein of unknown function.	+++
VPS65	Dubious open reading frame; unlikely to encode a functional protein.	+++
YBL083C	Dubious open reading frame; unlikely to encode a functional protein.	++
YBL094C	Dubious open reading frame; unlikely to encode a functional protein.	+++
YBR085C-A	Protein of unknown function.	+
YBR225W	Putative protein of unknown function.	+
YBR226C	Dubious open reading frame; unlikely to encode a functional protein.	++
YCR025C	Putative protein of unknown function.	++
YDL062W	Dubious open reading frame; unlikely to encode a functional	++

	protein.	
YDL199C	Putative transporter; member of the sugar porter family.	++
YDR008C	Dubious open reading frame; unlikely to encode a functional protein.	++
YDR203W	Dubious open reading frame; unlikely to encode a functional protein.	++
YDR230W	Dubious open reading frame; unlikely to encode a functional protein.	++
YDR417C	Dubious open reading frame; unlikely to encode a functional protein.	+
YDR431W	Dubious open reading frame; unlikely to encode a functional protein.	+
YER156C	Putative protein of unknown function.	+
YGL007C-A	Putative protein of unknown function	+++
YGL149W	Putative protein of unknown function.	+
YGL235W	Putative protein of unknown function.	++
YGR011W	Dubious open reading frame; unlikely to encode a functional protein.	++
YGR064W	Dubious open reading frame; unlikely to encode a functional protein.	++
YGR182C	Dubious open reading frame; unlikely to encode a functional protein.	+++
YHL005C	Putative protein of unknown function.	+
YIM2	Dubious open reading frame; unlikely to encode a functional protein.	+
YJL120W	Dubious open reading frame; unlikely to encode a functional protein.	++
YJR011C	Putative protein of unknown function.	++
YJR120W	Protein of unknown function.	+
YLR111W	Dubious open reading frame; unlikely to encode a functional protein.	++
YLR202C	Dubious open reading frame; unlikely to encode a functional protein.	++
YLR235C	Dubious open reading frame; unlikely to encode a functional protein,	++
YLR269C	Dubious open reading frame; unlikely to encode a functional protein.	+++
YLR366W	Dubious open reading frame; unlikely to encode a functional protein.	+
YML009W-B	Dubious open reading frame; unlikely to encode a functional protein.	++
YML012C-A	Dubious open reading frame; unlikely to encode a functional protein.	++
YMR031W-A	Dubious open reading frame; unlikely to encode a functional protein,	+++
YMR075C-A	Dubious open reading frame; unlikely to encode a functional protein.	++
YNL140C	Protein of unknown function.	+
YNL165W	Putative protein of unknown function.	+
YNL319W	Dubious open reading frame; unlikely to encode a functional protein.	++
YNR005C	Dubious open reading frame; unlikely to encode a functional protein.	++
YOR139C	Dubious open reading frame; unlikely to encode a functional protein.	++
YOR199W	Dubious open reading frame; unlikely to encode a functional	+

	protein.	
YOR200W	Dubious open reading frame; unlikely to encode a functional protein.	+
YOR331C	Dubious open reading frame; unlikely to encode a functional protein.	+

Table S2. List of genes whose expression increases *S. cerevisiae* tolerance to 5.5%(v/v) ethanol based on the screening of the Euroscarf deletion mutant collection; the elimination of the indicated genes increases yeast susceptibility to ethanol. Biological functions are based on the information available in SGD (www.yeastgenome.org). The symbols '+++', '++' and '+' correspond to phenotypes as shown in Figure S1.

Autophagy

Gene	Function	Susceptibility level
ATG10	Conserved E2-like conjugating enzyme; mediates formation of the Atg12-Atg5 conjugate, which is a critical step in autophagy.	+
ATG11	Adapter protein for pexophagy and the Cvt targeting pathway; directs receptor-bound cargo to the phagophore assembly site (PAS) for packaging into vesicles; required for recruiting other proteins to the PAS; recruits Dnm1p to facilitate fission of mitochondria that are destined for removal by mitophagy.	++
ATG17	Scaffold protein responsible for phagophore assembly site organization; regulatory subunit of an autophagy-specific complex that includes Atg1 and Atg13; stimulates Atg1 kinase activity.	+
ATG2	Peripheral membrane protein required for autophagic vesicle formation; also required for vesicle formation during pexophagy and the cytoplasm-to-vaucole targeting (Cvt) pathway; involved in Atg9p cycling between the phagophore assembly site and mitochondria; contains an APT1 domain that binds phosphatidylinositol-3-phosphate; essential for cell cycle progression from G2/M to G1 under nitrogen starvation; forms cytoplasmic foci upon DNA replication stress.	+
ATG20	Sorting nexin family member; required for the cytoplasm-to-vacuole targeting (Cvt) pathway and for endosomal sorting; has a Phox homology domain that binds phosphatidylinositol-3-phosphate; interacts with Snx4; potential Cdc28 substrate.	+++
ATG4	Conserved cysteine protease required for autophagy; cleaves Atg8p to a form required for autophagosome and Cvt vesicle generation.	+
MEH1	Component of the EGO and GSE complexes; EGO is involved in the regulation of microautophagy and GSE is required for proper sorting of amino acid permease Gap1.	+

Carbohydrate metabolism

Gene	Function	Susceptibility level
ADH3	Mitochondrial alcohol dehydrogenase isozyme III; involved in the shuttling of mitochondrial NADH to the cytosol under anaerobic conditions and ethanol production.	++

ARO1	Pentafunctional arom protein; catalyzes steps 2 through 6 in the biosynthesis of chorismate, which is a precursor to aromatic amino acids.	+
ARO2	Bifunctional chorismate synthase and flavin reductase; catalyzes the conversion of 5-enolpyruvylshikimate 3-phosphate (EPSP) to form chorismate, which is a precursor to aromatic amino acids; protein abundance increases in response to DNA replication stress.	++
ARO7	Chorismate mutase; catalyzes the conversion of chorismate to prephenate to initiate the tyrosine/phenylalanine-specific branch of aromatic amino acid biosynthesis.	+++
BNA1	3-hydroxyanthranilic acid dioxygenase; required for the *de novo* biosynthesis of NAD from tryptophan via kynurenine; expression regulated by Hst1.	++
FUM1	Fumarase; converts fumaric acid to L-malic acid in the TCA cycle; cytosolic and mitochondrial distribution determined by the N-terminal targeting sequence, protein conformation, and status of glyoxylate shunt; phosphorylated in mitochondria.	++
GCN5	Catalytic subunit of ADA and SAGA histone acetyltransferase complexes; modifies N-terminal lysines on histones H2B and H3; acetylates Rsc4p, a subunit of the RSC chromatin-remodeling complex, altering replication stress tolerance; relocalizes to the cytosol in response to hypoxia; mutant displays reduced transcription elongation in the G-less-based run-on (GLRO).	++
GPH1	Glycogen phosphorylase required for the mobilization of glycogen; non-essential; regulated by cyclic AMP-mediated phosphorylation; phosphorylation by Cdc28 may coordinately regulate carbohydrate metabolism and the cell cycle; expression is regulated by stress-response elements and by the HOG MAP kinase pathway.	++
KGD2	Dihydrolipoyl transsuccinylase; component of the mitochondrial alpha-ketoglutarate dehydrogenase complex, which catalyzes the oxidative decarboxylation of alpha-ketoglutarate to succinyl-CoA in the TCA cycle.	+
MET7	Folylpolyglutamate synthetase; catalyzes extension of the glutamate chains of the folate coenzymes, required for methionine synthesis and for maintenance of mitochondrial DNA.	+
PCL7	Pho85p cyclin of the Pho80 subfamily; forms a functional kinase complex with Pho85 which phosphorylates Mmr1 and is regulated by Pho81; involved in glycogen metabolism, expression is cell-cycle regulated; *PCL7* has a paralog, *PCL6*, that arose from the whole genome duplication.	+
PDC1	Major of three pyruvate decarboxylase isozymes; key enzyme in alcoholic fermentation; decarboxylates pyruvate to acetaldehyde; involved in amino acid catabolism.	+
PRS3	5-phospho-ribosyl-1(alpha)-pyrophosphate synthetase; synthesizes PRPP, which is required for nucleotide, histidine, and tryptophan biosynthesis; one of five related enzymes, which are active as heteromultimeric complexes.	+++
REG1	Regulatory subunit of type 1 protein phosphatase Glc7; involved in negative regulation of glucose-repressible genes; involved in regulation of the nucleocytoplasmic shuttling of Hxk2; *REG1* has a paralog, *REG2*, that arose from the whole genome duplication.	+++

REG2	Regulatory subunit of the Glc7 type-1 protein phosphatase; involved with Reg1, Glc7, and Snf1 in regulation of glucose-repressible genes.	+
RIB4	Lumazine synthase (DMRL synthase); catalyzes synthesis of immediate precursor to riboflavin; DMRL synthase stands for 6,7-dimethyl-8-ribityllumazine synthase.	+++

Cell cycle

Gene	Function	Susceptibility level
CIK1	Kinesin-associated protein; required for both karyogamy and mitotic spindle organization, interacts stably and specifically with Kar3 and may function to target this kinesin to a specific cellular role; locus encodes a long and short transcript with differing functions; *CIK1* has a paralog, *VIK1*, that arose from the whole genome duplication.	+++
CLN1	G1 cyclin involved in regulation of the cell cycle; activates Cdc28 kinase to promote the G1 to S phase transition; late G1 specific expression depends on transcription factor complexes, MBF (Swi-Mbp1) and SBF (Swi6-Swi4).	+
HOS4	Subunit of the Set3 complex; complex is a meiotic-specific repressor of sporulation specific genes that contains deacetylase activity.	+
IRC19	Protein of unknown function; *YLL033W* is not an essential gene but mutant is defective in spore formation; null mutant displays increased levels of spontaneous Rad52 *foc*i.	++
SPO22	Meiosis-specific protein essential for chromosome synapsis; involved in completion of nuclear divisions during meiosis.	+

Cell wall function

Gene	Function	Susceptibility level
CHS1	Chitin synthase I; requires activation from zymogenic form in order to catalyze the transfer of N-acetylglucosamine (GlcNAc) to chitin; required for repairing the chitin septum during cytokinesis; transcription activated by mating factor.	+++
CWH43	GPI lipid remodelase; responsible for introducing ceramides into GPI anchors having a C26:0 fatty acid in sn-2 of the glycerol moiety; can also use lyso-GPI protein anchors and various base resistant lipids as substrates.	+
FKS1	Catalytic subunit of 1,3-beta-D-glucan synthase; functionally redundant with alternate catalytic subunit Gsc2; binds to regulatory subunit Rho1p; involved in cell wall synthesis and maintenance; localizes to sites of cell wall remodeling.	+++
MNN11	Subunit of a Golgi mannosyltransferase complex; this complex also contains Anp1, Mnn9, Mnn10, and Hoc1, and mediates elongation of the polysaccharide mannan backbone; has homology to Mnn10.	+++

PUN1	Plasma membrane protein with a role in cell wall integrity; co-localizes with Sur7 in punctate membrane patches.	+
ROM2	Guanine nucleotide exchange factor (GEF) for Rho1 and Rho2; mutations are synthetically lethal with mutations in *rom1*, which also encodes a GEF; Rom2 localization to the bud surface is dependent on Ack1p; *ROM2* has a paralog, *ROM1*, that arose from the whole genome duplication.	+
ROT2	Glucosidase II catalytic subunit; required to trim the final glucose in N-linked glycans; required for normal cell wall synthesis; mutations in rot2 suppress *tor2* mutations, and are synthetically lethal with *rot1* mutations.	++
SAG1	Alpha-agglutinin of alpha-cells; binds to Aga1 during agglutination, N-terminal half is homologous to the immunoglobulin superfamily and contains binding site for a-agglutinin, C-terminal half is highly glycosylated and contains GPI anchor.	++
SLG1	Sensor-transducer of the stress-activated PKC1-MPK1 kinase pathway; involved in maintenance of cell wall integrity; required for mitophagy; involved in organization of the actin cytoskeleton; secretory pathway Wsc1 is required for the arrest of secretion response.	+++
SMI1	Protein involved in the regulation of cell wall synthesis; proposed to be involved in coordinating cell cycle progression with cell wall integrity.	++
YEH2	Steryl ester hydrolase; catalyzes steryl ester hydrolysis at the plasma membrane; involved in sterol metabolism; *YEH2* has a paralog, *YEH1*, that arose from the whole genome duplication.	++

Chromatin remodelling and transcriptional control

Gene	Function	Susceptibility level
ADA2	Transcription coactivator; component of the ADA and SAGA transcriptional adaptor/HAT (histone acetyltransferase) complexes.	++
APA1	AP4A phosphorylase; catalyzes phosphorolysis of dinucleoside oligophosphates, cleaving substrates' alpha/beta-anhydride bond and introducing Pi into the beta-position of the corresponding NDP formed.	+
ADO1	Adenosine kinase; required for the utilization of S-adenosylmethionine (AdoMet); may be involved in recycling adenosine produced through the methyl cycle.	+++
APT1	Adenine phosphoribosyltransferase; catalyzes the formation of AMP from adenine and 5-phosphoribosylpyrophosphate; involved in the salvage pathway of purine nucleotide biosynthesis; *APT1* has a paralog, *APT2*, that arose from the whole genome duplication.	++
BUD13	Subunit of the RES complex; RES complex is required for nuclear pre-mRNA retention and splicing; involved in bud-site selection; diploid mutants display a unipolar budding pattern instead of the wild-type bipolar pattern due to a specific defect in MATa1 pre-mRNA splicing which leads to haploid gene expression in diploids.	+++

CBP2	Required for splicing of the group I intron bI5 of the COB pre-mRNA; nuclear-encoded mitochondrial protein that binds to the RNA to promote splicing; also involved in but not essential for splicing of the COB bI2 intron and the intron in the 21S rRNA gene.	++
CBS1	Mitochondrial translational activator of the COB mRNA; membrane protein that interacts with translating ribosomes, acts on the COB mRNA 5'-untranslated leader.	+
CBS2	Mitochondrial translational activator of the COB mRNA; interacts with translating ribosomes, acts on the COB mRNA 5'-untranslated leader.	++
CCR4	Component of the *CCR4-NOT* transcriptional complex; *CCR4-NOT* is involved in regulation of gene expression; component of the major cytoplasmic deadenylase, which is involved in mRNA poly(A) tail shortening.	+
CDC50	Endosomal protein that interacts with phospholipid flippase Drs2; interaction with Cdc50 is essential for Drs2 catalytic activity; mutations affect cell polarity and polarized growth; similar to Lem3; *CDC50* has a paralog, *YNR048W*, that arose from the whole genome duplication.	+++
COQ4	Protein with a role in ubiquinone (Coenzyme Q) biosynthesis; possibly functioning in stabilization of Coq7; located on matrix face of mitochondrial inner membrane.	+
COQ10	Coenzyme Q (ubiquinone) binding protein; functions in the delivery of Q6 to its proper location for electron transport during respiration; START domain protein with homologs in bacteria and eukaryotes; respiratory growth defect of the null mutant is functionally complemented by human *COQ10A*.	++
CSE2	Subunit of the RNA polymerase II mediator complex; associates with core polymerase subunits to form the RNA polymerase II holoenzyme; component of the Middle domain of mediator; required for regulation of RNA polymerase II activity; relocalizes to the cytosol in response to hypoxia.	++
DEP1	Component of the Rpd3L histone deacetylase complex; required for diauxic shift-induced histone H2B deposition onto rDNA genes; transcriptional modulator involved in regulation of structural phospholipid biosynthesis genes and metabolically unrelated genes, as well as maintenance of telomeres, mating efficiency, and sporulation.	+
DHH1	Cytoplasmic DEAD-box helicase, stimulates mRNA decapping; coordinates distinct steps in mRNA function and decay, interacting with both decapping and deadenylase complexes; role in translational repression, mRNA decay, and possibly mRNA export; interacts and cooperates with Ngr1 to promote specific mRNA decay; ATP- and RNA-bound form promotes processing body (PB) assembly, while ATPase stimulation by Not1p promotes PB disassembly; forms cytoplasmic *foci* on replication stress.	+++
DST1	General transcription elongation factor TFIIS; enables RNA polymerase II to read through blocks to elongation by stimulating cleavage of nascent transcripts stalled at transcription arrest sites; maintains RNAPII elongation activity on ribosomal protein genes during conditions of transcriptional stress.	+++

ELP3	Subunit of Elongator complex; Elongator is required for modification of wobble nucleosides in tRNA; exhibits histone acetyltransferase activity that is directed to histones H3 and H4; disruption confers resistance to *K. lactis* zymotoxin; human homolog *ELP3* can partially complement yeast *elp3* null mutant.	++
GTF1	Subunit of the trimeric GatFAB AmidoTransferase(AdT) complex; involved in the formation of Q-tRNAQ; transposon insertion mutant is salt sensitive and null mutant has growth defects; non-tagged protein is detected in purified mitochondria.	++
HDA2	Subunit of the HDA1 histone deacetylase complex; possibly tetrameric trichostatin A-sensitive class II histone deacetylase complex contains Hda1p homodimer and an Hda2-Hda3 heterodimer; involved in telomere maintenance; relocalizes to the cytosol in response to hypoxia.	++
HIT1	Protein involved in C/D snoRNP assembly; regulates abundance of Rsa1p; required for growth at high temperature; similar to human *ZNHI*T.	++
HMI1	Mitochondrial inner membrane localized ATP-dependent DNA helicase; required for the maintenance of the mitochondrial genome; not required for mitochondrial transcription; has homology to *E. coli* helicase *uvrD*.	++
HMO1	Chromatin associated high mobility group (HMG) family member; involved in compacting, bending, bridging and looping DNA; rDNA-binding component that regulates transcription from RNA polymerase I promoters; regulates start site selection of ribosomal protein genes via RNA polymerase II promoters.	+++
HPR1	Subunit of *THO/TREX* complexes; this complex couple transcription elongation with mitotic recombination and with mRNA metabolism and export, subunit of an RNA Pol II complex; regulates lifespan; involved in telomere maintenance; similar to Top1.	+++
HTL1	Component of the RSC chromatin remodeling complex; RSC functions in transcriptional regulation and elongation, chromosome stability, and establishing sister chromatid cohesion; involved in telomere maintenance.	+++
HTZ1	Histone variant H2AZ; exchanged for histone H2A in nucleosomes by the SWR1 complex; involved in transcriptional regulation through prevention of the spread of silent heterochromatin; Htz1-containing nucleosomes facilitate RNA Pol II passage by affecting correct assembly and modification status of RNA Pol II elongation complexes and by favoring efficient nucleosome remodeling.	+++
IME4	mRNA N6-adenosine methyltransferase required for entry into meiosis; mediates N6-adenosine methylation of bulk mRNA during the induction of sporulation which includes the meiotic regulators *IME1, IME2* and *IME4* itself; repressed in haploids via production of antisense *IME4* transcripts.	+
IST3	Component of the U2 snRNP; required for the first catalytic step of splicing and for spliceosomal assembly; interacts with Rds3 and is required for Mer1p-activated splicing; diploid mutants have a specific defect in MATa1 pre-mRNA splicing which leads to haploid gene expression in diploids.	+
LEA1	Component of U2 snRNP complex; disruption causes reduced U2 snRNP levels; physically interacts with Msl1p; putative homolog of human U2A' snRNP protein.	+
LDB7	Component of the RSC chromatin remodeling complex; interacts	+

	with Rsc3, Rsc30, Npl6, and Htl1 to form a module important for a broad range of RSC functions.	
MAF1	Highly conserved negative regulator of RNA polymerase III; involved in tRNA processing and stability; inhibits tRNA degradation via rapid tRNA decay (RTD) pathway; binds N-terminal domain of Rpc160 subunit of Pol III to prevent closed-complex formation; regulated by phosphorylation mediated by *TORC1*, protein kinase A, Sch9, casein kinase 2; localizes to cytoplasm during vegetative growth and translocates to nucleus and nucleolus under stress conditions.	+++
MED1	Subunit of the RNA polymerase II mediator complex; associates with core polymerase subunits to form the RNA polymerase II holoenzyme; essential for transcriptional regulation.	++
MFT1	Subunit of the THO complex; *THO* is a nuclear complex comprised of Hpr1, Mft1, Rlr1, and Thp2, that is involved in transcription elongation and mitotic recombination; involved in telomere maintenance.	+
MRF1	Mitochondrial translation release factor; involved in stop codon recognition and hydrolysis of the peptidyl-tRNA bond during mitochondrial translation; lack of *MRF1* causes mitochondrial genome instability.	+++
MSE1	Mitochondrial glutamyl-tRNA synthetase; predicted to be palmitoylated.	++
NPT1	Nicotinate phosphoribosyltransferase; acts in the salvage pathway of NAD+ biosynthesis; required for silencing at rDNA and telomeres and has a role in silencing at mating-type *loci*.	+
NPL3	RNA-binding protein; promotes elongation, regulates termination, and carries poly(A) mRNA from nucleus to cytoplasm; represses translation initiation by binding eIF4G; required for pre-mRNA splicing; interacts with E3 ubiquitin ligase Bre1, linking histone ubiquitination to mRNA processing; may have role in telomere maintenance; dissociation from mRNAs promoted by Mtr10; phosphorylated by Sky1 in cytoplasm; protein abundance increases in response to DNA replication stress.	++
NRP1	Putative RNA binding protein of unknown function; localizes to stress granules induced by glucose deprivation; predicted to be involved in ribosome biogenesis.	++
PHO23	Component of the Rpd3L histone deacetylase complex; involved in transcriptional regulation of *PHO5*; affects termination of snoRNAs and cryptic unstable transcripts (CUTs).	+
POP2	RNase of the DEDD superfamily; subunit of the Ccr4-Not complex that mediates 3' to 5' mRNA deadenylation.	++
REF2	RNA-binding protein; involved in the cleavage step of mRNA 3'-end formation prior to polyadenylation, and in snoRNA maturation; part of holo-CPF subcomplex APT, which associates with 3'-ends of snoRNA- and mRNA-encoding genes; putative regulatory subunit of type 1 protein phosphatase Glc7p, required for actomyosin ring formation, and for timely dephosphorylation and release of Bnr1p from the division site; relocalizes to the cytosol in response to hypoxia.	++
RXT2	Component of the histone deacetylase Rpd3L complex; possibly involved in cell fusion and invasive growth.	+
RXT3	Component of the Rpd3L histone deacetylase complex; involved in histone deacetylation.	+

RMI1	Subunit of the RecQ (Sgs1) - Topo III (Top3p) complex; stimulates superhelical relaxing, DNA catenation/decatenation and ssDNA binding activities of Top3; involved in response to DNA damage; functions in S phase-mediated cohesion establishment via a pathway involving the Ctf18-RFC complex and Mrc1; stimulates Top3 DNA catenation/decatenation activity; null mutants display increased rates of recombination and delayed S phase.	++
RPB9	RNA polymerase II subunit B12.6; contacts DNA; mutations affect transcription start site selection and fidelity of transcription.	+++
RRN10	Protein involved in promoting high level transcription of rDNA; subunit of UAF (upstream activation factor) for RNA polymerase I.	++
RRP6	Nuclear exosome exonuclease component; has 3'-5' exonuclease activity that is regulated by Lrp1; involved in RNA processing, maturation, surveillance, degradation, tethering, and export; role in sn/snoRNAs precursor degradation; forms a stable heterodimer with Lrp1; has similarity to *E. coli* RNase D and to human PM-*Sc1 100 (EXOSC10*).	++
RSC2	Component of the RSC chromatin remodeling complex; required for expression of mid-late sporulation-specific genes; involved in telomere maintenance; *RSC2* has a paralog, *RSC1*, that arose from the whole genome duplication.	+++
SHE2	RNA-binding protein that binds specific mRNAs and interacts with She3; part of the mRNA localization machinery that restricts accumulation of certain proteins to the bud.	+
SDS23	Protein involved in cell separation during budding; one of two *S. cerevisiae* homologs (Sds23p and Sds24p) of the S. pombe Sds23 protein, which is implicated in APC/cyclosome regulation; *SDS23* has a paralog, *SDS24*, that arose from the whole genome duplication.	+++
SGS1	RecQ family nucleolar DNA helicase; role in genome integrity maintenance, chromosome synapsis, meiotic joint molecule/crossover formation; stimulates activity of Top3; rapidly lost in response to rapamycin in Rrd1p-dependent manner; forms nuclear foci upon DNA replication stress; yeast *SGS1* complements mutations in human homolog BLM implicated in Bloom syndrome; also similar to human WRN implicated in Werner syndrome; human BLM and WRN can each complement yeast null mutant.	++
SIN3	Component of both the Rpd3S and Rpd3L histone deacetylase complexes; involved in transcriptional repression and activation of diverse processes, including mating-type switching and meiosis; involved in the maintenance of chromosomal integrity.	+
SRB2	Subunit of the RNA polymerase II mediator complex; associates with core polymerase subunits to form the RNA polymerase II holoenzyme; general transcription factor involved in telomere maintenance.	+++
SRB8	Subunit of the RNA polymerase II mediator complex; associates with core polymerase subunits to form the RNA polymerase II holoenzyme; essential for transcriptional regulation; involved in glucose repression.	++

SSN2	Subunit of the RNA polymerase II mediator complex; associates with core polymerase subunits to form the RNA polymerase II holoenzyme; required for stable association of Srb10-Srb11 kinase; essential for transcriptional regulation.	+++
SSN8	Cyclin-like component of the RNA polymerase II holoenzyme; involved in phosphorylation of the RNA polymerase II C-terminal domain; forms a kinase-cyclin pair in the RNAPII holoenzyme with Ssn3p; required for both entry into and execution of the meiotic program; involved in glucose repression and telomere maintenance; cyclin homolog 35% identical to human cyclin C.	+++
SSO2	Plasma membrane t-SNARE; involved in fusion of secretory vesicles at the plasma membrane; syntaxin homolog that is functionally redundant with Sso1; *SSO2* has a paralog, *SSO*1, that arose from the whole genome duplication.	+
SWC5	Component of the SWR1 complex; complex exchanges histone variant H2AZ (Htz1) for chromatin-bound histone H2A; protein abundance increases in response to DNA replication stress; relocalizes to the cytosol in response to hypoxia.	++
TCD2	tRNA threonylcarbamoyladenosine dehydratase; required for the ct6A tRNA base modification, where an adenosine at position 37 is modified to form a cyclized active ester with an oxazolone ring; localized to the mitochondrial outer membrane; *TCD2* has a paralog, *TCD1*, that arose from the whole genome duplication.	++
TDA5	Putative protein of unknown function; detected in highly purified mitochondria in high-throughput studies; proposed to be involved in resistance to mechlorethamine and streptozotocin.	++
THO2	Subunit of the THO complex; *THO2* is required for efficient transcription elongation and involved in transcriptional elongation-associated recombination; required for LacZ RNA expression from certain plasmids.	+++
THP3	Protein that may have a role in transcription elongation; forms a complex with Csn12 that is recruited to transcribed genes; possibly involved in splicing based on pre-mRNA accumulation defect for many intron-containing genes.	++
TOP1	Topoisomerase I; nuclear enzyme that relieves torsional strain in DNA by cleaving and re-sealing the phosphodiester backbone; relaxes both positively and negatively supercoiled DNA; functions in replication, transcription, and recombination; role in processing ribonucleoside monophosphates in genomic DNA into irreversible single-strand breaks; enzymatic activity and interaction with Nsr1p are negatively regulated by polyphosphorylation.	++
TOP3	DNA Topoisomerase III; conserved protein that functions in a complex with Sgs1 and Rmi1 to relax single-stranded negatively-supercoiled DNA preferentially; DNA catenation/decatenation activity is stimulated by RPA and Sgs1-Top3-Rmi1; involved in telomere stability and regulation of mitotic recombination.	++

TRM1	tRNA methyltransferase; two forms of protein are made by alternative translation starts; localizes to both nucleus and mitochondrion to produce modified base N2,N2-dimethylguanosine in tRNAs in both compartments; nuclear Trm1 is evenly distributed around inner nuclear membrane in WT, but mislocalizes as puncta near ER-nucleus junctions in spindle pole body (SPB) mutants; both Trm1 inner nuclear membrane targeting and maintenance depend upon SPB.	+++
TRM10	tRNA methyltransferase; methylates the N-1 position of guanine at position 9 in tRNAs; protein abundance increases in response to DNA replication stress; member of the SPOUT (SpoU-TrmD) methyltransferase family; human ortholog *TRMT10A* plays a role in the pathogenesis of microcephaly and early onset diabetes; an 18-mer originates from the *TRM10* locus; genetic analysis shows the 18-mer is the translation regulator.	++
XRN1	Evolutionarily-conserved 5'-3' exonuclease; component of cytoplasmic processing (P) bodies involved in mRNA decay; also enters the nucleus and positively regulates transcription initiation and elongation; plays a role in microtubule-mediated processes, filamentous growth, ribosomal RNA maturation, and telomere maintenance; activated by the scavenger decapping enzyme Dcs1.	+++
YTA7	Protein that localizes to chromatin; has a role in regulation of histone gene expression; has a bromodomain-like region that interacts with the N-terminal tail of histone H3, and an ATPase domain; relocalizes to the cytosol in response to hypoxia; potentially phosphorylated by Cdc28.	+

Cytoskeleton organization and morphogenesis

Gene	Function	Susceptibility level
ALF1	Alpha-tubulin folding protein; similar to mammalian cofactor B; Alf1p-GFP localizes to cytoplasmic microtubules; required for the folding of alpha-tubulin and may play an additional role in microtubule maintenance.	+++
ARC18	Subunit of the ARP2/3 complex; *ARP2/3* is required for the motility and integrity of cortical actin patches.	++
ARP1	Actin-related protein of the dynactin complex; required for spindle orientation and nuclear migration; forms actin-like short filament composed of 9 or 10 Arp1 monomers.	++
AVO2	Component of a complex containing the Tor2pkinase and other proteins; complex may have a role in regulation of cell growth.	+++
BEM1	Protein containing SH3-domains; involved in establishing cell polarity and morphogenesis; functions as a scaffold protein for complexes that include Cdc24, Ste5, Ste20, and Rsr1.	+++
BEM2	Rho GTPase activating protein (RhoGAP); involved in the control of cytoskeleton organization and cellular morphogenesis; required for bud emergence.	++
BEM4	Protein involved in establishment of cell polarity and bud emergence; interacts with the Rho1p small GTP-binding protein and with the Rho-type GTPase Cdc42; involved in maintenance of proper telomere length.	+++

BUB3	Kinetochore checkpoint WD40 repeat protein; localizes to kinetochores during prophase and metaphase, delays anaphase in the presence of unattached kinetochores; forms complexes with Mad1-Bub1 and with Cdc20, binds Mad2 and Mad3; functions at kinetochore to activate APC/C-Cdc20 for normal mitotic progression.	++
KEL1	Protein required for proper cell fusion and cell morphology; forms a complex with Bud14 and Kel2 that regulates Bnr1 (formin) to affect actin cable assembly, cytokinesis, and polarized growth.	+
MDY2	Protein involved in inserting tail-anchored proteins into ER membranes; forms a complex with Get4; required for efficient mating; involved in shmoo formation and nuclear migration in the pre-zygote; associates with ribosomes.	++
MMR1	Phosphorylated protein of the mitochondrial outer membrane; localizes only to mitochondria of the bud; interacts with Myo2 to mediate mitochondrial distribution to buds; mRNA is targeted to the bud via the transport system involving She2.	+
NCL1	S-adenosyl-L-methionine-dependent tRNA: m5C-methyltransferase; methylates cytosine to m5C at several positions in tRNAs and intron-containing pre-tRNAs; increases proportion of tRNALeu(CAA) with m5C at wobble position in response to hydrogen peroxide, causing selective translation of mRNA from genes enriched in TTG codon; loss of *NCL1* confers hypersensitivity to oxidative stress; similar to Nop2 and human proliferation associated nucleolar protein p120.	++
RTN1	Reticulon protein; involved in nuclear pore assembly and maintenance of tubular ER morphology.	+
RVS161	Amphiphysin-like lipid raft protein; N-BAR domain protein that interacts with Rvs167p and regulates polarization of the actin cytoskeleton, endocytosis, cell polarity, cell fusion and viability following starvation or osmotic stress.	+
SAC6	Fimbrin, actin-bundling protein; cooperates with Scp1 in organization and maintenance of the actin cytoskeleton; phosphorylated by Cdc28/Clb2 in metaphase on T103, to regulate conformation, and modulate actin filament binding affinity and actin cable dynamics; relocalizes from the plasma membrane to the cytoplasm upon DNA replication stress; human homologs *PLS3* and *LCP1* implicated in spinocerebellar ataxia type 2 (SCA2) can each complement yeast null mutant.	+++
SHS1	Component of the septin ring that is required for cytokinesis; present at the ends of rod-like septin hetero-oligomers; C-terminal extension is important for recruitment of Bni5 to the mother-bud neck, which in turn is required for Myo1 recruitment and cytokinesis; undergoes sumoylation and phosphorylation during mitosis; protein abundance increases in response to DNA replication stress.	+++
SLA1	Cytoskeletal protein binding protein; required for assembly of the cortical actin cytoskeleton; interacts with proteins regulating actin dynamics and proteins required for endocytosis; found in the nucleus and cell cortex; has 3 SH3 domains.	+++

SPC72	Gamma-tubulin small complex (gamma-TuSC) receptor; recruits the gamma-TuSC complex to the cytoplasmic side of the SPB, connecting nuclear microtubules to the SPB; involved in astral microtubule formation, stabilization, and with Stu2, anchoring astral MTs at the cytoplasmic face of the SPB, and regulating plus-end MT dynamics; regulated by Cdc5 kinase.	++
SSD1	Translational repressor with a role in polar growth and wall integrity; regulated by Cbk1 phosphorylation to effect bud-specific translational control and localization of specific mRNAs; interacts with TOR pathway components; contains a functional N-terminal nuclear localization sequence and nucleocytoplasmic shuttling appears to be critical to Ssd1 function.	++
VAC17	Phosphoprotein involved in vacuole inheritance; degraded in late M phase of the cell cycle; acts as a vacuole-specific receptor for myosin Myo2.	+
VRP1	Verprolin, proline-rich actin-associated protein; involved in cytoskeletal organization and cytokinesis; promotes actin nucleation and endocytosis; related to mammalian Wiskott-Aldrich syndrome protein (WASP)-interacting protein (WIP).	+++

DNA repair

Gene	Function	Susceptibility level
CTF3	Outer kinetochore protein that forms a complex with Mcm16 and Mcm22; may bind the kinetochore to spindle microtubules; required for the spindle assembly checkpoint; orthologous to human centromere constitutive-associated network (CCAN) subunit CENP-I and fission yeast *mis6*.	+
FZO1	Mitofusin; integral membrane protein involved in mitochondrial outer membrane tethering and fusion; role in mitochondrial genome maintenance; efficient tethering and degradation of Fzo1p requires an intact N-terminal GTPase domain; targeted for destruction by the ubiquitin ligase SCF-Mdm30p and the cytosolic ubiquitin-proteasome system.	+++
MET18	Component of cytosolic iron-sulfur protein assembly (CIA) machinery; acts at a late step of Fe-S cluster assembly; forms the CIA targeting complex with Cia1 and Cia2 that directs Fe-S cluster incorporation into a subset of proteins involved in methionine biosynthesis, DNA replication and repair, transcription, and telomere maintenance; ortholog of human *MMS19*.	+
MIP1	Mitochondrial DNA polymerase gamma; single subunit of mitochondrial DNA polymerase in yeast, in contrast to metazoan complex of catalytic and accessory subunits; polymorphic in yeast, petites occur more frequently in some lab strains; human ortholog *POLG* complements yeast *mip1* mutant; mutations in human *POLG* associated with Alpers-Huttenlocher syndrome (AHS), progressive external ophthalmoplegia (PEO), parkinsonism, other mitochondrial diseases.	++
MLH1	Protein required for mismatch repair in mitosis and meiosis; also required for crossing over during meiosis; forms a complex with Pms1 and Msh2-Msh3 during mismatch repair; human homolog is associated with hereditary non-polyposis colon cancer.	++

MTF1	Mitochondrial RNA polymerase specificity factor; has structural similarity to S-adenosylmethionine-dependent methyltransferases and functional similarity to bacterial sigma-factors; Mtf1 interacts with and stabilizes the Rpo41-promoter complex, enhancing DNA bending and melting to facilitate pre-initiation open complex formation.	++
MTF2	Mitochondrial protein that interacts with mitochondrial RNA polymerase; interacts with an N-terminal region of mitochondrial RNA polymerase (Rpo41) and couples RNA processing and translation to transcription.	+

Ion homeostasis

Gene	Function	Susceptibility level
FRE8	Protein with sequence similarity to iron/copper reductases; involved in iron homeostasis; deletion mutant has iron deficiency/accumulation growth defects.	+
ICE2	Integral ER membrane protein with type-III transmembrane domains; required for maintenance of ER zinc homeostasis; necessary for efficient targeting of Trm1 tRNA methyltransferase to inner nuclear membrane; mutations cause defects in cortical ER morphology in both the mother and daughter cells.	+++
LSO2	Protein with a potential role in response to iron deprivation; localizes to nucleus and cytoplasm, and nuclear localization is enhanced under iron-replete conditions; null mutant exhibits slow growth during iron deprivation.	++
TRK1	Component of the Trk1-Trk2 potassium transport system; 180 kDa high affinity potassium transporter; phosphorylated *in vivo* and interacts physically with the phosphatase Ppz1, suggesting Trk1 activity is regulated by phosphorylation.	++

Internal pH homeostasis

Gene	Function	Susceptibility level
VMA1	Subunit A of the V1 peripheral membrane domain of V-ATPase; protein precursor undergoes self-catalyzed splicing to yield the extein Tfp1 and the intein Vde (PI-SceI), which is a site-specific endonuclease; the V1 peripheral membrane domain of the vacuolar H+-ATPase (V-ATPase) has eight subunits; involved in methionine restriction extension of chronological lifespan in an autophagy-dependent manner.	+++
VMA10	Subunit G of the V1 peripheral membrane domain of V-ATPase; part of the electrogenic proton pump found throughout the endomembrane system; involved in vacuolar acidification; the V1 peripheral membrane domain of the vacuolar H+-ATPase (V-ATPase) has eight subunits.	+++

VMA13	Subunit H of the V1 peripheral membrane domain of V-ATPase; part of the electrogenic proton pump found throughout the endomembrane system; serves as an activator or a structural stabilizer of the V-ATPase; the V1 peripheral membrane domain of the vacuolar H+-ATPase (V-ATPase) has eight subunits.	+++
VMA16	Subunit c'' of the vacuolar ATPase; v-ATPase functions in acidification of the vacuole; one of three proteolipid subunits of the V0 domain.	+++
VMA21	Integral membrane protein required for V-ATPase function; not an actual component of the vacuolar H+-ATPase (V-ATPase) complex; diverged ortholog of human *XMEA* (X-linked Myopathy with Excessive Autophagy); functions in the assembly of the V-ATPase; localized to the yeast endoplasmic reticulum.	+++
VMA5	Subunit C of the V1 peripheral membrane domain of V-ATPase; part of the electrogenic proton pump found throughout the endomembrane system; required for the V1 domain to assemble onto the vacuolar membrane; the V1 peripheral membrane domain of vacuolar H+-ATPase (V-ATPase) has eight subunits.	+++
VMA9	Vacuolar H+ ATPase subunit e of the V-ATPase V0 subcomplex; essential for vacuolar acidification; interacts with the V-ATPase assembly factor Vma21p in the ER; involved in V0 biogenesis.	+++
VPH2	Integral membrane protein required for V-ATPase function; not an actual component of the vacuolar H+-ATPase (V-ATPase) complex; functions in the assembly of the V-ATPase; localized to the endoplasmic reticulum (ER); involved in methionine restriction extension of chronological lifespan in an autophagy-dependent manner.	++

Intracellular trafficking

Gene	Function	Susceptibility level
ARL3	ARF-like small GTPase of the RAS superfamily; required for recruitment of Arl1, a GTPase that regulates membrane traffic, to the Golgi apparatus; NatC-catalyzed N-terminal acetylation regulates Golgi membrane association mediated by interaction with membrane receptor, Sys1; similar to ADP-ribosylation factor and orthologous to mammalian *ARFRP1.*	+
ARV1	Cortical ER protein; implicated in the membrane insertion of tail-anchored C-terminal single transmembrane domain proteins; may function in transport of glycosylphosphatidylinositol intermediates into the ER lumen; required for normal intracellular sterol distribution; human *ARV1,* required for normal cholesterol and bile acid homeostasis, can complement yeast *arv1* null mutant; human variant causing early onset epileptic encephalopathy is unable to rescue the yeast null.	++
AVL9	Conserved protein involved in exocytic transport from the Golgi; mutation is synthetically lethal with *apl2 vps1* double mutation; member of a protein superfamily with orthologs in diverse organisms.	+

CHS3	Chitin synthase III; catalyzes the transfer of N-acetylglucosamine (GlcNAc) to chitin; required for synthesis of the majority of cell wall chitin, the chitin ring during bud emergence, and spore wall chitosan; contains overlapping di-leucine and di-acidic signals that mediate, respectively, intracellular trafficking by AP-1 and trafficking to plasma membrane by exomer complex.	+
CHS5	Component of the exomer complex; the exomer which also contains Csh6p, Bch1p, Bch2p, and Bud7, is involved in the export of select proteins, such as chitin synthase Chs3p, from the Golgi to the plasma membrane.	+
DID2	Class E protein of the vacuolar protein-sorting (Vps) pathway; binds Vps4 and directs it to dissociate ESCRT-III complexes; forms a functional and physical complex with Ist1p; human ortholog may be altered in breast tumors.	++
DID4	Class E Vps protein of the ESCRT-III complex; required for sorting of integral membrane proteins into lumenal vesicles of multivesicular bodies, and for delivery of newly synthesized vacuolar enzymes to the vacuole, involved in endocytosis.	++
DOA1	WD repeat protein required for ubiquitin-mediated protein degradation; ubiquitin binding cofactor that complexes with Cdc48; required for ribophagy; controls cellular ubiquitin concentration; promotes efficient NHEJ in postdiauxic/stationary phase; facilitates N-terminus-dependent proteolysis of centromeric histone H3 (Cse4) for faithful chromosome segregation.	+
DOA4	Ubiquitin hydrolase; deubiquitinates intralumenal vesicle (ILVs) cargo proteins; required for recycling ubiquitin from proteasome-bound ubiquitinated intermediates, acts at the late endosome/prevacuolar compartment to recover ubiquitin from ubiquitinated membrane proteins destined for the vacuole; *DOA4* has a paralog, *UBP5*, that arose from the whole genome duplication.	+++
DRS2	Trans-golgi network aminophospholipid translocase (flippase); maintains membrane lipid asymmetry in post-Golgi secretory vesicles; contributes to clathrin-coated vesicle formation, endocytosis, protein trafficking between the Golgi and endosomal system and the cellular response to mating pheromone; autoinhibited by its C-terminal tail; localizes to the trans-Golgi network; mutations in human homolog *ATP8B1* result in liver disease.	++
END3	EH domain-containing protein involved in endocytosis; actin cytoskeletal organization and cell wall morphogenesis; forms a complex with Sla1 and Pan1.	+++
EPS1	ER protein with chaperone and co-chaperone activity; involved in retention of resident ER proteins; has a role in recognizing proteins targeted for ER-associated degradation (ERAD), member of the protein disulfide isomerase family.	++
FYV10	Subunit of GID complex; involved in proteasome-dependent catabolite inactivation of gluconeogenic enzymes FBPase, PEPCK, and c-MDH; forms dimer with Rmd5 that is then recruited to GID Complex by Gid8; contains a degenerate RING finger motif needed for GID complex ubiquitin ligase activity in vivo, as well as CTLH and CRA domains; plays role in anti-apoptosis; required for survival upon exposure to K1 killer toxin.	+

GCS1	ADP-ribosylation factor GTPase activating protein (ARF GAP); involved in ER-Golgi transport; required for prospore membrane formation; regulates phospholipase Spo14; shares functional similarity with Glo3; *GCS1* has a paralog, *SPS18*, that arose from the whole genome duplication.	+++
GET1	Subunit of the GET complex; involved in insertion of proteins into the ER membrane; required for the retrieval of HDEL proteins from the Golgi to the ER in an *ERD2* dependent fashion and for normal mitochondrial morphology and inheritance.	+
GTR2	Subunit of a TORC1-stimulating GTPase complex; subunit of the Gtr1-Gtr2 GTPase complex that stimulates TORC1 in response to amino acid stimulation; stimulates the GTPase activity of Gtr1p; negatively regulates the Ran/Tc4 GTPase cycle; activates transcription; tethered to the vacuolar membrane as part of the EGO complex (EGOC); required for sorting of Gap1p; activated by the the Lst4-Lst7 GAP complex; localizes to cytoplasm and to chromatin.	++
GYP1	Cis-golgi GTPase-activating protein (GAP) for yeast Rabs; the Rab family members are Ypt1 (*in vivo*) and for Ypt1, Sec4, Ypt7, and Ypt51 (*in vitro*); involved in vesicle docking and fusion.	++
HSE1	Subunit of the endosomal Vps27-Hse1 complex; complex is required for sorting of ubiquitinated membrane proteins into intralumenal vesicles prior to vacuolar degradation, as well as for recycling of Golgi proteins and formation of lumenal membranes.	+++
PKH2	Serine/threonine protein kinase; involved in sphingolipid-mediated signaling pathway that controls endocytosis; activates Ypk1 and Ykr2, components of signaling cascade required for maintenance of cell wall integrity; contains a PH-like domain; redundant with Pkh1p; *PKH2* has a paralog, *PKH1*, that arose from the whole genome duplication.	++
RAV1	Subunit of RAVE complex (Rav1, Rav2, Skp1); the RAVE complex promotes assembly of the V-ATPase holoenzyme; required for transport between the early and late endosome/PVC and for localization of TGN membrane proteins; potential Cdc28 substrate.	++
RAV2	Subunit of RAVE complex (Rav1, Rav2, Skp1); the RAVE complex associates with the V1 domain of the vacuolar membrane (H+)-ATPase (V-ATPase) and promotes assembly and reassembly of the holoenzyme.	++
RER1	Protein involved in retention of membrane proteins; including Sec12, in the ER; localized to Golgi.	+
RGP1	Subunit of a Golgi membrane exchange factor (Ric1-Rgp1); this complex catalyzes nucleotide exchange on Ypt6.	+++
SEC22	R-SNARE protein; assembles into SNARE complex with Bet1p, Bos1 and Sed5; cycles between the ER and Golgi complex; involved in anterograde and retrograde transport between the ER and Golgi.	+
SEC28	Epsilon-COP subunit of the coatomer; regulates retrograde Golgi-to-ER protein traffic; stabilizes Cop1, the alpha-COP and the coatomer complex; non-essential for cell growth; protein abundance increases in response to DNA replication stress.	++

SEC66	Non-essential subunit of Sec63 complex; with Sec61 complex, Kar2/BiP and Lhs1 forms a channel competent for SRP-dependent and post-translational SRP-independent protein targeting and import into the ER; other members are Sec63, Sec62, and Sec72.	+++
SLM4	Component of the EGO and GSE complexes; essential for integrity and function of EGO; EGO is involved in the regulation of microautophagy and GSE is required for proper sorting of amino acid permease Gap1; gene exhibits synthetic genetic interaction with *MSS4.*	++
SLM5	Mitochondrial asparaginyl-tRNA synthetase.	++
SNF2	Catalytic subunit of the SWI/SNF chromatin remodeling complex; involved in transcriptional regulation; contains DNA-stimulated ATPase activity; functions interdependently in transcriptional activation with Snf5p and Snf6p.	+
SNF7	One of four subunits of the ESCRT-III complex; involved in the sorting of transmembrane proteins into the multivesicular body (MVB) pathway; recruited from the cytoplasm to endosomal membranes; ESCRT-III stands for endosomal sorting complex required for transport III.	+++
SNF8	Component of the ESCRT-II complex; ESCRT-II is involved in ubiquitin-dependent sorting of proteins into the endosome; appears to be functionally related to *SNF7*; involved in glucose derepression.	+++
STP22	Component of the ESCRT-I complex; ESCRT-I is involved in ubiquitin-dependent sorting of proteins into the endosome; prevents polyubiquitination of the arrestin-related protein Rim8p, thereby directing its monoubiquitination by Rsp5; homologous to the mouse and human Tsg101 tumor susceptibility gene; mutants exhibit a Class E Vps phenotype.	+++
TIM18	Component of the mitochondrial *TIM22* complex; involved in insertion of polytopic proteins into the inner membrane; may mediate assembly or stability of the complex.	++
TLG2	Syntaxin-like t-SNARE; forms a complex with Tlg1 and Vti1 and mediates fusion of endosome-derived vesicles with the late Golgi; required along with *VPS45* for an early step of the constitutive CVT pathway.	+
TRS33	Core component of TRAPP complexes I, II and IV; transport protein particle (TRAPP) complexes are related multimeric guanine nucleotide-exchange factor for the GTPase Ypt1, regulating ER-Golgi traffic (TRAPPI), intra-Golgi traffic (TRAPPII), endosome-Golgi traffic (TRAPPII and III) and autophagy (TRAPPIII, and IV); proposed subunit of a novel complex, TRAPPIV, that may function redundantly with TRAPPIII as a GEF that activates Ypt1 during autophagy.	++
TRS85	Component of transport protein particle (TRAPP) complex III; TRAPPIII is a multimeric guanine nucleotide-exchange factor for the GTPase Ypt1, regulating endosome-Golgi traffic and required for membrane expansion during autophagy and the CVT pathway; directs Ypt1 to the PAS; late post-replication meiotic role.	++
VAM10	Protein involved in vacuole morphogenesis; acts at an early step of homotypic vacuole fusion that is required for vacuole tethering.	++

VAM3	Syntaxin-like vacuolar t-SNARE; functions with Vam7 in vacuolar protein trafficking; mediates docking/fusion of late transport intermediates with the vacuole; has an acidic di-leucine sorting signal and C-terminal transmembrane region.	++
VAM6	Guanine nucleotide exchange factor for the GTPase Gtr1; subunit of the HOPS endocytic tethering complex; vacuole membrane protein; functions as a Rab GTPase effector, interacting with both GTP- and GDP-bound conformations of Ypt7; facilitates tethering and promotes membrane fusion events at the late endosome and vacuole; required for both membrane and protein trafficking; component of vacuole-mitochondrion contacts (vCLAMPs) important for lipid transfer between organelles.	++
VPS1	Dynamin-like GTPase required for vacuolar sorting; also involved in actin cytoskeleton organization, endocytosis, late Golgi-retention of some proteins, regulation of peroxisome biogenesis.	+++
VPS16	Subunit of the HOPS and the CORVET complexes; part of the Class C Vps complex essential for membrane docking and fusion at Golgi-to-endosome and endosome-to-vacuole protein transport stages.	++
VPS17	Subunit of the membrane-associated retromer complex; essential for endosome-to-Golgi retrograde protein transport; peripheral membrane protein that assembles onto the membrane with Vps5 to promote vesicle formation; required for recruiting the retromer complex to the endosome membranes.	++
VPS21	Endosomal Rab family GTPase; required for endocytic transport and sorting of vacuolar hydrolases; required for endosomal localization of the CORVET complex; required with *YPT52* for MVB biogenesis and sorting; involved in autophagy and ionic stress tolerance; geranylgeranylation required for membrane association; protein abundance increases in response to DNA replication stress; mammalian Rab5 homolog; *VPS21* has a paralog, *YPT53*, that arose from the whole genome duplication.	++
VPS24	One of four subunits of the ESCRT-III complex; forms an endosomal sorting complex required for transport III (ESCRT-III) subcomplex with Did4; involved in the sorting of transmembrane proteins into the multivesicular body (MVB) pathway.	+++
VPS25	Component of the ESCRT-II complex; ESCRT-II is involved in ubiquitin-dependent sorting of proteins into the endosome.	++
VPS27	Endosomal protein that forms a complex with Hse1; required for recycling Golgi proteins, forming lumenal membranes and sorting ubiquitinated proteins destined for degradation; has Ubiquitin Interaction Motifs which bind ubiquitin (Ubi4).	++
VPS28	Component of the ESCRT-I complex; complex is involved in ubiquitin-dependent sorting of proteins into the endosome; conserved C-terminal domain interacts with ESCRT-III subunit Vps20p; other members include Stp22, Srn2, Vps28, and Mvb12.	+++
VPS33	ATP-binding protein that is a subunit of the HOPS and CORVET complexes; essential for protein sorting, vesicle docking, and fusion at the vacuole; binds to SNARE domain.	+++

VPS36	Component of the ESCRT-II complex; contains the GLUE (GRAM Like Ubiquitin binding in EAP45) domain which is involved in interactions with ESCRT-I and ubiquitin-dependent sorting of proteins into the endosome; plays a role in the formation of mutant huntingtin (Htt) aggregates in yeast.	+++
VPS4	AAA-ATPase involved in multivesicular body (MVB) protein sorting; ATP-bound Vps4p localizes to endosomes and catalyzes ESCRT-III disassembly and membrane release; ATPase activity is activated by Vta1; regulates cellular sterol metabolism.	+
VPS41	Subunit of the HOPS endocytic tethering complex; vacuole membrane protein that functions as a Rab GTPase effector, interacting specifically with the GTP-bound conformation of Ypt7p, facilitating tethering, docking and promoting membrane fusion events at the late endosome and vacuole; required for both membrane and protein trafficking; Yck3-mediated phosphorylation regulates the organization of vacuolar fusion sites.	++
VPS5	Nexin-1 homolog; required for localizing membrane proteins from a prevacuolar/late endosomal compartment back to late Golgi; structural component of retromer membrane coat complex; forms a retromer subcomplex with Vps17; required for recruiting the retromer complex to the endosome membranes; *VPS5* has a paralog, *YKR078W*, that arose from the whole genome duplication.	++
VPS52	Component of the GARP (Golgi-associated retrograde protein) complex; GARP is required for the recycling of proteins from endosomes to the late Golgi, and for mitosis after DNA damage induced checkpoint arrest; involved in localization of actin and chitin; members of the GARP complex are Vps51-Vps52-Vps53-Vps54.	++
VPS61	Dubious open reading frame; unlikely to encode a functional protein, based on available experimental and comparative sequence data; not conserved in closely related Saccharomyces species; 4% of ORF overlaps the verified gene *RGP1*; deletion causes a vacuolar protein sorting defect.	+++
VPS63	Putative protein of unknown function; not conserved in closely related *Saccharomyces species*; 98% of ORF overlaps the verified gene *YPT6*; deletion causes a vacuolar protein sorting defect; decreased levels of protein in enolase deficient mutant.	+++
VPS74	Golgi phosphatidylinositol-4-kinase effector and PtdIns4P sensor; interacts with the cytosolic domains of cis and medial glycosyltransferases, and in the PtdIns4P-bound state mediates the targeting of these enzymes to the Golgi; interacts with the catalytic domain of Sac1, the major cellular PtdIns4P phosphatase, to direct dephosphosphorylation of the Golgi pool of PtdIns4P; tetramerization required for function; ortholog of human *GOLPH3/GPP34/GMx33*.	++
YPT6	Rab family GTPase; required for endosome-to-Golgi, intra-Golgi retrograde, and retrograde Golgi-to-ER transport; temporarily at the Golgi, dissociating into the cytosol on arrival of the late Golgi GTPase Ypt32; Golgi-localized form is GTP bound, while cytosolic form is GDP-bound; required for delivery of Atg9 to the phagophore assembly site during autophagy under heat stress, with Ypt6p for starvation induced autophagy and for the CVT pathway.	+++

Lipid synthesis

Gene	Function	Susceptibility level
ELO2	Fatty acid elongase, involved in sphingolipid biosynthesis; acts on fatty acids of up to 24 carbons in length; mutations have regulatory effects on 1,3-beta-glucan synthase, vacuolar ATPase, and the secretory pathway; *ELO2* has a paralog, *ELO1*, that arose from the whole genome duplication; lethality of the *elo2 elo3* double null mutation is functionally complemented by human *ELOVL1* and weakly complemented by human *ELOVL3* or *ELOV7*.	+++
ELO3	Elongase; involved in fatty acid and sphingolipid biosynthesis; synthesizes very long chain 20-26-carbon fatty acids from C18-CoA primers; involved in regulation of sphingolipid biosynthesis; lethality of the *elo2 elo3* double null mutation is functionally complemented by human *ELOVL1* and weakly complemented by human *ELOVL3* or *ELOV7*.	++
ERG2	C-8 sterol isomerase; catalyzes isomerization of delta-8 double bond to delta-7 position at an intermediate step in ergosterol biosynthesis; transcriptionally down-regulated when ergosterol is in excess.	+++
ERG24	C-14 sterol reductase; acts in ergosterol biosynthesis; mutants accumulate the abnormal sterol ignosterol (ergosta-8,14 dienol), and are viable under anaerobic growth conditions but inviable on rich medium under aerobic condition.	++
ERG28	Endoplasmic reticulum membrane protein; may facilitate protein-protein interactions between the Erg26 dehydrogenase and the Erg27p 3-ketoreductase and/or tether these enzymes to the ER, also interacts with Erg6.	+++
ERG3	C-5 sterol desaturase; glycoprotein that catalyzes the introduction of a C-5(6) double bond into episterol, a precursor in ergosterol biosynthesis; transcriptionally down-regulated when ergosterol is in excess; mutants are viable, but cannot grow on non-fermentable carbon sources; substrate of HRD ubiquitin ligase; mutation is functionally complemented by human *SC5D.*	++
ERG4	C-24(28) sterol reductase; catalyzes the final step in ergosterol biosynthesis; mutants are viable, but lack ergosterol.	+
ERG5	C-22 sterol desaturase; a cytochrome P450 enzyme that catalyzes the formation of the C-22(23) double bond in the sterol side chain in ergosterol biosynthesis; may be a target of azole antifungal drugs	+
ETR1	2-enoyl thioester reductase; member of the medium chain dehydrogenase/reductase family; localized to mitochondria, where it has a probable role in fatty acid synthesis; human MECR functionally complements the respiratory growth defect of the null mutant.	++
HTD2	Mitochondrial 3-hydroxyacyl-thioester dehydratase; involved in fatty acid biosynthesis, required for respiratory growth and for normal mitochondrial morphology.	++

KCS1	Inositol hexakisphosphate and inositol heptakisphosphate kinase; generation of high energy inositol pyrophosphates by Kcs1 is required for many processes such as vacuolar biogenesis, stress response, RNA polymerase I-mediated rRNA transcription and telomere maintenance; inositol hexakisphosphate is also known as IP6; inositol heptakisphosphate is also known as IP7.	+++
LAM1	Putative sterol transfer protein; localizes to puncta in the cortical ER; probable role in retrograde transport of sterols from the plasma membrane to the ER; one of six StART-like domain-containing proteins in yeast that may be involved in sterol transfer between intracellular membranes.	+
LIP5	Protein involved in biosynthesis of the coenzyme lipoic acid.	+
LOA1	Lysophosphatidic acid acyltransferase; involved in triacelglyceride homeostasis and lipid droplet formation; localized to lipid droplets and the ER; specificity for oleoyl-CoA.	+
PDX3	Pyridoxine (pyridoxamine) phosphate oxidase; has homologs in *E. coli* and *Myxococcus xanthus*; transcription is under the general control of nitrogen metabolism.	+++
SAC1	Phosphatidylinositol phosphate (PtdInsP) phosphatase; involved in hydrolysis of PtdIns[4]P in the early and medial Golgi; regulated by interaction with Vps74p; ER localized transmembrane protein which cycles through the Golgi; involved in protein trafficking and processing, secretion, and cell wall maintenance; regulates sphingolipid biosynthesis through the modulation of PtdIns(4)P metabolism.	++
SPO7	Putative regulatory subunit of Nem1-Spo7 phosphatase holoenzyme; regulates nuclear growth by controlling phospholipid biosynthesis, required for normal nuclear envelope morphology, premeiotic replication, and sporulation.	+

Mitochondrial function

Gene	Function	Susceptibility level
AEP1	Protein required for expression of the mitochondrial *OLI1* gene; mitochondrial *OLI1* gene encodes subunit 9 of F1-F0 ATP synthase.	++
AIF1	Mitochondrial cell death effector; translocates to the nucleus in response to apoptotic stimuli, homolog of mammalian Apoptosis-Inducing Factor, putative reductase.	++
ATP1	Alpha subunit of the F1 sector of mitochondrial F1-F0 ATP synthase; which is a large, evolutionarily conserved enzyme complex required for ATP synthesis; F1 translationally regulates *ATP6* and *ATP8* expression to achieve a balanced output of ATP synthase genes encoded in nucleus and mitochondria; phosphorylated; N-terminally propionylated *in vivo.*	+++
ATP10	Assembly factor for the F0 sector of mitochondrial F1-F0 ATP synthase; mitochondrial inner membrane protein; interacts genetically with *ATP6.*	+++
ATP11	Molecular chaperone; required for the assembly of alpha and beta subunits into the F1 sector of mitochondrial F1-F0 ATP synthase; N-terminally propionylated *in vivo.*	++

ATP23	Putative metalloprotease of the mitochondrial inner membrane; required for processing of Atp6; has an additional role in assembly of the F0 sector of the F1-F0 ATP synthase complex; substrate of the Mia40p-Erv1p disulfide relay system, and folding is assisted by Mia40.	++
CEM1	Mitochondrial beta-keto-acyl synthase; possible role in fatty acid synthesis; required for mitochondrial respiration; human homolog *OXSM* can complement yeast *cem1* null mutant.	+++
COA3	Mitochondrial protein required for cytochrome c oxidase assembly; also involved in translational regulation of Cox1 and prevention of Cox1 aggregation before assembly; located in the mitochondrial inner membrane.	+++
COQ1	Hexaprenyl pyrophosphate synthetase; catalyzes the first step in ubiquinone (coenzyme Q) biosynthesis.	++
COQ3	O-methyltransferase; catalyzes two different O-methylation steps in ubiquinone (Coenzyme Q) biosynthesis; component of a mitochondrial ubiquinone-synthesizing complex; phosphoprotein.	+
COX5B	Subunit Vb of cytochrome c oxidase; cytochrome c oxidase is the terminal member of the mitochondrial inner membrane electron transport chain; Cox5B is predominantly expressed during anaerobic growth while its isoform Va (Cox5A) is expressed during aerobic growth.	+
COX13	Subunit VIa of cytochrome c oxidase; present in a subclass of cytochrome c oxidase complexes that may have a role in mimimizing generation of reactive oxygen species; not essential for cytochrome c oxidase activity but may modulate activity in response to ATP	+
COX17	Copper metallochaperone that transfers copper to Sco1 and Cox11; eventual delivery to cytochrome c oxidase; contains twin cysteine-x9-cysteine motifs; interacts with the MICOS complex, and interaction is promoted by copper ions; human homolog *COX17* partially complements yeast null mutant.	++
COX18	Protein required for membrane insertion of C-terminus of Cox2; mitochondrial integral inner membrane protein; interacts genetically and physically with Mss2 and Pnt1; similar to *S. cerevisiae* Oxa1, *N. crassa* Oxa2, and *E. coli YidC*; respiratory defect of the null mutant is functionally complemented by human *COX18* carrying the N-terminal 54 amino acids of *S. cerevisiae* Cox18.	+++
COX19	Protein required for cytochrome c oxidase assembly; located in the cytosol and mitochondrial intermembrane space; putative copper metallochaperone that delivers copper to cytochrome c oxidase; contains twin cysteine-x9-cysteine motifs.	+
COX6	Subunit VI of cytochrome c oxidase (Complex IV); Complex IV is the terminal member of the mitochondrial inner membrane electron transport chain; expression is regulated by oxygen levels.	++
FIS1	Protein involved in mitochondrial fission and peroxisome abundance; may have a distinct role in tethering protein aggregates to mitochondria in order to retain them in the mother cell; required for localization of Dnm1 and Mdv1 during mitochondrial division; mediates ethanol-induced apoptosis and ethanol-induced mitochondrial fragmentation.	+

FMC1	Mitochondrial matrix protein; required for assembly or stability at high temperature of the F1 sector of mitochondrial F1-F0 ATP synthase; null mutant temperature sensitive growth on glycerol is suppressed by multicopy expression of Odc1.	+++
GPD2	NAD-dependent glycerol 3-phosphate dehydrogenase; expression is controlled by an oxygen-independent signaling pathway required to regulate metabolism under anoxic conditions; located in cytosol and mitochondria.	+
GGC1	Mitochondrial GTP/GDP transporter; essential for mitochondrial genome maintenance; has a role in mitochondrial iron transport; member of the mitochondrial carrier family.	+++
MGM1	Mitochondrial GTPase, present in complex with Ugo1p and Fzo1p; required for mitochondrial morphology, fusion, and genome maintenance; promotes membrane bending.	+
MCX1	Non-proteolytic ATPase of the AAA family; stimulates incorporation of the pyridoxal phosphate cofactor into Hem1p (5-aminolevulinic acid synthase).	+
MDM10	Subunit of both the ERMES and the SAM complex; component of ERMES complex which acts as a molecular tether between the mitochondria and the ER, necessary for efficient phospholipid exchange between organelles and for mitophagy; SAM/TOB complex component that functions in the assembly of outer membrane beta-barrel proteins; involved in mitochondrial inheritance and morphology; ERMES complex is often co-localized with peroxisomes and concentrated areas of pyruvate dehydrogenase.	+
MDM12	Mitochondrial outer membrane protein, ERMES complex subunit; required for transmission of mitochondria to daughter cells; required for mitophagy; may influence import and assembly of outer membrane beta-barrel proteins; ERMES complex is often co-localized with peroxisomes and with concentrated areas of pyruvate dehydrogenase.	++
MDM20	Non-catalytic subunit of the NatB N-terminal acetyltransferase; NatB catalyzes N-acetylation of proteins with specific N-terminal sequences; involved in mitochondrial inheritance and actin assembly.	++
MDM34	Mitochondrial component of the ERMES complex; links the ER to mitochondria and may promote inter-organellar calcium and phospholipid exchange as well as coordinating mitochondrial DNA replication and growth; required for mitophagy; ERMES complex is often co-localized with peroxisomes and with concentrated areas of pyruvate dehydrogenase.	++
MEF2	Mitochondrial elongation factor involved in translational elongation.	++
MHR1	Mitochondrial ribosomal protein of the large subunit; also involved in homologous recombination in mitochondria; required for recombination-dependent mtDNA partitioning; involved in stimulation of mitochondrial DNA replication in response to oxidative stress.	++
MRS1	Splicing protein; required for splicing of two mitochondrial group I introns (BI3 in COB and AI5beta in *COX1*); forms a splicing complex, containing four subunits of Mrs1 and two subunits of the BI3-encoded maturase, that binds to the BI3 RNA; *MRS1* has a paralog, *CCE1*, that arose from the whole genome duplication.	++

MSF1	Mitochondrial phenylalanyl-tRNA synthetase; active as a monomer, unlike the cytoplasmic subunit which is active as a dimer complexed to a beta subunit dimer; similar to the alpha subunit of *E. coli* phenylalanyl-tRNA synthetase.	+++
MSK1	Mitochondrial lysine-tRNA synthetase; required for import of both aminoacylated and deacylated forms of tRNA (Lys) into mitochondria and for aminoacylation of mitochondrially encoded tRNA (Lys).	+
NAM2	Mitochondrial leucyl-tRNA synthetase; also has direct role in splicing of several mitochondrial group I introns; indirectly required for mitochondrial genome maintenance; human homolog LARS2 can complement yeast null mutant and is implicated in Perrault syndrome.	++
OAR1	Mitochondrial 3-oxoacyl-[acyl-carrier-protein] reductase; may comprise a type II mitochondrial fatty acid synthase along with Mct1; human homolog *CBR4* complements yeast null mutant.	++
PET100	Chaperone that facilitates the assembly of cytochrome c oxidase; integral to the mitochondrial inner membrane; interacts with a subcomplex of subunits VII, VIIa, and VIII (Cox7, Cox9, and Cox8) but not with the holoenzyme.	++
PET117	Protein required for assembly of cytochrome c oxidase.	+
PET54	Mitochondrial inner membrane protein; binds to the 5' UTR of the COX3 mRNA to activate its translation together with Pet122 and Pet494p; also binds to the *COX1* Group I intron AI5 beta to facilitate exon ligation during splicing.	++
PRX1	Mitochondrial peroxiredoxin with thioredoxin peroxidase activity; has a role in reduction of hydroperoxides; reactivation requires Trr2 and glutathione; induced during respiratory growth and oxidative stress; phosphorylated; protein abundance increases in response to DNA replication stress.	++
PSD1	Phosphatidylserine decarboxylase of the mitochondrial inner membrane; converts phosphatidylserine to phosphatidylethanolamine; regulates mitochondrial fusion and morphology by affecting lipid mixing in the mitochondrial membrane and by influencing the ratio of long to short forms of Mgm1p; partly exposed to the mitochondrial intermembrane space; autocatalytically processed.	++
RRG9	Protein of unknown function; null mutant lacks mitochondrial DNA and cannot grow on glycerol; the authentic, non-tagged protein is detected in highly purified mitochondria in high-throughput studies.	++
RSM23	Mitochondrial ribosomal protein of the small subunit; has similarity to mammalian apoptosis mediator proteins; null mutation prevents induction of apoptosis by overproduction of metacaspase Mca1.	+++
RSM24	Mitochondrial ribosomal protein of the small subunit.	+
RTC6	Protein involved in translation; mutants have defects in biogenesis of nuclear ribosomes; sequence similar to prokaryotic ribosomal protein L36, may be a mitochondrial ribosomal protein; protein abundance increases in response to DNA replication stress.	++
SCO1	Copper-binding protein of mitochondrial inner membrane; required for cytochrome c oxidase activity and respiration; may function to deliver copper to cytochrome c oxidase.	+
SCO2	Protein anchored to mitochondrial inner membrane; may have a	+

	redundant function with Sco1p in delivery of copper to cytochrome c oxidase.	
SOV1	Mitochondrial protein of unknown function.	++
YTA12	Mitochondrial inner membrane m-AAA protease component; mediates degradation of misfolded or unassembled proteins; also required for correct assembly of mitochondrial enzyme complexes; overexpression of human *AFG3L2* complements respiratory defect of yeast *afg3 yta12* double null mutation, but overexpression of disease-associated *AFG3L2* variants does not; expression of both human *SPG7* (paraplegin) and *AFG3L2* complements yeast *yta12 afg3* double mutation.	+

Peroxisomal function

Gene	Function	Susceptibility level
PEX12	C3HC4-type RING-finger peroxin and E3 ubiquitin ligase; required for peroxisome biogenesis and peroxisomal matrix protein import.	+
PEX27	Peripheral peroxisomal membrane protein; involved in controlling peroxisome size and number.	+
PEX34	Protein that regulates peroxisome populations; peroxisomal integral membrane protein; interacts with Pex11p, Pex25p, and Pex27p to control both constitutive peroxisome division and peroxisome morphology and abundance during peroxisome proliferation.	+
PEX19	Chaperone and import receptor for newly-synthesized class I PMPs; binds peroxisomal membrane proteins (PMPs) in the cytoplasm and delivers them to the peroxisome for subsequent insertion into the peroxisomal membrane.	+
PEX22	Putative peroxisomal membrane protein; required for import of peroxisomal proteins.	+
PEX6	AAA-peroxin; heterodimerizes with AAA-peroxin Pex1 and participates in the recycling of peroxisomal signal receptor Pex5 from the peroxisomal membrane to the cytosol; mutations in human *PEX6* can lead to severe peroxisomal disorders and early death.	++

Protein modification

Gene	Function	Susceptibility level
AIM22	Putative lipoate-protein ligase; required along with Lip2 and Lip5 for lipoylation of Lat1 and Kgd2; similar to E. coli LplA; null mutant displays reduced frequency of mitochondrial genome loss.	++
ARD1	Subunit of protein N-terminal acetyltransferase NatA; NatA comprises Nat1, Ard1, Nat5; acetylates many proteins to influence telomeric silencing, cell cycle, heat-shock resistance, mating, sporulation, early stages of mitophagy; protein abundance increases under DNA replication stress.	+++

BNI5	Linker protein responsible for recruitment of myosin to the bud neck; interacts with the C-terminal extensions of septins Cdc11 and Shs1 and binds Myo1p to promote cytokinesis.	++
BRE5	Ubiquitin protease cofactor; forms deubiquitination complex with Ubp3 that coregulates anterograde and retrograde transport between the endoplasmic reticulum and Golgi compartments; null is sensitive to brefeldin A.	++
CDC73	Component of the Paf1 complex; binds to and modulates the activity of RNA polymerases I and II; required for expression of certain genes, modification of some histones, and telomere maintenance; involved in transcription elongation as demonstrated by the G-less-based run-on (GLRO) assay; protein abundance increases in response to DNA replication stress; human homolog, parafibromin, is a tumour suppressor linked to breast, renal and gastric cancer.	++
CTK1	Catalytic (alpha) subunit of C-terminal domain kinase I (CTDK-I); phosphorylates both RNA pol II subunit Rpo21 to affect transcription and pre-mRNA 3' end processing, and ribosomal protein Rps2 to increase translational fidelity; required for H3K36 trimethylation but not dimethylation by Set2; suggested stimulatory role in 80S formation during translation initiation.	+++
DBF2	Ser/Thr kinase involved in transcription and stress response; functions as part of a network of genes in exit from mitosis; localization is cell cycle regulated; activated by Cdc15 during the exit from mitosis; also plays a role in regulating the stability of *SWI5* and *CLB2* mRNAs; phosphorylates Chs2 to regulate primary septum formation and Hof1 to regulate cytokinesis.	+++
EFM4	Lysine methyltransferase; involved in the dimethylation of *eEF1A* (Tef1/Tef2) at lysine 316; sequence similarity to S-adenosylmethionine-dependent methyltransferases of the seven beta-strand family; role in vesicular transport.	+
EFT2	Elongation factor 2 (EF-2), also encoded by *EFT1*; catalyzes ribosomal translocation during protein synthesis; contains diphthamide, the unique posttranslationally modified histidine residue specifically ADP-ribosylated by diphtheria toxin; *EFT2* has a paralog, *EFT1*, that arose from the whole genome duplication.	+
FES1	Hsp70 (Ssa1) nucleotide exchange factor; required for the release of misfolded proteins from the Hsp70 system to the Ub-proteasome machinery for destruction; cytosolic homolog of Sil1, which is the nucleotide exchange factor for BiP (Kar2) in the endoplasmic reticulum; protein abundance increases in response to DNA replication stress.	+++
FPK1	Ser/Thr protein kinase; phosphorylates several aminophospholipid translocase family members, regulating phospholipid translocation and membrane asymmetry; phosphorylates and inhibits upstream inhibitory kinase, Ypk1; localizes to the cytoplasm, early endosome/TGN compartments and thplasma membrane.	+
GIM3	Subunit of the heterohexameric cochaperone prefoldin complex; prefoldin binds specifically to cytosolic chaperonin and transfers target proteins to it; prefoldin complex also localizes to chromatin of actively transcribed genes in the nucleus and facilitates transcriptional elongation.	++

HPM1	AdoMet-dependent methyltransferase; involved in a novel 3-methylhistidine modification of ribosomal protein Rpl3p; seven beta-strand MTase family member	+
KEX2	Kexin, a subtilisin-like protease (proprotein convertase); a calcium-dependent serine protease involved in the activation of proproteins of the secretory pathway.	+++
LIP2	Lipoyl ligase; involved in the modification of mitochondrial enzymes by the attachment of lipoic acid groups.	+
MCK1	Dual-specificity ser/thr and tyrosine protein kinase; roles in chromosome segregation, meiotic entry, genome stability, phosphorylation-dependent protein degradation (Rcn1 and Cdc6), inhibition of protein kinase A, transcriptional regulation, inhibition of RNA pol III, calcium stress and inhibition of Clb2-Cdc28 after nuclear division; *MCK1* has a paralog, *YGK3*, that arose from the whole genome duplication.	+
NAS2	Evolutionarily conserved 19S regulatory particle assembly-chaperone; involved in assembly of the base subcomplex of the 19S proteasomal regulatory particle (RP); non-essential gene; interacts with Rpn4; protein abundance increases in response to DNA replication stress; ortholog of human p27.	++
NAT1	Subunit of protein N-terminal acetyltransferase NatA; NatA comprised of Nat1, Ard1, and Nat5; N-terminally acetylates many proteins to influence multiple processes such as cell cycle progression, heat-shock resistance, mating, sporulation, telomeric silencing and early stages of mitophagy; orthologous to human *NAA15*; expression of both human *NAA10* and *NAA15* functionally complements *ard1 nat1* double mutant although single mutations are not complemented by their orthologs.	+++
NAT3	Catalytic subunit of the NatB N-terminal acetyltransferase; NatB catalyzes acetylation of the amino-terminal methionine residues of all proteins beginning with Met-Asp or Met-Glu and of some proteins beginning with Met-Asn or Met-M.	+++
NAT4	N alpha-acetyl-transferase; involved in acetylation of the N-terminal residues of histones H4 and H2A.	+++
OST3	Gamma subunit of the oligosaccharyltransferase complex of the ER lumen; complex catalyzes asparagine-linked glycosylation of newly synthesized proteins.	+
OST4	Subunit of the oligosaccharyltransferase complex of the ER lumen; complex catalyzes protein asparagine-linked glycosylation; type I membrane protein required for incorporation of Ost3 or Ost6 into the OST complex.	+++
PMT1	Protein O-mannosyltransferase of the ER membrane; transfers mannose from dolichyl phosphate-D-mannose to protein serine and threonine residues.	+
PPM1	Carboxyl methyltransferase; methylates the C terminus of the protein phosphatase 2A catalytic subunit (Pph21 or Pph22), which is important for complex formation with regulatory subunits; required for methionine to inhibit autophagy and promote growth.	++
PPT2	Phosphopantetheine:protein transferase (PPTase); activates mitochondrial acyl carrier protein (Acp1) by phosphopantetheinylation.	++
PTK2	Serine/threonine protein kinase; involved in regulation of ion transport across plasma membrane; carboxyl terminus is essential for glucose-dependent Pma1 activation via	++

	phosphorylation of Pma1-Ser899.	
RAM1	Beta subunit of the CAAX farnesyltransferase (FTase); this complex prenylates the a-factor mating pheromone and Ras proteins; required for the membrane localization of Ras proteins and a-factor; homolog of the mammalian FTase beta subunit.	+
SHR5	Palmitoyltransferase subunit; this complex adds a palmitoyl lipid moiety to heterolipidated substrates such as Ras1 and Ras2 through a thioester linkage; palmitoylation is required for Ras2 membrane localization; Palmitoyltransferase is composed of Shr5 and Erf.	+++
SWM1	Subunit of the anaphase-promoting complex (APC); APC is an E3 ubiquitin ligase that regulates the metaphase-anaphase transition and exit from mitosis; required for activation of the daughter-specific gene expression and spore wall maturation.	+++
TOS3	Protein kinase; related to and functionally redundant with Elm1 and Sak1p for the phosphorylation and activation of Snf1; functionally orthologous to *LKB1*, a mammalian kinase associated with Peutz-Jeghers cancer-susceptibility syndrome; *TOS3* has a paralog, *SAK1*, that arose from the whole genome duplication.	++
UBA4	E1-like protein that activates Urm1 before urmylation; also acts in thiolation of the wobble base of cytoplasmic tRNAs by adenylating and then thiolating Urm1; receives sulfur from Tum1.	++
UBI4	Ubiquitin; becomes conjugated to proteins, marking them for selective degradation via the ubiquitin-26S proteasome system.	+
UBP14	Ubiquitin-specific protease; specifically disassembles unanchored ubiquitin chains; involved in fructose-1,6-bisphosphatase (Fbp1) degradation	+
UBP6	Ubiquitin-specific protease; situated in the base subcomplex of the 26S proteasome, releases free ubiquitin from branched polyubiquitin chains *en bloc*, rather than from the distal tip of the chain.	+
VMS1	Component of a Cdc48-complex involved in protein quality control; exhibits cytosolic and ER-membrane localization, with Cdc48p, during normal growth, and contributes to ER-associated degradation (ERAD) of specific substrates at a step after their ubiquitination; forms a mitochondrially-associated complex with Cdc48 and Npl4 under oxidative stress that is required for ubiquitin-mediated mitochondria-associated protein degradation (MAD); conserved in *C. elegans* and humans.	++
YME1	Catalytic subunit of i-AAA protease complex; complex is located in mitochondrial inner membrane; responsible for degradation of unfolded or misfolded mitochondrial gene products; serves as nonconventional translocation motor to pull PNPase into intermembrane space; also has role in intermembrane space protein folding; mutation causes elevated rate of mitochondrial turnover.	++

Protein synthesis

Gene	Function	Susceptibility level
HCR1	eIF3j component of translation initiation factor 3 (eIF3); dual function protein involved in translation initiation as a substoichiometric component (eIF3j) of eIF3; required for 20S pre-rRNA processing; required at post-transcriptional step for efficient retrotransposition; absence decreases Ty1 Gag:GFP protein levels.	+++
IFM1	Mitochondrial translation initiation factor.	++
MRP17	Mitochondrial ribosomal protein of the small subunit; *MRP17* exhibits genetic interactions with *PET122*, encoding a *COX3*-specific translational activator.	++
MRP7	Mitochondrial ribosomal protein of the large subunit.	++
MRPL13	Mitochondrial ribosomal protein of the large subunit; not essential for mitochondrial translation.	+
MRPL20	Mitochondrial ribosomal protein of the large subunit.	++
MRPL22	Mitochondrial ribosomal protein of the large subunit.	++
MRPL24	Mitochondrial ribosomal protein of the large subunit; two mitochondrial ribosomal proteins, *YmL14* and *YmL24*, have been assigned to the same gene.	+
MRPL36	Mitochondrial ribosomal protein of the large subunit; overproduction suppresses mutations in the *COX2* leader peptide-encoding region.	++
MRPL49	Mitochondrial ribosomal protein of the large subunit.	++
MRPS12	Mitochondrial protein; may interact with ribosomes based on co-purification experiments; similar to *E. coli* and human mitochondrial S12 ribosomal proteins.	++
MRX14	Putative mitochondrial ribosomal protein of the large subunit; similar to E. coli L34 ribosomal protein; required for respiratory growth, as are most mitochondrial ribosomal proteins; protein increases in abundance and relocalizes to the plasma membrane upon DNA replication stress.	+
RML2	Mitochondrial ribosomal protein of the large subunit (L2); has similarity to *E. coli* L2 ribosomal protein; mutant allele (*fat21*) causes inability to utilize oleate, and induce oleic acid oxidation; may interfere with activity of the Adr1 transcription factor.	+++
RPB4	RNA polymerase II subunit B32; forms dissociable heterodimer with Rpb7; Rpb4/7 dissociates from RNAPII as Ser2 CTD phosphorylation increases; Rpb4/7 regulates cellular lifespan via mRNA decay process; involved in recruitment of 3'-end processing factors to transcribing RNAPII complex, export of mRNA to cytoplasm under stress conditions; also involved in translation initiation.	+++
RPL20B	Ribosomal 60S subunit protein L20B; homologous to mammalian ribosomal protein L18A, no bacterial homolog; *RPL20B* has a paralog, *RPL20A*, that arose from the whole genome duplication.	+++
RPL21A	Ribosomal 60S subunit protein L21A.	+

RPL21B	Ribosomal 60S subunit protein L21B; homologous to mammalian ribosomal protein L21, no bacterial homolog; *RPL21B* has a paralog, *RPL21A*, that arose from the whole genome duplication.	+
RPL39	Ribosomal 60S subunit protein L39; required for ribosome biogenesis; loss of both Rpl31p and Rpl39p confers lethality; also exhibits genetic interactions with *SIS1* and *PAB*1; homologous to mammalian ribosomal protein L39, no bacterial homolog.	+++
RPL40A	Ubiquitin-ribosomal 60S subunit protein L40A fusion protein; cleaved to yield ubiquitin and ribosomal protein L40A; ubiquitin may facilitate assembly of the ribosomal protein into ribosomes; homologous to mammalian ribosomal protein L40, no bacterial homolog; *RPL40A* has a paralog, *RPL40B*, that arose from the whole genome duplication; relative distribution to the nucleus increases upon DNA replication stress.	+
RPL42B	Ribosomal 60S subunit protein L42B; required for propagation of the killer toxin-encoding M1 double-stranded RNA satellite of the L-A double-stranded RNA virus; homologous to mammalian ribosomal protein L36A, no bacterial homolog; *RPL42B* has a paralog, RPL42A.	++
RPP1A	Ribosomal stalk protein P1 alpha; involved in the interaction between translational elongation factors and the ribosome; free (non-ribosomal) P1 stimulates the phosphorylation of the *eIF2* alpha subunit (Sui2) by Gcn2; accumulation of P1 in the cytoplasm is regulated by phosphorylation and interaction with the P2 stalk component.	++
RPS10A	Protein component of the small (40S) ribosomal subunit; homologous to mammalian ribosomal protein S10, no bacterial homolog; *RPS10A* has a paralog, *RPS10B*, that arose from the whole genome duplication; mutations in the human homolog associated with Diamond-Blackfan anemia.	+
RPS11B	Protein component of the small (40S) ribosomal subunit; homologous to mammalian ribosomal protein S11 and bacterial S17; *RPS11B* has a paralog, *RPS11A*, that arose from the whole genome duplication.	+
RPS16A	Protein component of the small (40S) ribosomal subunit	+
RPS21A	Protein component of the small (40S) ribosomal subunit; homologous to mammalian ribosomal protein S21, no bacterial homolog.	+
RPS21B	Protein component of the small (40S) ribosomal subunit; homologous to mammalian ribosomal protein S21, no bacterial homolog; *RPS21B* has a paralog, *RPS21*A, that arose from the whole genome duplication.	++
RPS24A	Protein component of the small (40S) ribosomal subunit; homologous to mammalian ribosomal protein S24, no bacterial homolog; *RPS24A* has a paralog, *RPS24B*, that arose from the whole genome duplication.	+
RPS27B	Protein component of the small (40S) ribosomal subunit; homologous to mammalian ribosomal protein S27, no bacterial homolog; *RPS27B* has a paralog, *RPS27*A, that arose from the whole genome duplication.	+++
RPS6A	Protein component of the small (40S) ribosomal subunit; homologous to mammalian ribosomal protein S6, no bacterial homolog; phosphorylated on S233 by Ypk3 in a TORC1-dependent manner, and on S232 in a TORC1/2-dependent manner by Ypk1/2/3; *RPS6A* has a paralog, *RPS6B*, that arose from the whole genome duplication.	++

RPS6B	Protein component of the small (40S) ribosomal subunit;	+
RSM19	Mitochondrial ribosomal protein of the small subunit; has similarity to *E. coli* S19 ribosomal protein.	++
RSM26	Mitochondrial ribosomal protein of the small subunit.	+
SEM1	19S proteasome regulatory particle lid subcomplex component; role in Ub-dependent proteolysis and proteasome stability; involved in TREX-2 mediated mRNA export, and in the prevention of transcription-associated genome instability; ubiquitinated by Nedd4-like E3-ligase, Rsp5; human ortholog *DSS1*, a *BRCA1* binding protein implicated in cancer, complements the yeast null; drives trinucleotide repeat expansion; protein abundance increases in response to DNA replication stress.	+
TIF3	Translation initiation factor eIF-4B; contains an RNA recognition motif and binds to single-stranded RNA; has RNA annealing activity; interacts with Rps20 at the head of the 40S ribosomal subunit and alters the structure of the mRNA entry channel.	++
TIF4631	Translation initiation factor *eIF4G*; subunit of the mRNA cap-binding protein complex (eIF4F) that also contains eIF4E (Cdc33); interacts with Pab1 and with eIF4A (Tif1).	+

Response to stress

Gene	Function	Susceptibility level
CCS1	Copper chaperone for superoxide dismutase Sod1; involved in oxidative stress protection; Met-X-Cys-X2-Cys motif within N-terminus is involved in insertion of copper into Sod1 under conditions of copper deprivation; required for regulation of yeast copper genes in response to DNA-damaging agents; protein abundance increases in response to DNA replication stress; human homolog CCS can complement yeast *ccs1* null mutant.	++
MXR2	Methionine-R-sulfoxide reductase; involved in the response to oxidative stress; protects iron-sulfur clusters from oxidative inactivation along with *MXR1*.	+
SYM1	Protein required for ethanol metabolism; induced by heat shock and localized to the inner mitochondrial membrane; homologous to mammalian peroxisomal membrane protein Mpv17; human homolog MPV17 is implicated in hepatocerebral mtDNA depletion syndromes (MDDS), and complements yeast null mutant.	+
WHI2	Protein required for full activation of the general stress response; required with binding partner Psr1, possibly through Msn2 dephosphorylation; regulates growth during the diauxic shift.	+
WHI5	Repressor of G1 transcription; binds to SCB binding factor (SBF) at SCB target promoters in early G1.	+

Signal transduction

Gene	Function	Susceptibility level

GPB2	Multistep regulator of cAMP-PKA signaling; inhibits PKA downstream of Gpa2 and Cyr1, thereby increasing cAMP dependency; inhibits Ras activity through direct interactions with Ira1/2.	+
IRS4	EH domain-containing protein; involved in regulating phosphatidylinositol 4,5-bisphosphate levels and autophagy; Irs4 and Tax4 bind and activate the PtdIns phosphatase Inp51; Irs4 and Tax4 are involved in localizing Atg17 to the PAS; *IRS4* has a paralog.	++
NBP2	Protein involved in the HOG (high osmolarity glycerol) pathway; negatively regulates Hog1 by recruitment of phosphatase Ptc1 the Pbs2-Hog1 complex; interacts with Bck1 and down regulates the cell wall integrity pathway; found in the nucleus and cytoplasm, contains an SH3 domain and a Ptc1 binding domain (PBM).	++
PDE2	High-affinity cyclic AMP phosphodiesterase; component of the cAMP-dependent protein kinase signaling system, protects the cell from extracellular cAMP	+
RAS2	GTP-binding protein; regulates nitrogen starvation response, sporulation, and filamentous growth; farnesylation and palmitoylation required for activity and localization to plasma membrane; homolog of mammalian Ras proto-oncogenes; *RAS2* has a paralog, *RAS1*, that arose from the whole genome duplication.	+++
REI1	Cytoplasmic pre-60S factor; required for the correct recycling of shuttling factors Alb1, Arx1 and Tif6 at the end of the ribosomal large subunit biogenesis; involved in bud growth in the mitotic signaling network.	++
SIP1	Alternate beta-subunit of the Snf1 kinase complex; may confer substrate specificity; vacuolar protein containing KIS (Kinase-Interacting Sequence) and ASC (Association with Snf1 kinase Complex) domains involved in protein interaction	+
SNF1	AMP-activated S/T protein kinase; forms a complex with Snf4 and members of the Sip1/Sip2/Gal83 family; required for transcription of glucose-repressed genes, thermotolerance, sporulation, and peroxisome biogenesis; regulates nucleocytoplasmic shuttling of Hxk2; regulates filamentous growth and acts as a non-canonical GEF, activating Arf3 during invasive growth; SUMOylation by Mms21 inhibits its function and targets Snf1 for destruction via the Slx5-Slx8 Ub ligase.	++
STE50	Adaptor protein for various signaling pathways; involved in mating response, invasive/filamentous growth, osmotolerance.	+
TUS1	Guanine nucleotide exchange factor (GEF) that modulates Rho1 activity; involved in the cell integrity signaling pathway; interacts with Rgl1; localization of Tus1 to the bed neck is regulated by Rgl1; multicopy suppressor of *tor2* mutation and *ypk1 ypk2* double mutation; potential Cdc28 substrate.	++

Transcription factors

Gene	Function	Susceptibility level
CBF1	Basic helix-loop-helix (bHLH) protein; forms homodimer to bind E-box consensus sequence CACGTG present at *MET* gene promoters and centromere DNA element I (CDEI); affects nucleosome positioning at this motif; associates with other transcription factors such as Met4 and Isw1 to mediate transcriptional activation or repression; associates with kinetochore proteins, required for chromosome segregation; protein abundance increases in response to DNA replication stress.	++
HAP5	Subunit of the Hap2/3/4/5 CCAAT-binding complex; complex is heme-activated and glucose repressed; complex is a transcriptional activator and global regulator of respiratory gene expression.	+
MGA2	ER membrane protein involved in regulation of *OLE1* transcription; inactive ER form dimerizes and one subunit is then activated by ubiquitin/proteasome-dependent processing followed by nuclear targeting; *MGA2* has a paralog, *SPT23*, that arose from the whole genome duplication.	++
OAF1	Oleate-activated transcription factor; subunit of a heterodimeric complex with Pip2, which binds to oleate-response elements (ORE) in the promoter of genes involved in beta-oxidation of fatty acids, peroxisome organization and biogenesis, activating transcription in the presence of oleate.	+
RPN4	Transcription factor that stimulates expression of proteasome genes; Rpn4 levels are in turn regulated by the 26S proteasome in a negative feedback control mechanism; RPN4 is transcriptionally regulated by various stress responses; relative distribution to the nucleus increases upon DNA replication stress.	+++
RSF2	Zinc-finger protein; involved in transcriptional control of both nuclear and mitochondrial genes, many of which specify products required for glycerol-based growth, respiration.	+
SFL1	Transcriptional repressor and activator; involved in repression of flocculation-related genes, and activation of stress responsive genes; has direct role in *INO1* transcriptional memory; negatively regulated by cAMP-dependent protein kinase A subunit Tpk2; premature stop codon (C1430T, Q477-stop) in SK1 background is linked to the aggressively invasive phenotype of SK1 relative to BY4741 (S288C).	++
SFP1	Regulates transcription of ribosomal protein and biogenesis genes; regulates response to nutrients and stress, G2/M transitions during mitotic cell cycle and DNA-damage response and modulates cell size; regulated by TORC1 and Mrs6; sequence of zinc finger, ChIP localization data, and protein-binding microarray (PBM) data, and computational analyses suggest it binds DNA directly at highly active RP genes and indirectly through Rap1 at others; can form the [ISP+] prion.	+++

STP1	Transcription factor; contains a N-terminal regulatory motif (RI) that acts as a cytoplasmic retention determinant and as an Asi dependent degron in the nucleus; undergoes proteolytic processing by SPS (Ssy1-Ptr3-Ssy5)-sensor component Ssy5 in response to extracellular amino acids; activates transcription of amino acid permease genes and may have a role in tRNA processing.	+
SWI6	Transcription cofactor; forms complexes with Swi4 and Mbp1 to regulate transcription at the G1/S transition; involved in meiotic gene expression; also binds Stb1 to regulate transcription at START; cell wall stress induces phosphorylation by Mpk1, which regulates Swi6 localization; required for the unfolded protein response, independently of its known transcriptional coactivators.	++
TUP1	General repressor of transcription; forms complex with Cyc8, involved in the establishment of repressive chromatin structure through interactions with histones H3 and H4.	+
UME6	Rpd3L histone deacetylase complex subunit; key transcriptional regulator of early meiotic genes; involved in chromatin remodeling and transcriptional repression via DNA looping; binds *URS1* upstream regulatory sequence, represses transcription by recruiting conserved histone deacetylase Rpd3 (through co-repressor Sin3) and chromatin-remodeling factor Isw2; couples metabolic responses to nutritional cues with initiation and progression of meiosis.	+++
UPC2	Sterol regulatory element binding protein; induces sterol biosynthetic genes, upon sterol depletion; acts as a sterol sensor, binding ergosterol in sterol rich conditions.	+
URE2	Nitrogen catabolite repression transcriptional regulator; inhibits *GLN3* transcription in good nitrogen source; role in sequestering Gln3 and Gat1 to the cytoplasm; has glutathione peroxidase activity and can mutate to acquire GST activity; self-assembly under limited nitrogen conditions creates [*URE3*] prion and releases catabolite repression.	+

Transporters

Gene	Function	Susceptibility level
DAL5	Allantoate permease; ureidosuccinate permease; also transports dipeptides, though with lower affinity than for allantoate and ureidosuccinate; expression is constitutive but sensitive to nitrogen catabolite repression.	+
FPS1	Aquaglyceroporin, plasma membrane channel; involved in efflux of glycerol and xylitol, and in uptake of acetic acid, arsenite, and antimonite; key factor in maintaining redox balance by mediating passive diffusion of glycerol; phosphorylated by Hog1 MAPK under acetate stress; deletion improves xylose fermentation; regulated by Rgc1 and Ask10, which are regulated by Hog1p phosphorylation under osmotic stress; phosphorylation by Ypk1 required to maintain an open state.	+++
MME1	Transporter of the mitochondrial inner membrane that exports magnesium; involved in mitochondrial Mg2+ homeostasis; has similarity to human mitochondrial ATP-Mg/Pi carriers.	++
NHA1	Na+/H+ antiporter; involved in sodium and potassium efflux through the plasma membrane; required for alkali cation tolerance at acidic pH.	+

OPT2	Oligopeptide transporter; localized to peroxisomes and affects glutathione redox homeostasis; also localizes to the plasma membrane (PM) and to the late Golgi, and has a role in maintenance of lipid asymmetry between the inner and outer leaflets of the PM; member of the OPT family, with potential orthologs in *S. pombe* and *C. albicans*; also plays a role in formation of mature vacuoles and in polarized cell growth.	+
SPF1	P-type ATPase, ion transporter of the ER membrane; required to maintain normal lipid composition of intracellular compartments and proper targeting of mitochondrial outer membrane tail-anchored proteins; involved in ER function and Ca2+ homeostasis; required for regulating Hmg2 degradation; confers sensitivity to a killer toxin (SMKT) produced by *Pichia farinosa* KK1.	+++
TPN1	Plasma membrane pyridoxine (vitamin B6) transporter; member of the purine-cytosine permease subfamily within the major facilitator superfamily; proton symporter with similarity to Fcy21, Fcy2, and Fcy22.	++
TPO1	Polyamine transporter of the major facilitator superfamily; member of the 12-spanner drug:H(+) antiporter *DHA1* family; recognizes spermine, putrescine, and spermidine; catalyzes uptake of polyamines at alkaline pH and excretion at acidic pH; during oxidative stress exports spermine, spermidine from the cell, which controls timing of expression of stress-responsive genes; phosphorylation enhances activity and sorting to the plasma membrane.	+
YIA6	Mitochondrial NAD+ transporter; involved in the transport of NAD+ into the mitochondria (see also *YEA6*); member of the mitochondrial carrier subfamily; disputed role as a pyruvate transporter; has putative mouse and human orthologs; *YIA6* has a paralog, *YEA6*, that arose from the whole genome duplication.	++

Unknown function

Gene	Function	Susceptibility level
AIM4	Protein proposed to be associated with the nuclear pore complex; null mutant is viable, displays elevated frequency of mitochondrial genome loss and is sensitive to freeze-thaw stress.	+
API2	Putative protein of unknown function; conserved among *S. cerevisiae* strains; not conserved in closely related *Saccharomyces* species; 26% of ORF overlaps the dubious ORF YDR524C-A	+
BIL1	Protein that binds Bud6 and has a role in actin cable assembly; involved in the Bnr1p-dependent pathway of cable assembly; localizes to bud tip and bud neck.	++
BUD26	Dubious open reading frame; unlikely to encode a functional protein, based on available experimental and comparative sequence data; not conserved in closely related Saccharomyces species; 1% of ORF overlaps the verified gene SNU56.	+
HGH1	Nonessential protein of unknown function.	++
IRC13	Putative protein of unknown function; conserved across *S. cerevisiae* strains.	+
JIP4	Protein of unknown function; previously annotated as two separate ORFs, YDR474C and YDR475C, which were merged as a result of corrections to the systematic reference sequence.	+

MRP8	Protein of unknown function; undergoes sumoylation; transcription induced under cell wall stress; protein levels are reduced under anaerobic conditions.	+
OPI10	Protein with a possible role in phospholipid biosynthesis; null mutant displays an inositol-excreting phenotype that is suppressed by exogenous choline; protein abundance increases in response to DNA replication stress.	++
OPI7	Dubious open reading frame; unlikely to encode a functional protein.	+
OPI9	Dubious open reading frame; unlikely to encode a functional protein.	+++
PAL1	Protein of unknown function thought to be involved in endocytosis.	+
RGL1	Regulator of Rho1p signaling, cofactor of Tus1; required for the localization of Tus1 during all phases of cytokinesis; green fluorescent protein (GFP)-fusion protein localizes to the bud neck and cytoplasm; null mutant is viable and exhibits growth defect on a non-fermentable (respiratory) carbon source.	++
RMD8	Cytosolic protein required for sporulation.	+
RRG1	Protein of unknown function; required for vacuolar acidification and mitochondrial genome maintenance; the authentic, non-tagged protein is detected in highly purified mitochondria in high-throughput studies.	+
SRP40	Nucleolar serine-rich protein; role in preribosome assembly or transport; may function as a chaperone of small nucleolar ribonucleoprotein particles (snoRNPs).	+
TPH3	Putative protein of unknown function; GFP-fusion protein localizes to the cytoplasm; contains two adjacent PH-like domains; conserved in closely related *Saccharomyces* species.	+
TVP15	Integral membrane protein; localized to late Golgi vesicles along with the v-SNARE Tlg2.	+
YAR029W	Member of *DUP240* gene family but contains no transmembrane domains; green fluorescent protein (GFP)-fusion protein localizes to the cytoplasm in a punctate pattern.	+
YBL071C-B	Putative protein of unknown function.	++
YBL094C	Dubious open reading frame; unlikely to encode a functional protein.	+
YBL100C	Dubious open reading frame; unlikely to encode a functional protein.	+++
YCL007C	Dubious open reading frame; unlikely to encode a functional protein.	+++
YCL026C	Deleted ORF; does not encode a protein.	+
YDR008C	Dubious open reading frame; unlikely to encode a functional protein.	+++
YDR203W	Dubious open reading frame; unlikely to encode a functional protein.	+
YDR442W	Dubious open reading frame; unlikely to encode a functional protein.	+++
YDR524C-B	Putative protein of unknown function.	++
YDR524W-C	Putative protein of unknown function.	++
YGL024W	Dubious open reading frame; unlikely to encode a functional protein.	+

YGL218W	Dubious open reading frame; unlikely to encode a functional protein.	++
YGR079W	Putative protein of unknown function.	+
YHR175W-A	Putative protein of unknown function.	++
YIL028W	Dubious open reading frame; unlikely to encode a functional protein.	++
YIL029C	Putative protein of unknown function.	++
YIL077C	Putative protein of unknown function.	+
YIL092W	Putative protein of unknown function.	+
YIL141W	Dubious open reading frame; unlikely to encode a functional protein.	+
YJR011C	Putative protein of unknown function.	+++
YJR114W	Dubious open reading frame; unlikely to encode a functional protein.	++
YKL118W	Dubious open reading frame; unlikely to encode a functional protein.	++
YKR004C-A	Merged open reading frame; does not encode a discreet protein; YKR004C-A was originally annotated as an independent ORF.	+
YLR202C	Dubious open reading frame; unlikely to encode a functional protein.	++
YLR235C	Dubious open reading frame; unlikely to encode a functional protein.	++
YLR264C-A	Putative protein of unknown function.	+
YLR297W	Protein of unknown function; green fluorescent protein (GFP)-fusion protein localizes to the vacuole.	+
YLR358C	Protein of unknown function.	+++
YLR407W	Putative protein of unknown function.	++
YMR001C-A	Putative protein of unknown function.	+
YNL190W	Hydrophilin essential in desiccation-rehydration process; cell wall protein; contains a putative GPI-attachment site.	++
YNL193W	Putative protein of unknown function.	+
YNL194C	Integral membrane protein; required for sporulation and plasma membrane sphingolipid content; similar to *SUR7*; GFP-fusion protein is induced in response to the DNA-damaging agent MMS; GFP-fusion protein is more abundant at MCCs (membrane compartment occupied by Can1) in the presence of glycerol and oleate; *YNL194C* has a paralog, *FMP45*, that arose from the whole genome duplication.	+
YOL050C	Dubious open reading frame; unlikely to encode a functional protein.	+
YOL107W	Putative protein of unknown function; green fluorescent protein (GFP)-fusion protein localizes to the cytoplasm and colocalizes in a punctate pattern with the early Golgi/COPI vesicles; *YOL107W* is not an essential protein.	++
YOR082C	Dubious open reading frame; unlikely to encode a functional protein.	+
YOR139C	Dubious open reading frame; unlikely to encode a functional protein.	++
YPL205C	Hypothetical protein; deletion of locus affects telomere length.	+
